# Direct Cell Radiolabeling for *in Vivo* Cell Tracking with PET and SPECT Imaging

**DOI:** 10.1021/acs.chemrev.1c00767

**Published:** 2022-05-12

**Authors:** Peter J. Gawne, Francis Man, Philip J. Blower, Rafael T. M. de Rosales

**Affiliations:** § School of Biomedical Engineering & Imaging Sciences, King’s College London, St Thomas’ Hospital, London, SE1 7EH, U.K.; ⊥ Institute of Pharmaceutical Science, School of Cancer and Pharmaceutical Sciences, King’s College London, London, SE1 9NH, U.K.

## Abstract

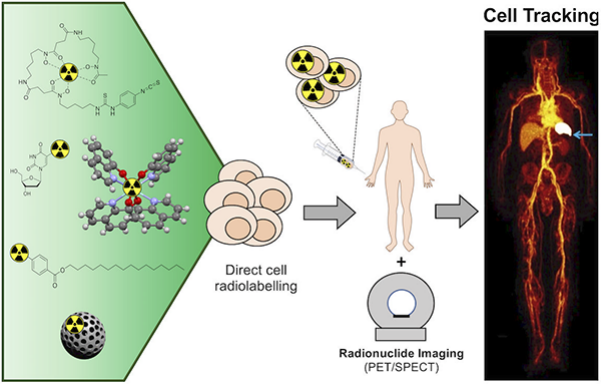

The arrival of cell-based therapies is a revolution in medicine. However, its safe clinical application in a rational manner depends on reliable, clinically applicable methods for determining the fate and trafficking of therapeutic cells in vivo using medical imaging techniques—known as in vivo cell tracking. Radionuclide imaging using single photon emission computed tomography (SPECT) or positron emission tomography (PET) has several advantages over other imaging modalities for cell tracking because of its high sensitivity (requiring low amounts of probe per cell for imaging) and whole-body quantitative imaging capability using clinically available scanners. For cell tracking with radionuclides, ex vivo direct cell radiolabeling, that is, radiolabeling cells before their administration, is the simplest and most robust method, allowing labeling of any cell type without the need for genetic modification. This Review covers the development and application of direct cell radiolabeling probes utilizing a variety of chemical approaches: organic and inorganic/coordination (radio)chemistry, nanomaterials, and biochemistry. We describe the key early developments and the most recent advances in the field, identifying advantages and disadvantages of the different approaches and informing future development and choice of methods for clinical and preclinical application.

## 1 Introduction

### 1.1 Cell Tracking: Preclinical and Clinical Applications

In vivo cell tracking describes the use of medical imaging techniques to allow the noninvasive visualization of the biodistribution and trafficking of active cells throughout a living organism. This information is highly beneficial for disease diagnosis (e.g., infection/inflammation), the imaging of biological mechanisms, and developing and evaluating the efficacy of cell-based treatments.^1^ Following several reports of toxicity and deaths associated with certain cellular therapy treatments in the clinic, it is essential to fully understand the biodistribution, accumulation, and tissue residence of therapeutic cells both during their preclinical development and in the clinical setting when treating patients.

Cell tracking has been extensively used in both preclinical and clinical studies. Notably, the in vivo tracking of autologous radiolabeled white blood cells for the diagnosis of inflammation and infection has been performed in patients for decades. More recently cell tracking has allowed noninvasive assessment of the fate of tumor cells in animal models, providing an invaluable tool to understand tumor development and metastasis, and supporting the assessment of antitumor therapies. Furthermore, cell tracking supports development and evaluation of cellular therapies (*e.g*. CAR T-cells, stem cells) by helping to answer the fundamental question: where do the cells go after administration? Significant developments have been made in recent years, particularly in T cell and stem cell engineering, that call for a variety of new and improved cell tracking methods to fully understand the biodistribution, accumulation, and tissue residence of therapeutic cells in preclinical and clinical settings.

There are a wide range of chemical methods and strategies to label cells for noninvasive in vivo cell tracking. These may be broadly categorized into indirect cell labeling and direct labeling methods, schematically represented in Figure 1. To choose the best approach for a specific application, it is important to have a clear understanding of their respective advantages and disadvantages. These will be summarized in the following section.

### 1.2 Direct Cell Labeling versus Indirect Cell Labeling

Indirect cell labeling usually requires genetic manipulation of the cells by stable transfection of a reporter gene. Reporter genes are used to induce the expression of proteins, such as cell receptors, transporters, or enzymes; imaging can then be performed by using contrast agents that specifically interact with these proteins (Figure 1A). A key benefit of indirect cell labeling is that the reporter gene protein is, ideally, present throughout the lifespan of the cell and is passed on during cell division. This allows in vivo imaging over a long period of time—potentially over the lifetime of the patient/subject—and if suitably calibrated, in principle provides information on the proliferation of the cells in vivo as well as their location. For long-term imaging, repeated administrations of the tracer are required. Additionally, some reporter genes can provide cell viability information as the corresponding protein does not function in a dead cell (e.g., the sodium-iodide symporter NIS is ATP-dependent and thus can only function in a live cell environment). Despite these advantages, the need for genetic manipulation of cells to allow imaging contrast is often seen as barrier to clinical translation, though this is less of an issue with cellular therapies that are already genetically modified during their development (e.g., CAR T-cells).^2^

By comparison, direct cell labeling (Figure 1B) is in principle a simpler cell tracking method as any chemical agent capable of entering cells or binding to cellular membranes can potentially be used for cell radiolabeling. Cells are usually labeled or “tagged” ex vivo/in vitro by incubation with the direct labeling agent, followed by injection into the subject. In vivo imaging can then be performed over time to assess the distribution of the cells. There are several methods for direct cell labeling. For example, uptake of the imaging probe can be mediated by phagocytosis or by the attachment to the cell membrane. These will be discussed further in section 4. It is important to note that since cells do not need to be modified genetically as a requirement for direct cell labeling, this method presents a lower regulatory barrier for clinical application compared to indirect methodologies. However, it does not allow imaging of cell proliferation, and can be restricted by the efflux of the labeling agent from cells over time, which can lead to reduction and misinterpretation of the imaging signal (Figure 1B).

Imaging modalities available for in vivo cell tracking vary greatly in properties, such as their spatial and temporal resolution, sensitivity (defined as the amount of contrast agent or label required to obtain sufficient imaging signal), field of view (FOV), and depth penetration. Thus, each modality comes with advantages and drawbacks. While in this Review we will focus on radionuclide-based imaging methods, to provide context the following subsection contains a brief overview of the other key imaging modalities used for cell tracking (Figure 2), with examples of cell labeling agents and their relevant pros and cons. Radionuclide imaging will then be discussed in more detail in detail in section 2.

### 1.3 Medical Imaging Techniques for Cell Tracking (Non-radionuclide Based)

#### 1.3.1 Magnetic Resonance Imaging (MRI)

Magnetic resonance imaging (MRI) is based on the spin characteristics and magnetic properties of atomic nuclei. Protons (^1^H) are the primary nuclei used for MRI contrast as they are abundant in water molecules within living systems. Imaging contrast in MRI is generated by the different longitudinal (*T*_1_) and transverse (*T*_2_) relaxation times of protons present in different tissues. Cell tracking with MRI requires exogenous imaging agents, which influence *T*_1_ and *T*_2_ of water protons or provide alternative spin-active nuclei and provide additional imaging contrast or allow “hotspot” imaging. Several agents containing paramagnetic metals (e.g., Gd^3+^ and Mn^2+/3+^), providing *T*_1_-weighted (positive) contrast, have been developed for both direct and indirect cell labeling.^9^ Additionally, superparamagnetic iron oxide nanoparticles (SPIONs), which provide *T*_2_-weighted (negative) or *T*_1_-based contrast depending on their properties, can be used to label cells via endocytic mechanisms.^10^ As well as imaging ^1^H, other spin-active nuclei such as ^19^F can be detected with MRI after administration of exogenous compounds (such as ^19^F-rich molecular compounds or nanoparticles) allowing “hotspot” MR imaging.^9,11^ While MRI as a modality provides exceptional spatial resolution (1–2 mm clinically) without the need for ionizing radiation, it suffers from its low sensitivity (typical in vivo contrast agent concentrations are 10^–3^–10^–5^ M) resulting in the need for large amounts of cell labeling agents to be administered (e.g., 10–30 pg Fe/cell clinically for SPIONs).^10^

#### 1.3.2 Magnetic Particle Imaging (MPI)

Magnetic particle imaging (MPI) is a relatively recent imaging modality, first introduced in 2005,^12^ allowing the direct imaging of SPIONs based on their magnetization in an external magnetic field. Several SPION-based MRI tracers have been repurposed as MPI tracers and, hence, have also been used for cell labeling and in vivo tracking with MPI.^13−15^ Cell tracking with MPI offers several benefits over MRI and other modalities. First, it benefits from a positive “hotspot” contrast with no endogenous signal from tissue. Additionally, it is highly sensitive, with the MPI signal being linearly quantitative with magnetic particle concentration, allowing calculation of the number of labeled cells.^14^ However, MPI suffers from a relatively low spatial resolution, compared to MRI, and it needs to be combined with an additional imaging modality to provide anatomical information. Furthermore, unlike MRI, CT, and nuclear imaging, there are currently no clinical MPI scanners available. Nonetheless, MPI remains a highly promising imaging modality for cell tracking.

#### 1.3.3 Computed Tomography (CT)

Computed tomography (CT) is a widely available medical imaging technique based on the differing levels of X-ray attenuation of tissues of varying density in the body resulting in imaging signal contrast. CT provides 3D images at high spatial resolution (∼0.1 mm preclinically and ∼0.5 mm clinically) and has practically unlimited depth penetration in tissues. However, the use of highly ionizing X-rays results in high radiation doses.^16^ While generally used for anatomical imaging, CT contrast can be generated by the administration of materials containing high *Z* elements (e.g., Au, I, Yb, Ba). In the context of cell tracking, gold nanoparticles are often the first choice to label cells because of their biocompatibility and favorable imaging contrast properties.^17,18^ However, as with MRI, the low sensitivity of CT cell tracking results in the need for high concentrations of contrast agent for in vivo detection that could lead to potential toxicity issues.

#### 1.3.4 Optical Imaging (OI)

Optical imaging (OI) is based on the detection of light emissions from molecules after their excitation, detected by external cameras that convert this signal into images. For preclinical in vivo applications, optical fluorescence imaging is often used. This relies on imaging agents consisting of exogenous chemical compounds that fluoresce after excitation by an external light source of a certain wavelength. A widely used alternative is bioluminescence imaging, where no excitation light is needed; instead, photons are generated by an endogenous chemical reaction, usually involving a reporter gene.^19^ In terms of cell labeling, reporter gene products such as fluorescent proteins (e.g., GFP, RFP) and luciferases (using luciferin) have been widely used for cell tracking with fluorescence and bioluminescence imaging, respectively. Alternatively, lipophilic optical dyes, such as 1,1′-dioctadecyl-3,3,3′,3′- tetramethylindodicarbocyanine (DiD) have been used to directly label cells for in vitro and in vivo cell imaging.^20^ OI techniques suffer from limited tissue penetration (a few mm, and up to a few cm in the near-infrared range) of both the excitation and emitted light, which affects sensitivity and spatial resolution, as well as significant tissue autofluorescence. Although the use of molecules emitting in the near-infrared is a partial remedy, this can limit in vivo cell tracking by optical imaging to the intraoperative and preclinical fields. Nonetheless, optical imaging is a highly sensitive technique compatible with light microscopy, making it an invaluable tool for the imaging of cells at multiple scales: from the whole-body to single-cell level.^21^

#### 1.3.5 Photoacoustic Imaging (PAI)

Photoacoustic (or optoacoustic) imaging (PAI) is based on the excitation of contrast agents or endogenous chromophores (e.g., oxyhemoglobin, deoxyhemoglobin, melanin) by externally applied light pulses. Upon relaxation, energy released as heat creates pressure waves that can be detected with an acoustic transducer.^22^ PAI is highly sensitive (in the pM range) and has submillimeter spatial resolution, It can penetrate several cm of tissue but suffers from a limited FOV. Despite this, because of the lower scattering of sound waves by tissue compared with photons, PAI has better depth penetration than standard OI techniques.^23^ Cell labeling and tracking with PAI has primarily been performed by loading cells with gold nanoparticles.^24^ More recent examples have performed cell labeling and tracking with organic semiconducting polymer nanoparticles capable of being excited in the second near-infrared region (NIR-II), which can mitigate depth penetration issues with PAI.^25^

## 2 Fundamentals of Radionuclide Imaging

**Figure 1:**
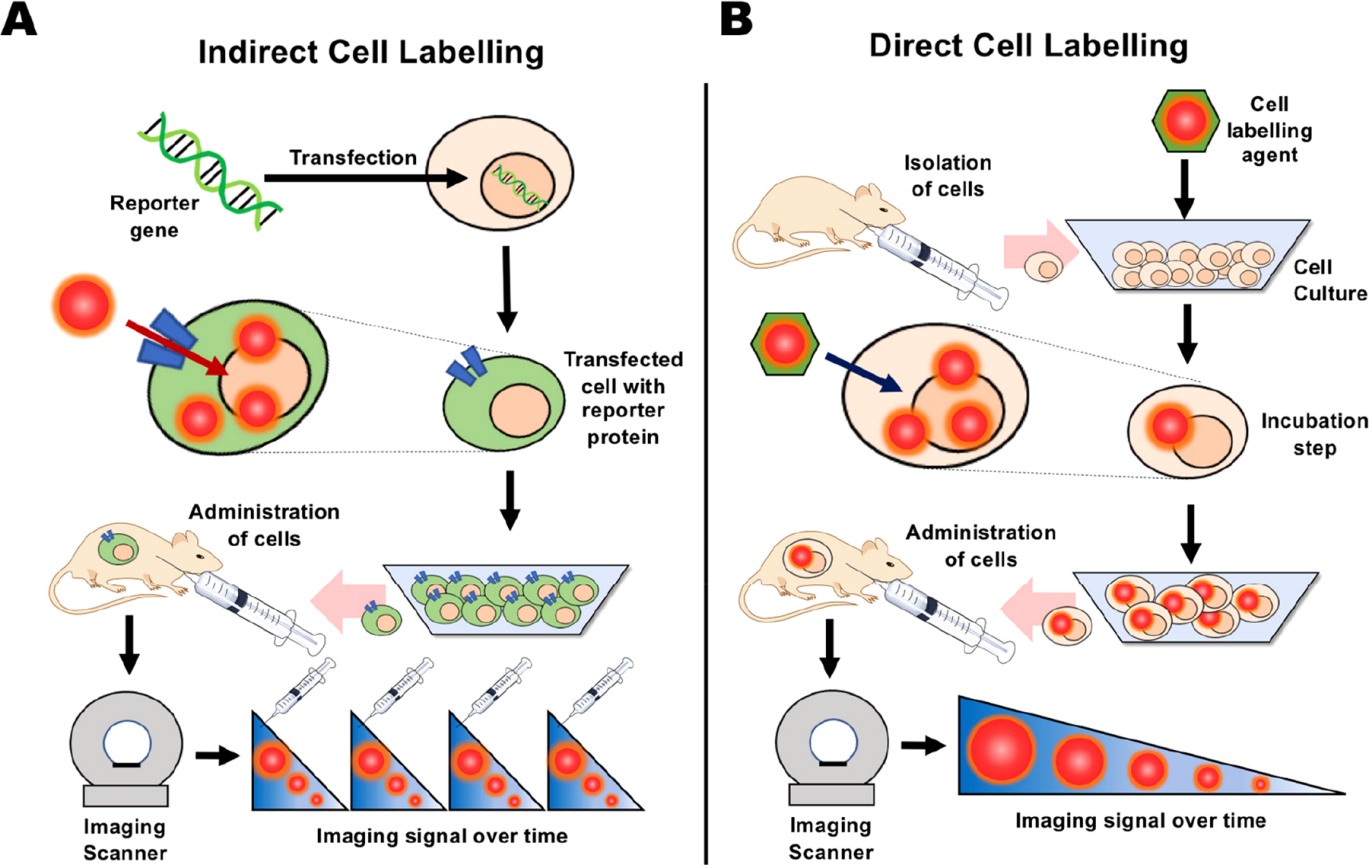
Principles of indirect and direct cell labeling used for cell tracking. (A) Indirect cell labeling. Cells are genetically modified with a reporter gene, enabling them to express a reporter protein, which allows binding or uptake of the imaging label in vivo. The cells can then be administered into the subject and imaged over time by repeated injections of imaging label that binds specifically to cells expressing the reporter gene. In principle, the gene expression persists over the lifespan of the cell and can be passed on to daughter cells. (B) Direct cell labeling. Cells are isolated from the subject, donor or culture and labeled in vitro. The labeled cells are then administered into the subject and can be imaged repeatedly for as long as the half-life of the imaging label allows (from hours to days).

### 2.1 Single Photon Emission Computed Tomography (SPECT) and Scintigraphy

Single photon emission computed tomography (SPECT) imaging utilizes gamma (γ) ray emitting radionuclides. The emitted γ rays have well-defined energy levels which are detected using a gamma camera, allowing the creation of a planar image, known as gamma scintigraphy. Alternatively, in SPECT imaging, a gamma camera is rotated around the imaging subject to capture the gamma emissions in 3D. To accurately determine the origin of the gamma ray photons, collimators are used to exclude diagonally incident photons. However, because of this exclusion the use of collimators reduces the fraction of gamma ray photons detected, resulting in a decrease in the imaging sensitivity (Figure 3A).

**Figure 2:**
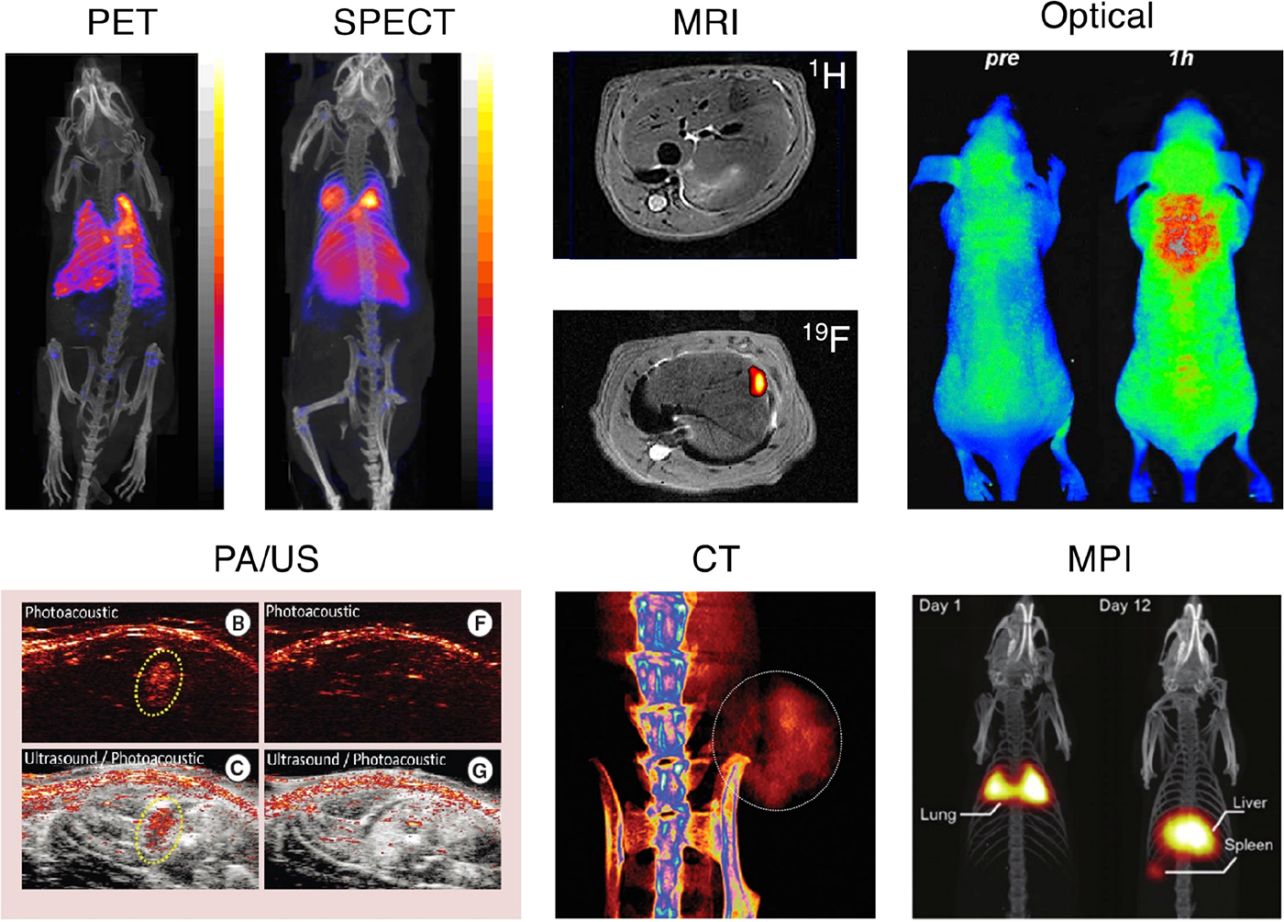
Representative images showing preclinical cell tracking studies with different imaging modalities and cell types, including nuclear imaging-based techniques with ^89^Zr- and ^111^In-labeled 5T33 cells (PET and SPECT) Reproduced with permission from ref (3). Copyright 2015, Springer Nature under CC License [https://creativecommons.org/licenses/by/4.0/]. MRI with SPIO- and ^19^F-labeled mesenchymal stromal cells. Reproduced with permission from ref (4). Copyright 2020, Springer Nature under CC License [https://creativecommons.org/licenses/by/4.0/]. Optical cell tracking of human hematopoietic cells. Reproduced with permission from ref (5). Copyright 2004, Springer Nature. Photoacoustic (PA) and ultrasound (US) cell tracking with gold nanoparticle-labeled cells. Reproduced with permission from ref (6). Copyright 2012, PLOS One under CC License [https://creativecommons.org/licenses/by/4.0/]. CT cell tracking of gold nanoparticle-labeled T cells. Reproduced with permission from ref (7). Copyright 2015, American Chemical Society. MPI cell tracking with SPIO labeled-stem cells. Reproduced with permission from ref (8). Copyright 2016, Springer Nature under CC License [https://creativecommons.org/licenses/by/4.0/].

**Figure 3:**
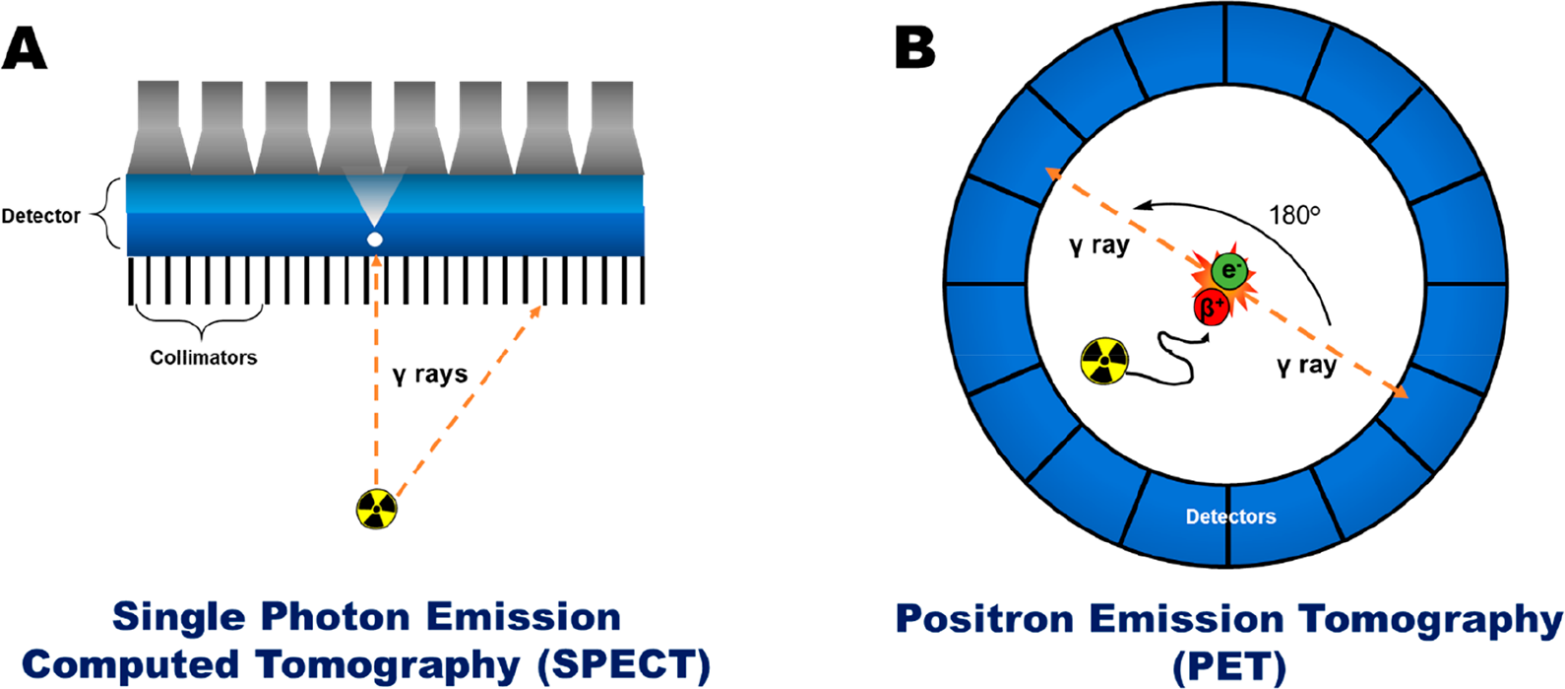
Schematic representation of (A) single photon emission computed tomography (SPECT) and (B) positron emission tomography (PET). The gamma camera depicted in A intrinsically produces a planar projection but by rotating the camera around the subject a three-dimensional tomographic reconstruction (SPECT scan) is produced. Adapted with permission from Man et al., ref (26). Copyright 2019 Man et al. Published by Elsevier under CC License [https://creativecommons.org/licenses/by/4.0/].

Several gamma-emitting radionuclides are available (Table 1) for radiolabeling a variety of different compounds, from small molecules and peptides to antibodies, nanoparticles and cells. In the clinic, the most widely used radionuclide is ^99m^Tc which offers a moderately short half-life (6 h, which is long enough for convenient synthesis of radiotracers while not imposing prolonged radiation exposure to the subject, but only allows tracking of cells for a few hours), favorable nuclear emission properties (89% γ radiation abundance at 140 keV) and convenient generator-based production.^27^ Because of its metallic character, ^99m^Tc radiotracers are based on the formation of coordination complexes between the radionuclide and a chelating agent. Another key SPECT radionuclide is ^111^In, which has a relatively long half-life (*t*_1/2_ = 2.81 d) allowing imaging over several days; this is beneficial for the in vivo tracking of molecular species with longer biological half-life, such as antibodies, nanoparticles, and cells. For the radiolabeling of organic molecules, there are several iodine radionuclides for SPECT imaging, each with a different half-life, allowing short-term (^123^I, *t*_1/2_ = 13.3 h) and long-term imaging studies (^125^I, *t*_1/2_ = 60.5 d; ^131^I, *t*_1/2_ = 8 d). However, ^131^I is also a β^–^ emitter, which underpins it main clinical use as a component of therapeutic radiopharmaceuticals but limits its application for cell tracking. Clinical imaging with ^125^I is limited by its long half-life and the low energy of its emissions (27–35 keV).

**Table 1:** Table Showing the Properties of Various Radionuclides Used for SPECT Imaging

radionuclide	half-life	max. energy (keV)	decay	production	common production reaction
^198^Au	2.7 d	960	β^–^, γ	cyclotron	^197^Au(n,γ)^198^Au
^199^Au	3.1 d	452.6	β^–^, γ	cyclotron	^198^Au(n,γ)^199^Au
^67^Ga	78.3 h	300	Auger e^–^, γ	cyclotron	^68^Zn(p,2n)^67^Ga
^111^In	2.81 d	245	Auger e^–^, γ	cyclotron	^111^Cd(p,n)^111^In
^123^I	13.3 h	159	Auger e^–^, γ	cyclotron	^127^I(p,5n)^123^Xe
^125^I	60.5 d	35	Auger e^–^, γ	nuclear reactor	^124^Xe(n,y)^125^Xe → ^125^I
^131^I	8.0 d	610	β^–^, γ	nuclear reactor	^130^Te(n,γ)^131^Te → ^131^I
^188^Re	16.9 h	155	β^–^, γ	generator	^188^W/^188^Re
^99m^Tc	6.0 h	140	γ	generator	^99^ Mo/^99m^Tc

### 2.2 Positron Emission Tomography (PET)

Positron emission tomography (PET) involves the imaging of positron (β^+^) emitting radionuclides. When the emitted positrons encounter electrons, they undergo mutual annihilation due to the matter-antimatter interaction, resulting in the release of energy in the form of two gamma photons, which are emitted in opposite directions at an approximate 180° angle from each other with a distinct energy of 511 keV (Figure 3B). PET scanners allow the detection of these 511 keV γ rays (known as coincidence detection) by using a ring of gamma detectors. The location of the annihilation event can be determined along a so-called “line of response”, which in turn allows the approximate position of the positron-emitting radionuclide to be elucidated. Positrons are emitted from the nucleus in random directions and can travel a short distance (up to a few mm in tissue, depending on their energy) before annihilating. This distance is known as the positron range and fundamentally limits the spatial resolution of the PET scanner; PET radionuclides with high positron energy will have a long positron range, meaning a greater uncertainty on the position of the emitting nucleus and therefore a poorer spatial resolution.

A selection of PET radionuclides is shown in Table 2. Small molecules are often radiolabeled with “organic” PET radionuclides, such as ^11^C and ^18^F to give radiotracers with unchanged or almost unchanged chemical structures. ^18^F (*t*_1/2_ = 110 min) is currently the most widely used PET radionuclide in the clinic, usually as the glucose derivative [^18^F]fluoro-2-deoxy-d-glucose ([^18^F]FDG, see section 4.4) used mainly for cancer and inflammation imaging. There are also longer-lived organic PET radionuclides, such as ^124^I (*t*_1/2_ = 4.2 d) and ^76^Br (*t*_1/2_ = 16 h). As well as the organic PET radionuclides, several radiometals are available for use with PET (Table 2). Like ^99m^Tc, ^68^Ga (*t*_1/2_ = 67.6 min) offers the benefits of generator production and is widely used preclinically and increasingly in the clinic for labeling peptides and small molecules. The longer-lived ^64^Cu (*t*_1/2_ = 12.7 h) and ^89^Zr (*t*_1/2_ = 3.3 d) are also commonly used for PET imaging of long-circulating antibodies, nanoparticles, and cells.

**Table 2:** Table Showing the Properties of a Selection of Radionuclides Used for PET Imaging

radionuclide	half-life	max. energy (keV)	decay	production	common production reaction
^15^O	2.1 min	1732	β^+^	cyclotron	^15^N(p,n)^15^O
^13^N	9.9 min	1199	β^+^	cyclotron	^16^O(p,α)^13^N
^11^C	20.4 min	961	β^+^	cyclotron	^14^N(p,α)^11^C
^68^Ga	67.6 min	1899	EC, β^+^	generator	^68^Ge/^68^Ga
^18^F	109.7 min	634	EC, β^+^)	cyclotron	^18^F(F^–^): ^18^O(p,n)^18^F
^62^Cu	9.7 min	2926	β^+^	generator	^62^Zn/^62^Cu
^64^Cu	12.7 h	656	EC, β^+^, β^–^	cyclotron	^64^Ni(p,n)^64^Cu
^89^Zr	78.4 h	900	EC, β^+^	cyclotron	^89^Y(p,n)^89^Zr
^124^I	4.2 d	2100	EC, β^+^	cyclotron	^124^Te(p,n)^124^I
^52^Mn	5.6 d	1434	β^+^	cyclotron	^52^Cr(p,n)^52^ Mn

### 2.3 Advantages and Disadvantages of Radionuclide Imaging

Radionuclide-based imaging techniques have several properties that are worth discussing in the context of the previously discussed imaging techniques. First, unlike optical imaging modalities, radionuclide imaging has no major tissue depth penetration limitations, and its large field of FOV means it can usually be performed on a whole-body scale. However, radionuclide imaging has lower spatial resolution compared to MRI and CT. Furthermore, the use of radionuclides means that the radiation doses the subject receives during scanning must be carefully considered and managed, particularly when combined with CT imaging. A large benefit of radionuclide imaging is how sensitive (10^–10^–10^–12^ M—the typical radionuclide concentration in vivo) it is compared to other imaging modalities with a large FOV, such as MRI and CT. This usually means the administered radiotracers (in the scale of micrograms or less, c.f. grams for MRI/CT) do not perturb the biological system being imaged or cause significant toxicity. For example, receptor-targeted radiopharmaceuticals can usually be used without risk of saturating or significantly activating the receptors. Radionuclide imaging is, therefore, well suited for the imaging of molecular processes (known as molecular imaging), while also being highly versatile in that very many processes can be targeted for imaging. Additionally, radioactive emissions do not suffer from significant tissue attenuations, allowing quantification of tissue uptake ex vivo and in vivo with high accuracy and temporal resolution. This can make it highly complementary when used with other modalities (such as MRI and CT), which allow high resolution imaging but suffer from lower sensitivity and do not generally image molecular processes.

### 2.4 PET versus SPECT

As mentioned above, both PET and SPECT have lower spatial resolution than other medical imaging techniques. The spatial resolution of current clinical SPECT scanners (7–15 mm) is lower than PET scanners (6–10 mm).^28^ However, preclinically there is little difference in spatial resolution between PET and SPECT; both are capable of submillimeter resolution.^29^ In SPECT, the use of collimators excludes a large fraction of gamma ray emissions from the radionuclides, while with PET this is not the case making the modality more effective at detecting decay events. SPECT imaging also has the advantage that multiple isotopes and radioactive compounds can be used in the same subject to image different molecular targets simultaneously, due to the distinct energy emissions that SPECT radionuclides may have. This is known as multiplexed imaging.^30^ In contrast, multiplexed imaging is not possible with current PET scanners, as the annihilation γ rays detected by PET imaging have the same 511 keV energy regardless of the positron energy or radionuclide. Additionally, clinical SPECT imaging is generally less costly and more widely available than PET imaging, although the latter is becoming increasingly widely available. Finally, the recent development of a new form of clinical PET, “total-body PET”, offers a step change in the potential versatility and capability of this technique. Total-body PET scanners allow the imaging of radiotracers in humans at significantly lower radiation doses (up to 40×), much shorter acquisition times,^31,32^ or both. The potential impact of this technology on cell tracking will be discussed later.

## 3 Overview of Cell Radiolabeling and Tracking Methods

**Figure 4:**
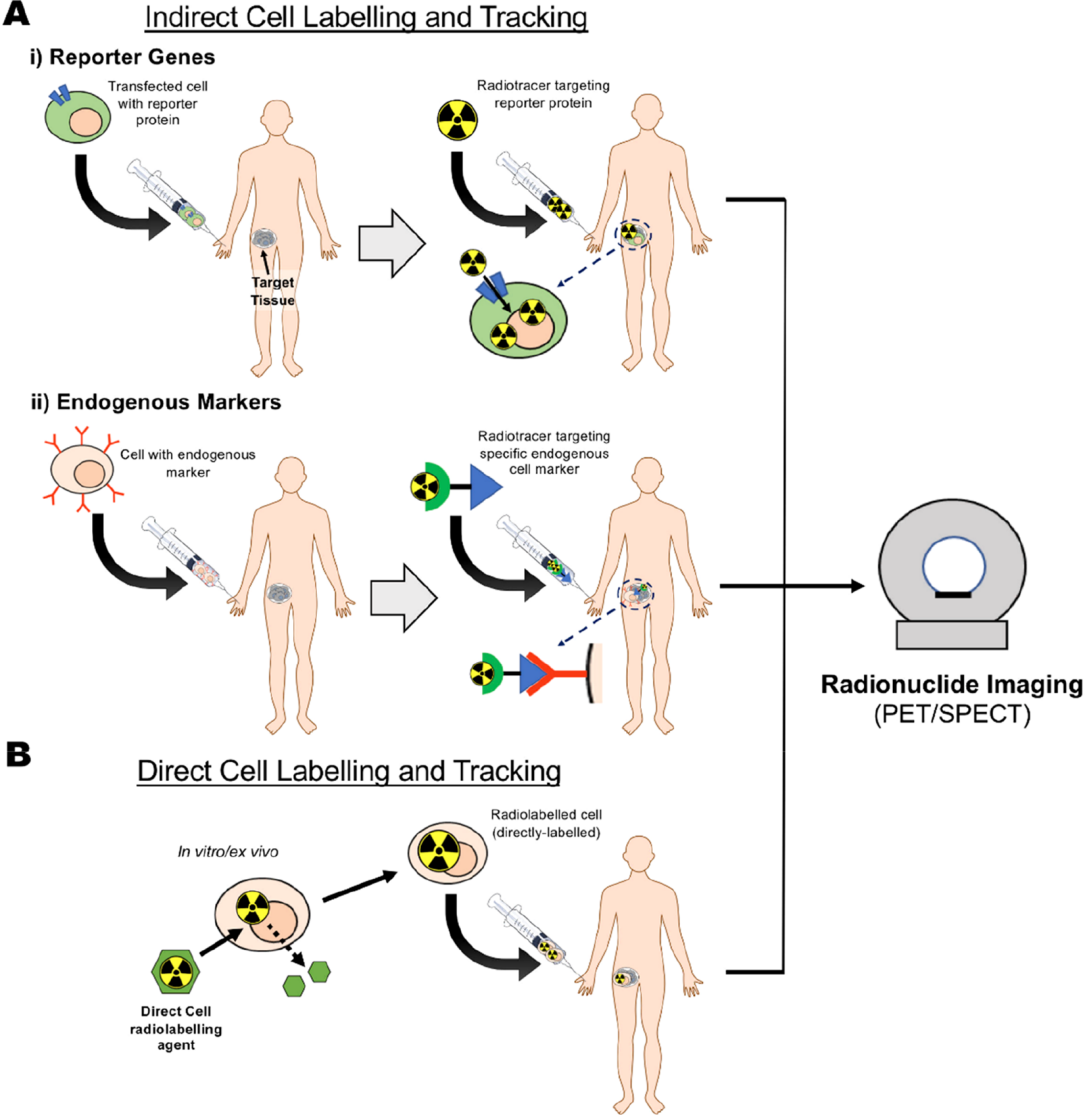
Schematic representation of in vivo cell tracking methods using radionuclides. (A) (i) Indirect cell labeling and tracking; cells transfected with a reported gene are administered into the living subject, followed by a radiotracer targeting the specific reporter gene/protein. This radiotracer can be administered over the lifetime of a subject, allowing longitudinal imaging. (ii) Alternatively, cells expressing an endogenous marker (e.g., T-cell receptor) are administered into the living subject. Target uptake and distribution of the cells can then be imaged in vivo by administration of a radiotracer targeting the specific cell marker (e.g., radiolabeled antibodies). (B) Direct cell labeling and tracking. Cells are radiolabeled in vitro/ex vivo using a direct cell labeling agent. The cells are washed to remove unreacted radiotracer and then administered in the living subject for in vivo imaging using radionuclide imaging.

In the previous section, we have discussed the various benefits of radionuclide imaging for in vivo cell tracking methods compared to other modalities available. We will now briefly discuss the various in vivo tracking methodologies used with radionuclide imaging (Figure 4) with a focus on the benefits and pitfalls of each.

### 3.1 Indirect Cell Labeling and Tracking

As discussed in section 1.2, indirect cell labeling requires the genetic manipulation of cells to express a reporter gene. Within the context of radionuclide imaging, a reporter gene is usually a protein (receptors, transporters and enzymes) that facilitates the uptake or binding of a radiotracer, which after administration of the cells allows “hotspot” imaging of their location within the body by repeat injections of the radiotracer (Figure 4A). For example, receptor-based reporter genes induce the expression of cell receptors that can then be targeted by specific imaging tracers. Several researchers have modified cancer cell lines with the human somatostatin type 2 receptor (hSSTR2), a gene that is not significantly expressed in healthy adult tissues. This allows in vivo imaging of tumors using a ^99m^Tc-labeled peptide conjugate that specifically targets hSSTR2.^33,34^ More recently, the prostate specific membrane antigen (PSMA) was used as a reporter gene for the tracking of CAR T-cells using the prostate cancer PET agent [^18^F]DCFPyL.^35^ Similarly, transporter-based reporter genes, such as the sodium-iodide symporter (NIS), allow the cellular uptake of radiotracers through cell membrane transporters. Cells genetically modified with NIS can be imaged in vivo using iodide-mimicking radiotracers such as [^99m^Tc]TcO_4_^–^, [^18^F]BF_4_^–^, [^18^F]SO_3_F^–^, and [^18^F]PF_6_^–^, as well as radioiodine isotopes ([^123/124/125^I]NaI), using PET and SPECT.^36−41^ Finally, enzyme-based reporter genes allow tracking of cells via the enzymatic trapping of radiotracers within genetically modified cells. A prominent example is the genetic modification of cells to express the herpes simplex virus type 1 thymidine kinase gene (HSV1-tk). Upon entering the modified cells, radiolabeled substrates of HSV1-tk such as 9-[4-[^18^F]fluoro-3-(hydroxymethyl)butyl]guanine ([^18^F]FHBG) are phosphorylated by the enzyme and trapped within the cell.^42^

One major drawback of indirect cell labeling is the need to genetically modify cells, which is often considered to be a significant barrier to clinical translation because of the increased complexity of the technique and the requirement for additional safety evaluation. However, for cellular therapies that inherently involve genetic manipulation (e.g., CAR T-cells), this should not in principle represent a significant issue. Indeed, Gambhir and collaborators have reported the clinical tracking of CAR T-cells using reporter gene technology with PET.^42,43^ Alternatively, indirect cell tracking can be performed using radiotracers targeted to specific endogenous cell markers present on the cells of interest (Figure 4Aii) even without genetic manipulation.^44^ A key recent example of this was reported by Simonetta et al., who used immunoPET to image the Inducible T-cell COStimulator (ICOS) which was up-regulated during activation of human CD19.28z CAR T cells.^45^ Anti-ICOS mAbs radiolabeled with ^89^Zr enabled the in vivo imaging of activated CAR T-cells without damaging the antitumor effect of the therapeutic cells. However, the use of radiolabeled antibodies may be undesirable due to their long blood half-lives. To overcome this, smaller binding proteins with shorter circulation half-lives and faster clearance such as radiolabeled peptides,^46^ single-chain Fv fragments (scFv)^47,48^ and minibodies^49^ targeting cell markers have been used. One potential limitation with this approach is the limited number of radiotracer molecules per cell. While imaging surface markers allows for a more specific approach, the 1:1 ratio of targeting ligand to surface protein may limit the sensitivity of the method when low numbers of infiltrating cells are present.^44^ Direct labeling and, to some extent, indirect cell labeling using reporter genes, overcome this issue by allowing many more radiotracer molecules per cell. Additionally, the use of an exogenously administered imaging tracer has the drawback of leading to misinterpretation of the imaging signal, as hotspots associated with the tracer cannot be distinguished from those associated with the target cells. For example, the signal of imaging tracers cleared through the liver may be misinterpreted as the presence of administered cells. Furthermore, this method is limited to specific examples where the cell of interest has unique or low abundant targetable proteins. While indirect cell labeling is not the focus of this review, it remains a highly valuable cell tracking tool and readers are referred to other reviews on this topic.^21,50^

### 3.2 Ex Vivo Direct Cell Labeling

Compared to indirect cell labeling, direct cell labeling is a simpler cell tracking method that does not involve the genetic manipulation of cells. Cells are usually radiolabeled ex vivo/in vitro by incubation with a radiotracer, followed by injection of the radiolabeled cells into the imaging subject (Figure 4B). In vivo PET or SPECT imaging can then be performed over time to assess the distribution of the cells. The radiolabeling mechanism can vary depending on the type of probe. Cells can be radiolabeled using radiotracers designed to bind to or integrate into the cell membrane. Alternatively, imaging probes can be specifically designed to permeate the cell membrane and become trapped intracellularly. Finally, cells can be labeled via the uptake of radiolabeled particles, which can be mediated by endocytic or phagocytic pathways. A limitation of direct cell labeling is that the imaging time window of this technique is limited by the half-life of the radionuclide used. Direct cell labeling can also be restricted by the efflux of the radiotracer/radionuclide from the radiolabeled cells in vivo. Additionally, information on in vivo cell proliferation cannot be determined because when cells divide, the radionuclide probe will be redistributed between daughter cells, causing “label dilution”.^1^ Hence, ideal direct cell labeling agents should facilitate fast, efficient (high yield) cellular uptake, with high cellular retention of the radionuclide (slow label efflux), while not affecting the cell viability. Furthermore, they should allow imaging over relatively long periods of time (if needed for the imaging application). Hence, long-lived radionuclides (such as ^111^In, ^89^Zr) are usually preferred.

## 4 Chemical Probes for Ex Vivo Direct Cell Radiolabeling

As outlined in previous sections, attaching a radiolabel to cells prior to their administration—ex vivo direct cell radiolabeling—is the most straightforward and robust method of radiolabeling and tracking cells with PET/SPECT. The simplicity of direct cell labeling ex vivo means that in theory any chemical probe capable of entering or binding to cells can be repurposed for this application, and various cellular chemistries and processes can be utilized for this purpose. In practice, several concepts should be carefully considered before selecting a cell labeling agent. In this section, we will review the various methodologies used for direct cell tracking and discuss the broad library of chemical probes that have been developed for each method, and their respective benefits and disadvantages. First, we will introduce and define basic cell radiolabeling concepts, which will be referred to throughout the rest of the Review.

### 4.1 Key Concepts for Direct Cell Radiolabeling

#### 4.1.1 Cellular Uptake/Labeling Efficiency

**Figure 5:**
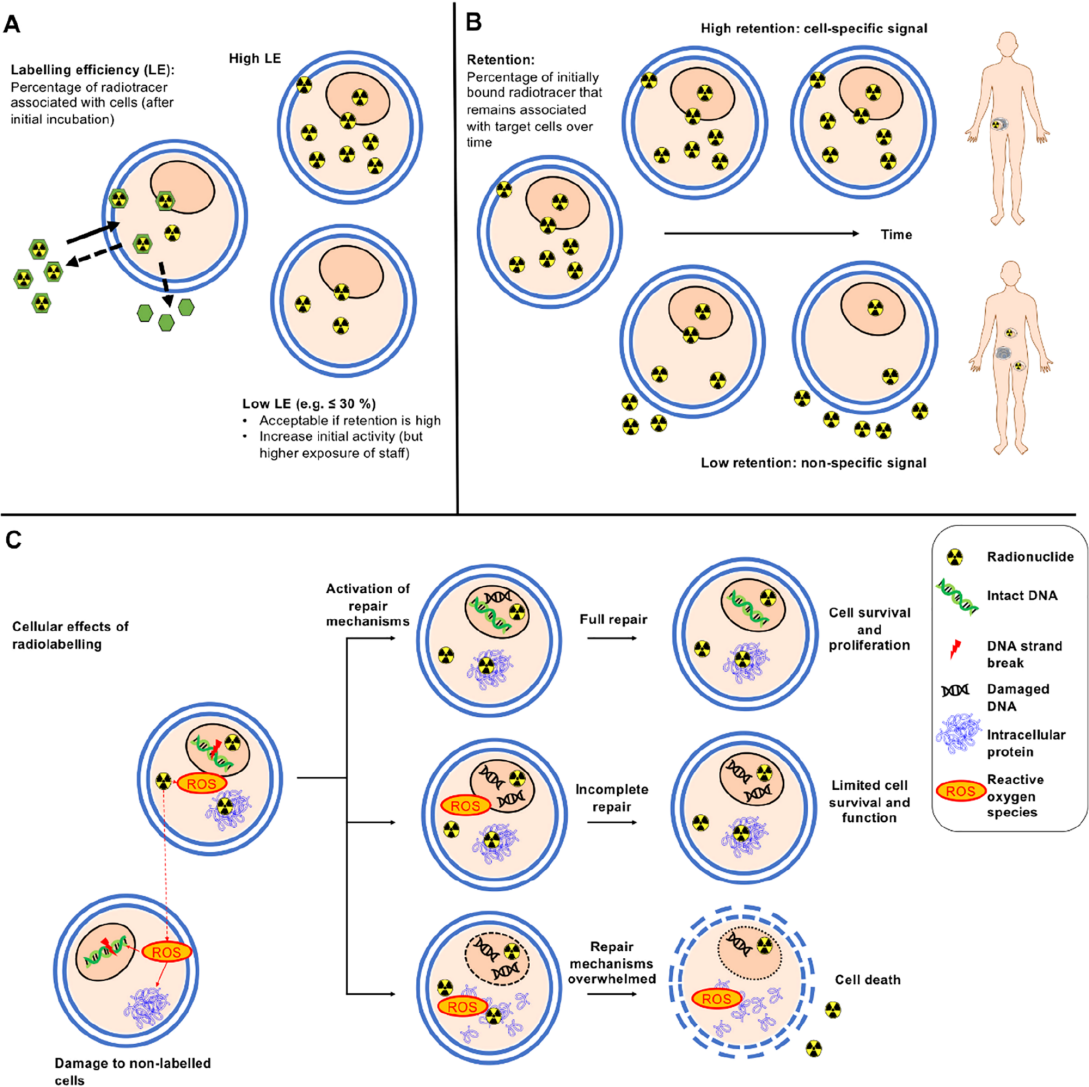
Key concepts in cell labeling. (A) Labeling efficiency (LE) depends on the radiotracer, cell type, and labeling conditions. A high labeling efficiency is preferable, however lower labeling efficiencies are acceptable if the subsequent retention of radioactivity by the cells is sufficiently high for the desired imaging period. To compensate for low LE, labeling can be performed with a higher starting activity to achieve the desired activity in the subject to be imaged. However, higher starting activities may pose additional costs and risks to staff involved in radiolabeling. (B) Retention of activity by labeled cells. High retention of activity within the labeled cells over the desired imaging period is essential to obtain meaningful images, even if labeling efficiencies are lower. Low retention of radioactivity by labeled cells can lead to less specific images as the localization of the radionuclide becomes decoupled from that of the cells of interest. (C) Cellular effects of radiolabeling. Radionuclides can damage cellular components directly (e.g., DNA strand breaks caused by Auger electrons or positrons) and indirectly (via water radiolysis and ROS generation). In response to ionizing radiation, cells activation endogenous repair mechanisms. Depending on the extent and nature of the damage, these repair mechanisms can salvage cells, partially repair the cells leaving them incompletely functional, or they can be overwhelmed, leading to rapid cell death. Depending on the nature of the radiation, neighboring nonlabeled cells can also be affected.

A key concept for assessing a direct cell labeling agent is the extent of cellular uptake, which refers to the amount (%) of radioactivity associated with cells. This is often expressed as labeling efficiency (LE; Figure 5A), defined as the percentage of radioactivity added that is associated with the cells after the labeling process. Generally, after the incubation of a direct cell radiolabeling agent with the target cells, the reaction is “quenched” by removal of the supernatant. If the cells are in suspension, this is usually done by pelleting the cells (i.e., gentle centrifugation) and removing the supernatant, followed by a washing step. Typically, LE is defined by the equation below:
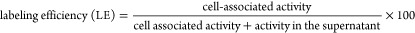
However, there are other ways of expressing cellular uptake, which provide additional information, such as activity/cell, percent activity added per milligram of protein or a ratio of intracellular/extracellular radioisotope concentration.^51^ These units have the benefit of correcting for cell numbers, which may affect cellular uptake; higher cell numbers are expected to lead to higher labeling efficiencies. Hence, the method used to calculate and compare cellular uptake of radiotracers should be carefully considered for each radiotracer, both when designing studies or interpreting results from the literature. High labeling efficiencies are desirable to reduce waste of expensive radionuclide and minimize problems associated with purification steps, particularly when cell numbers are restricted.

#### 4.1.2 Cellular Retention of the Radiolabel

A second fundamental aspect of direct cell radiolabeling is the retention of the radiotracer/radionuclide inside or on the surface of the cells after quenching of the radiolabeling step. This is of high importance because, unlike fluorescence or bioluminescence, radioactive emissions cannot be “switched off” or selectively activated and all radiotracer signal will be acquired by the detector whether it originates within the labeled cell or not. Consequently, it is difficult to tell a priori from a PET or SPECT image whether the signal represents live cells, damaged cells, radioactive cell debris, or leaked radiotracer (Figure 5B). To mitigate this, several approaches should be taken in conjunction. First, the radionuclide retention should be maximized, ideally for the useful duration of the study. This includes considering the physicochemical interactions of the radiotracer with the various cellular constituents (e.g., receptors, membrane, intracellular proteins) and its intracellular metabolism, but also ensuring that the amount of radiotracer does not result in significant cell damage. Second, any unincorporated radiotracer should be removed by washing the cells after incubation with the radiotracer and before further use in vitro or in vivo, to ensure that at least at the point of administration the radioactivity is fully associated with the cells of interest. Calculation of radiotracer retention is performed using the same equation as for LE, the only difference being that it is measured at a specified time after the initial radiotracer incubation and washing step. The factors that can affect radiolabel retention will be discussed in more detail in section 5.2.

#### 4.1.3 Cell Viability and Functionality

Finally, it is essential that direct cell labeling methods have no significant effect on the viability, activity, motility, and trafficking of the target cells, because the radioactive signals from directly labeled cells do not report on whether the cells are alive or functioning normally. This is important because dying (e.g., apoptotic) or dead cells not only have different circulating patterns from live cells in the body but can also release their radiolabel more quickly. This may lead to misleading images. It is therefore essential to assess the damage the radiolabeling method may do to the target cells over time. Ideally this should be performed over a period of time corresponding to the desired in vivo imaging time frame. As well as the viability of radiolabeled cells, the functionality of these cells must not be affected by the radiolabeling method. For example, cytotoxic cells (i.e., CAR T-cells) should be tested to confirm they retain their cell-targeting and killing ability after radiolabeling. The viability and functionality of cells can be affected by the radiotracer itself (e.g., through radiation-induced DNA damage; Figure 5C), as well as the labeling conditions along with the chemical compounds used to mediate radiolabeling. Hence, it can be important to perform suitable controls (i.e., with the absence of radioactivity) to establish the potential cause for any effects on cell viability or functionality observed. A more detailed discussion on the effects of radionuclides on cell viability and testing the functionality of radiolabeled cells can be found in sections 5.3 and 5.4, respectively.

**Figure 6:**
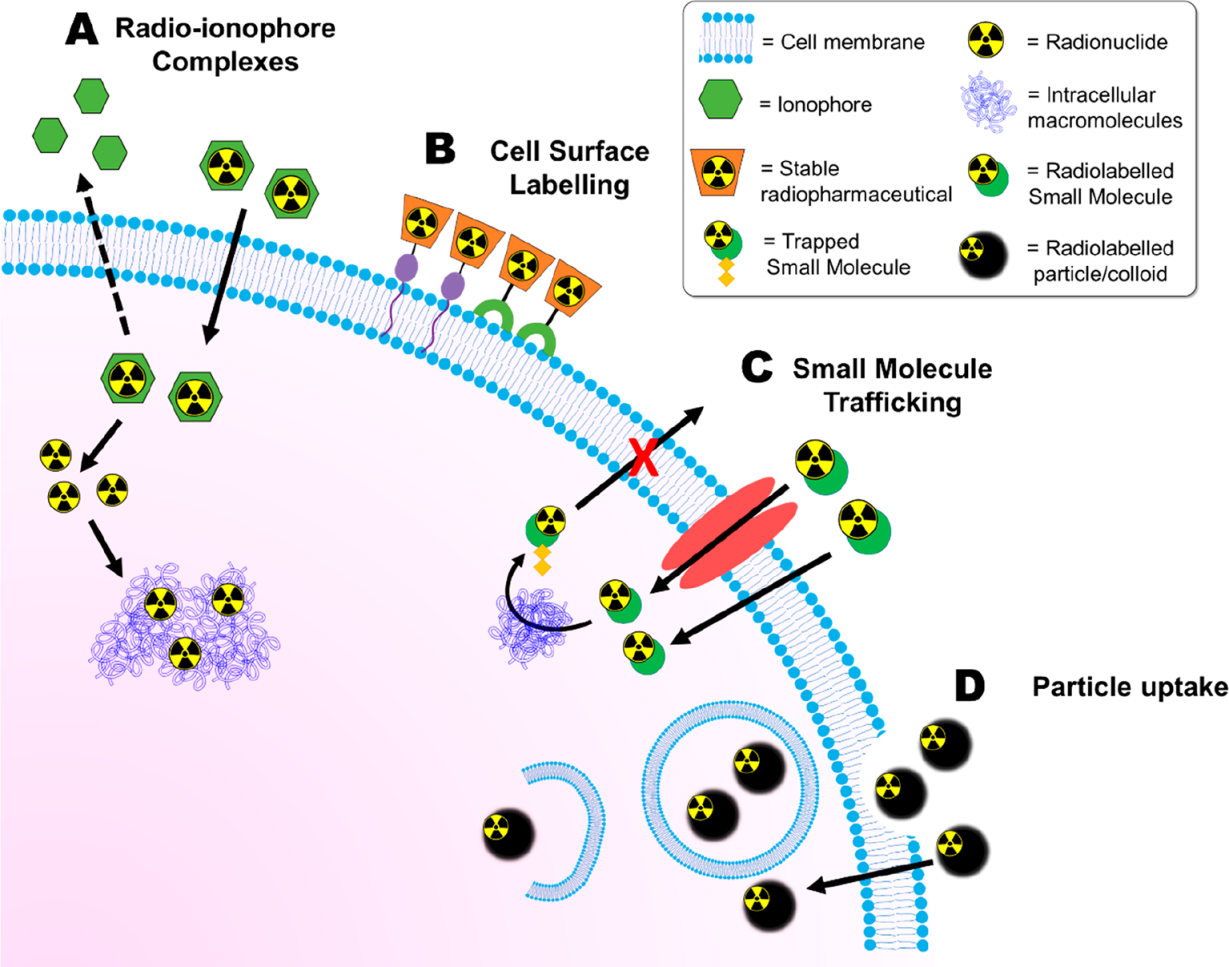
Schematic overview of the main methods for direct cell radiolabeling. (A) Radio-ionophore complexes. The ionophore ligand forms a complex with a radionuclide which allows it to cross cell membranes. Once inside the cell, the radioisotope is released and trapped by binding to intracellular macromolecules. (B) Surface of cells can be radiolabeled using stable radiopharmaceuticals which can bind covalently to components of the cell surface (e.g., proteins) or via compounds which can interact with the lipid membrane. (C) Radiolabeled small molecules can be used for direct cell labeling. They can enter cells through passive or active transport mechanisms and subsequently be converted into hydrophilic forms which are unable to diffuse out of cells. (D) Radiolabeled particles, such as colloids and nanoparticles, can be taken up by cells through phagocytic processes.

We will now discuss in detail different chemical methods that have been developed for the radiolabeling of cells in vitro/ex vivo, summarized in Figure 6.

### 4.2 Radiometal–Ionophore Complexes

Most compounds used for direct cell radiolabeling are “radiometal–ionophore” complexes, which consist of a radiometal and an ionophore. An ionophore is defined as a ligand which binds to a metal ion reversibly for transport across lipid membranes.^52^ The resulting radiometal complex is sufficiently hydrophobic to allow passage across cell membranes but insufficiently stable to remain intact within the cell (Figure 6A). Once inside the cell, the radiometal can be transchelated by intracellular proteins/macromolecules,^53^ resulting in trapping of the radionuclide–and a radiolabeled cell. Effective radio-ionophore agents should facilitate fast uptake and slow radionuclide efflux (which requires rapid transchelation once inside the cell), while not affecting the cell viability. Table 3 lists the various ionophore ligands used for direct cell radiolabeling.

**Table 3:** Table Summarizing the Various Ionophore Ligands Used for Direct Cell Labelling, along with Their Corresponding Radionuclides and the Cell Type Labeled

ionophore ligand	radionuclide	cell type labeled	ref
Oxine (8-hydroxyquinoline)	^99m^Tc	RBCs; WBCs	(54)
		platelets	(55)
	^111^In	RBCs; WBCs	(54)
		platelets	(55)
		neutrophils	(53)
		T-cells	(56)
		hepatocytes	(57)
		dendritic cells	(58, 59)
		human endothelial progenitor cells	(60)
		mesenchymal stem cells	(61−63)
		cytolytic T lymphocytes	(64)
		hematopoietic progenitor cells	(65)
		monocytes	(66)
		gamma-delta T cells	(67)
	^68^Ga	platelets	(68)
		RBCs	(69, 70)
		CAR T-cells	(71)
	^89^Zr	breast cancer cells (MDA-MB 231); mouse macrophage (J447)	(72)
		leukocytes	(3, 73, 74)
		mouse myeloma cells (5T33)	(3)
		CAR T-cells	(71, 75)
		cytotoxic T-cells; dendritic cells	(76)
		bone marrow cells	(76−79)
		natural killer cells	(77, 80)
		gamma-delta T cells	(81)
		T-cells	(82)
		Jurkat cells	(83)
		RBCs	(84)
		mesenchymal stem cells	(85)
		endothelial progenitor cells	(86)
	^64^Cu	RBCs; WBCs	(84)
	^52^Mn	gamma-delta T cells; breast cancer cells (MDA-MB 231)	(87)
tropolone	^111^In	platelets	(88, 89)
		leukocytes	(90)
		neutrophils	(91)
		mesenchymal stem cells	(92−94)
		gamma-delta T cells	(95)
		CAR T-cells	(96)
	^68^Ga	RBCs	(84)
		platelets	(68)
	^64^Cu	leukocytes	(97)
		RBCs; WBCs	(84)
	^89^Zr	RBCs	(84)
		mouse macrophage cell line (J447)	(72)
2-mercaptopyridine-*N*-oxide (MPO)	^111^In	platelets	(98)
		leukocytes	(99, 100)
	^68^Ga	platelets	(68, 101)
	^67^Ga	platelets	(102)
hydroxypyranones	^111^In	leukocytes	(103, 104)
ethyl maltol	^89^Zr	colon cancer cells (HTC-116)	(72)
acetylacetone	^111^In	RBCs	(105, 106)
		leukocytes	(54)
dithiocarbamates	^99m^Tc	leukocytes	(107)
	^64^Cu	J774 mouse macrophages	(108)
*N*-ethoxy-*N*-ethyl-dithiocarbamate (NOET)	^99m^Tc	leukocytes	(109)
	^188^Re		
dithiocarboxylates	^99m^Tc		(110)
HMPAO	^99m^Tc	leukocytes	(111)
		dendritic cells	(112)
		T-cells	(113)
bis(thiosemicarbazones)	^64^Cu	glioma cells (G6)	(114)
		rhesus monkey mesenchymal stem cells	(115)
		glioblastoma cells (U87MG)	(116)
		OVA-Th1 cells	(117)
		J774 mouse macrophages	(108)
poly(ethylenimine)	^64^Cu	glioblastoma cells (U87MG)	(116)

#### 4.2.1 8-Hydroxyquinoline (Oxine)

**Figure 7:**
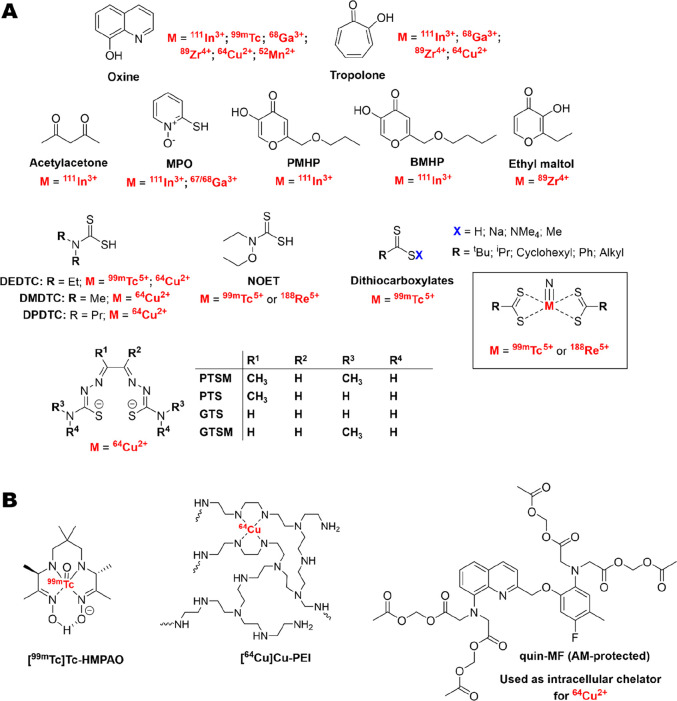
(A) Chemical structures of all ionophore ligands discussed in this Review along with the corresponding radionuclides used for cell labeling. (B) Chemical structures of key radiometal–ionophore complexes and chemical compounds used for radiometal–ionophore cell radiolabeling. Note that while [^99m^Tc]Tc-HMPAO has been categorized as a radiometal–ionophore complex, the exact cellular trapping mechanism is not known.

**Figure 8:**
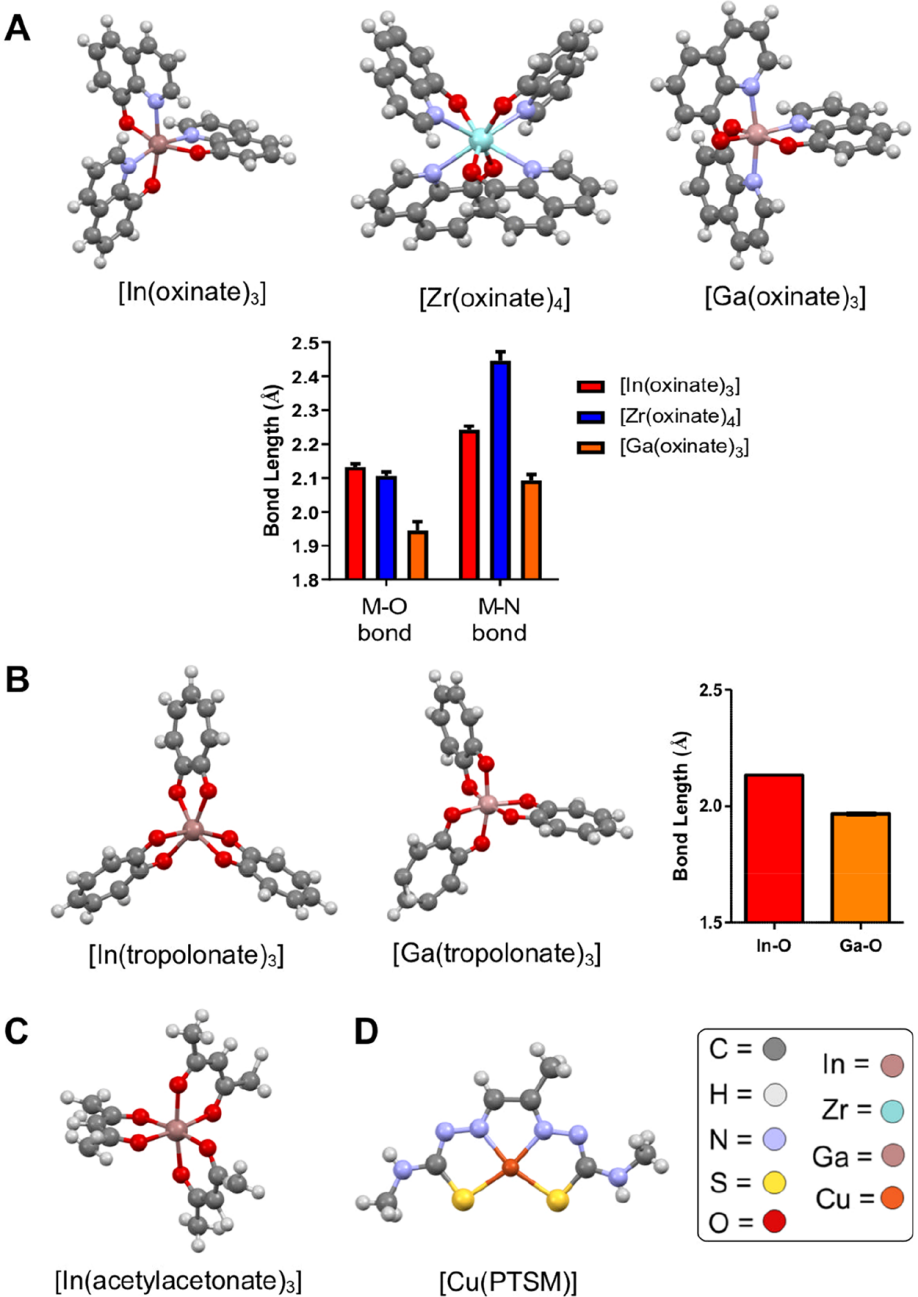
X-ray crystal structures of various metal-ionophore complexes discussed in this Review. (A) Structures of the oxine complexes of In^3+^, Zr^4+^, and Ga^3+^ (structures from refs (127 and 128)) and the corresponding metal–ligand bond lengths of each complex (M = metal). (B) Structures of the tropolone complexes of In^3+^ and Ga^3+^,^129^ and the corresponding metal–ligand bond lengths of each complex. Structures of (C) In(acetylacetonate)_3_^130^ and (D) Cu-PTSM.^131^ X-ray structure visualization and data analysis was performed using Mercury CSD.^132^

8-Hydroxyquinoline (oxine, Figure 7A) is a metal-chelating ligand known to bind a wide variety of metals through the pyridyl nitrogen and the hydroxyl group, which becomes deprotonated, allowing the formation of neutral, lipophilic metal complexes.^118,119^ To the best of our knowledge the first use of oxine for direct cell labeling with radionuclides was in 1976 by McAfee et al., who reported the synthesis of the [^99m^Tc]Tc-oxine and [^111^In]In-oxine complexes for the labeling of red blood cells (RBCs) and white blood cells (WBCs/leukocytes).^54^ Following these initial uses, both compounds were subsequently used for the radiolabeling of platelets.^55^ The indium metal center in [^111^In]In-oxine is likely in the 3+ oxidation state, and the observed lipophilicity of the compound suggests that the most likely chemical identity is the neutral [^111^In]In(oxinate)_3_ complex (X-ray structure with nonradioactive ^113^In isotope in Figure 8A). However, because of the complex redox chemistry of technetium, the identity of the [^99m^Tc]Tc-oxine complex is not known. Technetium(V) complexes of oxine have been previously reported in the oxo [^99^Tc][TcO(oxinate_2_)]^+^ form.^120^ However, these complexes were synthesized from different precursors (tetrabutylammonium tetrachlorooxotechnetate(V)) compared to the [^99^Tc]Tc-oxine preparation ([^99m^Tc]TcO_4_^–^ with tin pyrophosphate);^121^ therefore, this may not be the structure of the radioactive complex. Regardless, only [^111^In]In-oxine was taken further and was later used to image leukocytes in humans,^122^ eventually being approved for leukocyte imaging by the FDA in 1985 and used clinically for imaging inflammatory disease. [^111^In]In-oxine labeling of cells required a medium free of plasma proteins because of transchelation of the ^111^In. This was a particular issue when labeling platelets due to in vitro damage to the cells.^123^ Additionally, oxine has low solubility in aqueous solvents, and early protocols consequently entailed a variety of organic solvents (i.e., ethanol, chloroform) for synthesis and purification–which can be cytotoxic.^123,124^ Furthermore, the [^111^In]In-oxine complex is highly lipophilic, causing reduced recovery in aqueous medium due to adherence to plastic/glass vessels. These problems were overcome later by the use of the surfactant polysorbate in formulations.^73,125^ The [^111^In]In-oxine formulation was withdrawn from the EU market by GE Healthcare, apparently because of insufficient medical demand,^126^ although it is now available in Europe from Curium. It was replaced by [^99m^Tc]Tc-HMPAO (see section 4.2.4) for the labeling and tracking of leukocytes—the primary use of the tracer in clinics at that time. However, the need for tracking cells for longer periods of time has recently resulted in a renewed interest in [^111^In]In-oxine for the in vivo tracking of cellular therapies preclinically and clinically.

The use of oxine as an ionophore for ^68^Ga was first reported by Welch et al.; being the first use of a PET radiometal for cell labeling.^70^ Because of the redox inertness of Ga^3+^, the neutral [^68^Ga]Ga-oxine complex is likely the [^68^Ga]Ga(oxinate)_3_ complex (X-ray crystal structure in Figure 8A). The [^68^Ga]Ga-oxine complex was used to radiolabel both red blood cells and platelets with ∼93% LE for the former,^70^ and lower for platelets (∼20–50*%* after washing).^68^ This is possibly due to presence of transferrin in the platelet labeling mixture, which may transchelate the ^68^Ga^3+^ ion. More recently, [^68^Ga]Ga-oxine was used for the radiolabeling of CAR T-cells with high cellular retention (>90% after 2 h), with no effect on cell viability up to 48 h.^71^ However, [^68^Ga]Ga-oxine has limited use for cell tracking applications that require long imaging timeframes because of the short half-life of ^68^Ga (68 min). Nevertheless, [^68^Ga]Ga-oxine was recently used clinically for the labeling and tracking of heat-denatured RBCs over short periods with clinical PET/CT imaging.^69^

**Figure 9:**
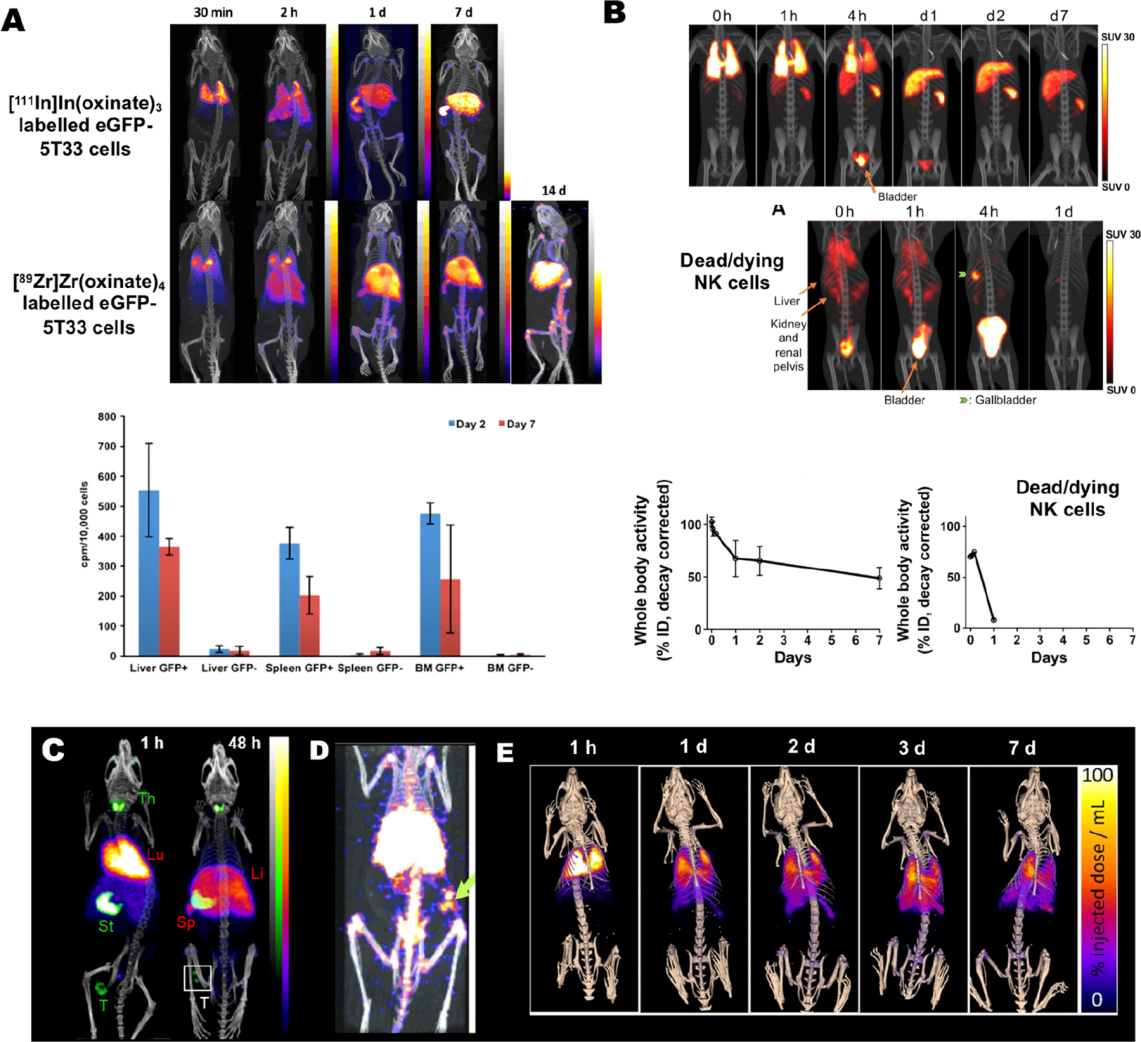
(A) PET/CT and SPECT/CT images of C57Bl/KaLwRij mice inoculated with [^89^Zr]Zr(oxinate)_4_ (bottom row) or [^111^In]In(oxinate)_3_ labeled (top row) eGFP-5T33 cells from 30 min to 14 days after i.v. inoculation. Bottom figure shows the ^89^Zr activities in eGFP-positive and eGFP-negative cell populations sorted from liver, spleen, and femoral marrow (BM) organ homogenates harvested from mice 2 and 7 days after i.v. inoculation with [^89^Zr]Zr(oxinate)_4_-labeled eGFP-5T33 cells; showing that the radioactivity in the target tissues remained associated with the originally labeled eGFP-expressing cells and hence that these cells remained alive over 7 days in vivo. Adapted with permission from Charoenphun et al., ref (3). Copyright 2015 Springer Nature under CC License [https://creativecommons.org/licenses/by/4.0/]. (B) PET/CT imaging of autologous ^89^Zr-labeled expanded NK cells transferred to rhesus macaques, with continuous deferoxamine infusion, for up to 7 days (top row). PET/CT imaging of ^89^Zr-labeled apoptotic NK cells were tracked in a rhesus macaque model under continuous deferoxamine infusion (bottom row). Whole-body activity (%ID) of ^89^Zr-labeled expanded NK cells (bottom left graph) and ^89^Zr-labeled apoptotic NK cells (bottom right graph) showing that DFO is able to clear released ^89^Zr from dead/dying cells. Adapted with permission from Sato et al., ref (134) (Copyright 2020 AACR). (C) Representative PET, SPECT, and CT (merged) scans of a PLA-treated SCID/beige mouse bearing MDA-MB-231.hNIS-GFP xenografts at 1 and 48 h postinjection of ^89^Zr-labeled γδ-T cells. Adapted with permission from Man et al., ref (81). Copyright 2019 Man et al. under CC License [https://creativecommons.org/licenses/by/4.0/]. D) PET/CT images of [^89^Zr]Zr-oxine radiolabeled PSCA CAR T-cells at 162 h postinjection in NSG mice with PC3-PSCA tumors in right flank (arrow). Adapted with permission from Weist et al., ref (75). Copyright 2018 SNMMI. (E) PET-CT images of intravenously injected [^89^Zr]Zr-oxine-labeled uct-MSCs tracked over 7 days. Adapted with permission from Patrick et al., ref (85). Copyright 2020 Springer Nature under CC License [https://creativecommons.org/licenses/by/4.0/].

Similarities between the reactivity and preferred ligand types of In^3+^ and Zr^4+^ have led to the development of a PET alternative to [^111^In]In-oxine for long-term cell tracking using ^89^Zr.^3,72,76^ The neutral [^89^Zr]Zr(oxinate)_4_ ([^89^Zr]Zr-oxine) compound likely exists as the dodecahedral complex (X-ray structure in Figure 8A) based on X-ray crystal structures of the nonradioactive complex.^133^ A comparison of [^89^Zr]Zr-oxine with [^111^In]In-oxine revealed lower or similar cell uptake for [^89^Zr]Zr-oxine, depending on the cell type, but also a lower efflux of ^89^Zr after 24 h.^3^ An in vivo comparison of the two compounds using eGFP-5T33 myeloma cells revealed a significantly higher uptake and retention of ^89^Zr in the target organs (liver, spleen, and bone marrow) compared to ^111^In, with the presence of ^89^Zr-labeled cells confirmed in those organs using FACS analysis (Figure 9A). Sato et al. explored the in vivo retention of ^89^Zr in radiolabeled NK cells in rhesus macaques. They continuously infused the ^89^Zr chelator deferoxamine (DFO) to clear any released activity through the renal system. It was found that the whole-body activity dropped to ∼70% injected dose (% ID) after 1 d, and down to 50% ID after 7 d (Figure 9B). However, after administration of ^89^Zr-labeled dead/dying cells DFO-enhanced renal clearance of ^89^Zr was observed, with the whole-body radioactivity decreased to 8% within just 1 day (Figure 9B).^134^ While this suggests that most of the activity released is from dead/dying cells, the release of the ^89^Zr radiolabel from intact cells due to instability cannot be ruled out. Despite this, the increased retention in vivo of ^89^Zr coupled with the improved imaging properties of PET may allow [^89^Zr]Zr-oxine to extend the useful time frame for tracking cells in vivo. Indeed, PET imaging has been performed preclinically up to 14 days postadministration of cells.^3^ [^89^Zr]Zr-oxine has since been used by several groups for the in vivo tracking of various cell types, particularly for cell therapy models (Table 3;^75,76,81,85,135^Figure 9B–E) and an easy-to-use kit formulation for the clinical radiosynthesis of [^89^Zr]Zr-oxine has also been reported.^73^

The synthesis of [^64^Cu]Cu-oxine has also been reported by Socan et al., who used the compound to radiolabel WBCs and RBCs; the radiometal complex was synthesized using an on-cartridge method with which the corresponding ^68^Ga, ^111^In, and ^89^Zr oxine complexes were also prepared.^84^ [^64^Cu]Cu-oxine showed promising radiolabeling properties with a LE of 67.6% and 57.1% for RBCs and WBCs respectively, and 83% cellular retention of ^64^Cu in RBCs and 55% in WBCs after 48 h. Finally, oxine was reported as an ionophore for ^52^Mn (*t*_1/2_ = 5.6 days); the authors showed that under dilute conditions (to mimic the case in the radiochemistry reaction) the bis(oxine) complex was likely formed with the manganese metal in the 2+ state.^87^ This [^52^Mn]Mn(oxinate)_2_ complex allowed the direct labeling of a variety of cells, and showed comparable labeling of gamma-delta T-cells to [^89^Zr]Zr-oxine. However, cellular efflux of ^52^Mn was rapid, with only 27% remaining in cells after 24 h compared to 78% for ^89^Zr. The released activity was shown to be highly hydrophilic (with a negative LogP value); hence not the [^52^Mn]Mn(oxinate)_2_ complex. Because of the bioactivity of manganese, it is likely the ^52^Mn is trafficked out via a cellular process, possibly through the known manganese efflux pathways, ferroportin^136^ and SLC30A10,^137^ which potentially limits the utility of the agent for direct cell tracking.

#### 4.2.2 Tropolone

2-Hydroxy-2,4,6-cycloheptatrien-1-one (tropolone; Figure 7A) is a bidentate ligand that coordinates metal ions via the two oxygen donor atoms of the carbonyl and hydroxyl group. It was first investigated as an ionophore for cell labeling with ^111^In,^88,89^ likely as the [^111^In]In(tropolonate)_3_ complex (X-ray structure in Figure 8B). The [^111^In]In-tropolone complex was developed as a water-soluble direct cell labeling agent, overcoming the insolubility of oxine in aqueous medium. The higher stability of the tropolone complex also avoids trans-chelation of the radiometal to transferrin, which limited the use of [^111^In]In-oxine for labeling platelets in plasma.^138^ A clinical study showed that [^111^In]In-tropolone-labeled leukocytes could localize lesions with an accuracy similar to those labeled using [^111^In]In-oxine.^139^ However, [^111^In]In-tropolone failed to replace it, likely due to it not being commercially available (at the time), and because it was not demonstrably better than oxine in the clinical setting.^138^

Tropolone was also reported as an ionophore for cell labeling with ^64^Cu.^97^ The [^64^Cu]Cu-tropolone complex was shown to label leukocytes with 83% LE, however the cellular retention was low with just 24% remaining after 24 h. To overcome this, the authors employed a unique approach using an additional chelating agent during the radiolabeling procedure; the membrane-permeable, calcium chelator quin-MF/AM (Figure 7B). This agent crosses the leukocyte cell membrane in its more lipophilic, protected acetoxymethyl (AM) ester form, which cannot bind Cu. However, once inside the cell the AM groups are cleaved by intracellular esterases forming the negatively charged anionic form which has a very high affinity for Cu^2+^. This hydrolyzed form of the compound was proposed to rapidly chelate the ^64^Cu from the tropolone complex, trapping it within the cell. Indeed, radiolabeling with quin-MF/AM present increased the cellular retention at 24 h from 24% to 79%.^97^ Ferris et al. tested tropolone for cell labeling with ^89^Zr. Cell labeling with [^89^Zr]Zr(tropolonate)_4_ was tested in a mouse macrophage cell line (J447) and was found to give ∼22% LE after 1 h, with ∼49% being retained after 24 h (c.f., ∼22% cell uptake obtained with [^89^Zr]Zr(oxinate)_4_ and 91% cellular retention after 24 h.

The tropolone complexes of ^68^Ga (X-ray structure of nonradioactive complex in Figure 8B), ^89^Zr, and ^64^Cu, were also prepared by Socan et al. and their RBC radiolabeling properties compared with those of the corresponding ^68^Ga-, ^64^Cu-, and [^89^Zr]Zr-oxine complexes.^84^ For ^68^Ga, oxine was shown to be more favorable for RBC labeling than tropolone (73% LE and 51% LE respectively). The cellular retention of ^68^Ga was also very low when using tropolone (15% after 4 h) compared with 62% after 4 h for [^68^Ga]Ga-oxine. Oxine was also shown to be a better ionophore for radiolabeling RBCs with ^89^Zr, with 82% and 44% LE for [^89^Zr]Zr-oxine and [^89^Zr]Zr-tropolone, respectively. Furthermore, the amount of ^89^Zr retained in RBCs after 24 h was lower when using tropolone (30%) than with oxine (80%). However, both oxine and tropolone were shown to be favorable for ^64^Cu-RBC labeling, with 70% and 91% LE, respectively. High cellular retention of ^64^Cu was also seen for both compounds with 77% and 86% after 24 h for tropolone and oxine, respectively.^84^ It is possible that the variations in cell uptake and retention observed using various radiometals with tropolone could be related to the differences in Lewis acidity of the metal ions. The “harder” Lewis acids Zr^4+^ and Ga^3+^ may form more stable complexes with the oxygen donors of tropolone compared with the softer Cu^2+^, potentially resulting in lower release of the metals intracellularly—as well as passive diffusion of the stable [^68^Ga]Ga-tropolone and [^89^Zr]Zr-tropolone complexes out of cells. Regardless, this highlights the importance of considering the inorganic coordination chemistry of the radiometal ion used when designing and using ionophores.

#### 4.2.3 Other Ionophore Ligands

Another early reported ionophore for cell labeling was acetylacetone (acac, Figure 7A), which was primarily used for ^111^In—likely as the tris(acetylacetonate) complex. In(acetylacetonate)_3_ is a tris(β-ketoenolato) distorted octahedral complex with the three ligands each forming a six-membered chelate ring with the indium ion (X-ray structure in Figure 8C).^130,140^ The first use of the ligand for direct cell labeling with ^111^In was by Sinn et al. in 1974 for erythrocyte labeling.^105,106^ It was later included in the cell labeling ligand survey by McAfee et al., who reported the radiolabeling of leukocytes.^54^ Initially, as with tropolone, it was developed as an alternative to oxine because of the higher solubility of acetylacetone in aqueous buffers.^106,124^ However, acetylacetone failed to replace oxine and other ionophores for ^111^In, possibly because of less favorable performance in clinical studies. For example, granulocytes labeled with [^111^In]In-acetylacetonate were shown to have inferior sensitivity and visualization of infection in patients, compared cells labeled with [^111^In]In-tropolone.^141^

Another ionophore used for cell labeling is 2-mercaptopyridine-*N*-oxide (MPO, Figure 7A), which is the conjugate base of pyrithione. The ligand is bidentate with metal binding occurring through the negatively charged thiolate and the N-oxide oxygen atom. The [^111^In]In-MPO complex for cell radiolabeling was first developed in 1985 for platelet labeling.^98^ The cell labeling of platelets with ^111^In by MPO was found to be comparable to that with oxine.^99^ MPO was also later used with ^68^Ga for platelet labeling,^68,101^ as well as with ^67^Ga;^102^ however, the labeling efficiency of these agents was shown to be much lower (∼15%) compared with [^111^In]In-MPO (∼80%).^102^

In an interesting study, Ellis et al. synthesized and screened a variety of hydroxypyranones and hydroxypyridinones as bidentate ligands for In^3+^, which formed 3:1 (L:M) complexes with the metal. They identified 3-hydroxy-6-propoxymethyl-4H-pyran-4-one (PMHP; Figure 7A) and 6-butoxymethyl-3-hydroxy-4H-pyran-4-one (BMHP; Figure 7A) as potential ionophores for cell labeling using ^111^In.^103^ A subsequent study showed that these ligands allowed increased cellular uptake of ^111^In (∼90% LE) in mixed leukocytes compared to tropolone (76% LE), with similar efflux rate (approximately 20% after 4 h).^104^ However, radionuclide efflux was not assessed at later time points, which is more relevant for longer-term cell tracking. This may explain the absence of any subsequent reports using these compounds. A similar ligand, ethyl maltol (Figure 7A), was reported as an ionophore for ^89^Zr by Ferris et al. Uptake of the proposed [^89^Zr]Zr(ethyl maltolate)_4_ complex was shown in colon cancer cells (HTC-116) with ∼43% retention after 1 h and with 26% after 24 h.^72^ Because of its less favorable radiolabeling properties compared to [^89^Zr]Zr-oxine, this ligand was not taken any further.

Diethyldithiocarbamate (DEDTC; Figure 7A) was first used as a ligand with ^99m^Tc for cell labeling by Sampson et al. in 1988.^107^ The radiometal complex was proposed to be the bis(ligand)nitrido complex with the Tc/Re core in the 5+ oxidation state (Figure 7A). It was able to radiolabel a crude leukocyte suspension with a LE of ∼73%. *N*-Ethoxy-*N*-ethyl-dithiocarbamate (NOET; Figure 7A) was later used analogously with ^99m^Tc and ^188^Re for leukocyte radiolabeling by Demaimay et al.^109^ Interestingly, radio-HPLC analysis of cell lysates demonstrated that the radiometal complex was still intact, with no release of the radiometal occurring intracellularly. However, this would likely lead to low cellular retention of the compound. Several dithiocarbamates (DEDTC, DMDTC, and DPDTC; Figure 7A) were explored as ionophores for ^64^Cu, likely as the bis(dithiocarbamate) Cu^2+^ complexes (e.g., [^64^Cu]Cu(DEDTC)_2_).^108^ DEDTC exhibited the highest cell labeling efficiency for J774 mouse macrophages with 61−73% LE after just 1 min. The cell uptake of ^64^Cu when using DMDTC and DPDTC was slightly lower with ∼35% and 55% after 30 min, respectively. However, rapid cellular efflux of ^64^Cu was observed with all the dithiocarbamates with cellular retentions between 15–21% after just 20 h,^108^ making these compounds inappropriate for long-term cell tracking.

Demaimay et al. later compared a library of dithiocarboxylate ligands (Figure 7A) for Tc/Re-based cell labeling agents.^110^ The authors first tested the effect of the carboxylate counterion of the ligand on leukocyte labeling using the ^99m^Tc complex of a dithiohexanoic acid ligand. It was found the tetramethylammonium salt was capable of labeling leukocytes, whereas the sodium salt could not. Interestingly, they showed that the LE of leukocytes increased linearly with increasing chain length on the dithiocarboxylate ligand; with ∼25% LE for the 7-carbon chain to ∼65% for the decyldithiocarboxylate ligand.^110^ However, limited data on cellular retention or viability was reported, and hence, it is difficult to assess the effectiveness of these compounds as direct cell labeling agents.

#### 4.2.4 [^99m^Tc]Tc-HMPAO

Another key SPECT radiotracer for direct cell labeling is technetium-99m hexamethylpropylene amine oxime ([^99m^Tc]Tc-HMPAO; Figure 7B). The compound was initially developed for brain imaging because of its lipophilicity (and hence its ability to cross the blood–brain barrier) and its chemical instability (hence its trapping once in the brain).^142^ These properties are the same as those required for cell labeling by the ionophore approach and [^99m^Tc]Tc-HMPAO was first used to label cells in 1986 by Peters et al. for the imaging of leukocytes.^111^ The [^99m^Tc]Tc-HMPAO complex likely exists in the five-coordinate technetium(V) oxo form. The mechanism of trapping within cells relies on the conversion of the complex to a hydrophilic form; however, to the best of our knowledge, neither the structure of this hydrophilic form nor the mechanism of conversion are known. Glutathione has been to shown to convert [^99m^Tc]Tc-HMPAO into a hydrophilic form.^143^ Additionally, it has been shown that liposomes encapsulating glutathione resulted in higher uptake and retention in the aqueous core, consistent with this mechanism.^144^ The main application for [^99m^Tc]Tc-HMPAO was the tracking of leukocytes for the imaging of inflammatory bowel disease,^145^ but since the discontinuation of [^111^In]In-oxine sales in Europe, [^99m^Tc]Tc-HMPAO is now used for most indications in which a leukocyte scan is warranted. Due to the generator production of the radiometal, [^99m^Tc]Tc-HMPAO leukocyte imaging is cheaper and more convenient compared to using [^111^In]In-oxine, and imparts lower radiation doses.^145,146^ However, the shorter half-life of ^99m^Tc (*t*_1/2_ = 6 h) compared to ^111^In (*t*_1/2_ = 2.80 d) limits its use in the long term cell tracking in vivo.

#### 4.2.5 Bis(thiosemicarbazones) with ^64^Cu

One of the earlier ligands investigated for cell labeling with ^64^Cu is the lipophilic, redox-active pyruvaldehyde-bis(*N*^4^-methylthiosemicarbazone) (PTSM). Cu-PTSM exists as an approximate square planar N_2_S_2_ complex (Figures 7A and 8D) which is uncharged due to deprotonation.^131^ The lipophilicity of the Cu(II)-PTSM complex allows it to cross the cell membrane efficiently, while the rate of efflux from cells is controlled by the redox reactivity. Intracellular reduction of Cu(II) to Cu(I) destabilizes the complex, leading to its dissociation and trapping of radioactive copper inside the cell.^147^ However, this release mechanism results in low cellular retention of the isotope. In C6 glioma cells, 36% retention after 5 h was observed,^114^ and efflux studies in the OVA-Th1 cells revealed that 47% of [^64^Cu]Cu-PTSM remained after 5 h and only 14% after 24 h.^117^ A similar trend was observed by Charoenphun et al., who prepared the copper complexes of several bis(thiosemicarbazones) (GTS, GTSM, PTS, and PTSM; Figure 7A). Cellular uptake in J774 mouse macrophages of ^64^Cu plateaued at 50–60% LE for all of the radiometal complexes. However, rapid cellular efflux of ^64^Cu was observed with all ligands with cellular retentions between 14–28% after 20 h.^108^ This low cellular retention is likely due to copper cellular transport mechanisms (see Section 5.2) and may limit the use of these compounds for long-term cell tracking. [^64^Cu]Cu-PTSM was later compared with ^64^Cu labeled poly(ethylenimine) (^64^Cu-PEI; Figure 7B) for cell labeling.^116^ PEI has been used as a gene carrier and can enter cells via endosomes, by becoming cationic via amine protonation.^148^ In vitro studies showed that [^64^Cu]Cu-PTSM uptake into cells was much greater compared to ^64^Cu-PEI (70–80% and 20%, respectively, after 3 h), and also had approximately half the radiation efflux after 27 h. However, the PEGylation of ^64^Cu-PEI (^64^Cu-PEI-PEG) partially ameliorated these issues.^116^

### 4.3 Cell Surface Labeling

**Figure 10:**
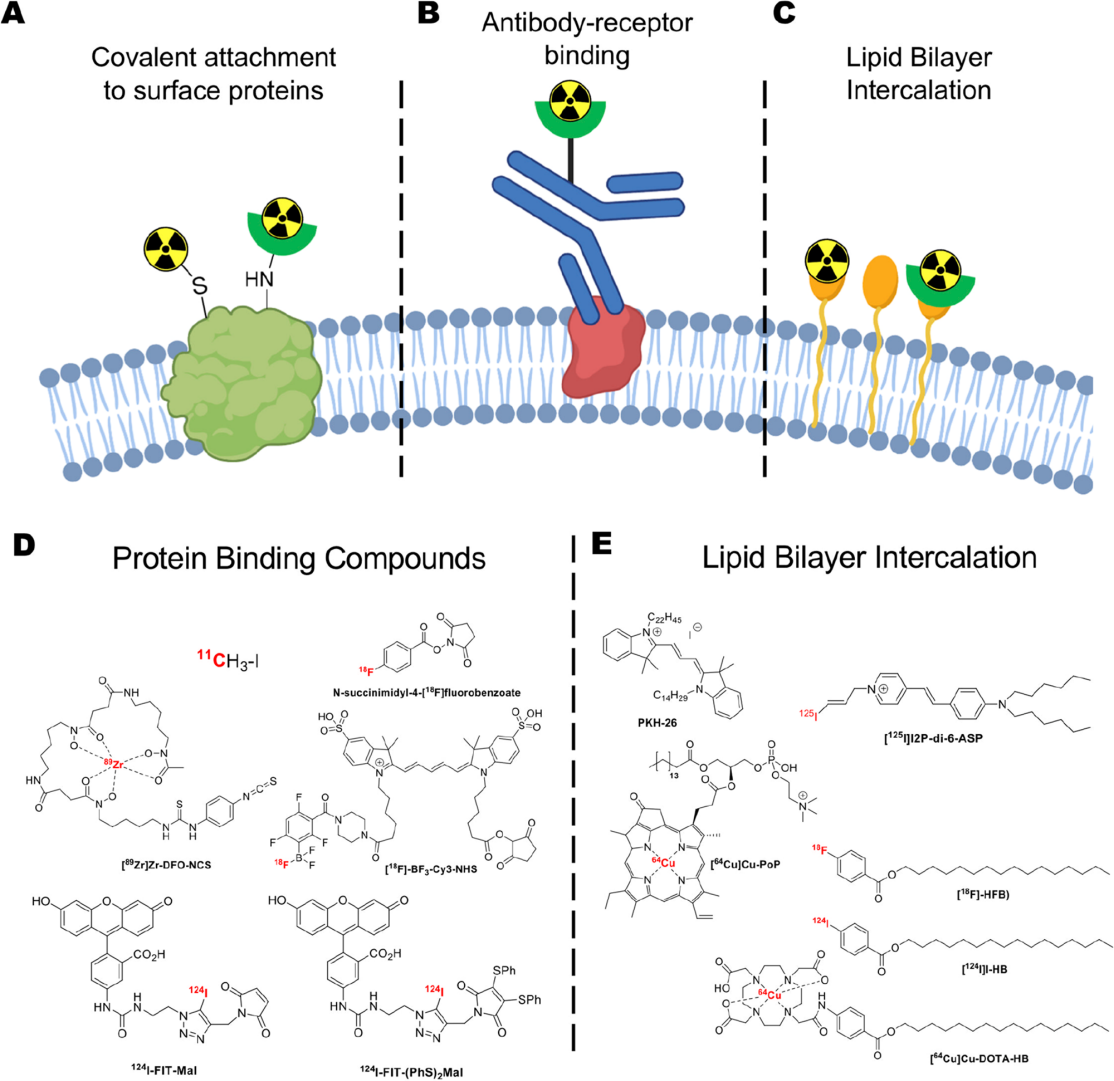
Schematic showing the three main methods used for radiolabeling of the cell surface for direct cell radiolabeling. (A) Radionuclides can be covalently attached to surface proteins or (B) radiolabeled antibodies can bind to receptors on the cell surface. Additionally, (C) compounds can be designed to intercalate into the lipid bilayers on the cells surface allowing radiolabeling. (D) Structures of the radioactive compounds used for covalent attachment to the cell surface. (E) Structures of radioactive compounds that can intercalate into the lipid bilayer on cells allowing radiolabeling. Panel A was made using Biorender.com.

The transport of radionuclides into cells using ionophore ligands is clearly a successful and widely used strategy. However, the potential radiotoxicity associated with the delivery of ionizing radiation-emitting radionuclide intracellularly (see section 5.3) is often stated as a concern. A potential (although as yet unproven) way of mitigating this effect is by radiolabeling cells on the cell membrane, further away from the nucleus which would likely reduce the toxicity of Auger-electrons (but not gamma photons) emitted by some radionuclides (e.g., ^111^In, ^123^I).^149^ The radiotoxicity of a cell labeling agent is both radionuclide- and cell-dependent, and hence, more research is needed in the field of radiobiology to establish the effects of cell-radiolabel location on radiotoxicity. Regardless, the chemical structure of the cell membrane easily allows the binding and association of a variety of different compounds (Table 4) through various interactions (Figure 10). In this section, we will discuss the main methods used for the direct labeling of cells via their plasma membrane.

**Table 4:** Table Summarizing the Various Methods of Cell Surface Labeling and Cell Radiolabeling Agents Used for Direct Cell Labeling, along with Their Corresponding Radionuclides and the Cell Type Labeled

cell radiolabeling method	cell labeling agent	radionuclide	cell type labeled	ref
surface protein binding	methyl iodide	^11^C	natural killer (NK) cells	(150, 151)
	*N*-succinimidyl-4-fluorobenzoate	^18^F	bone-marrow-derived dendritic cells (BMDCs)	(152)
	NHS ester-functionalized cyanine dye	^18^F	RBCs	(153)
	*p*-isothiocyanato-benzyl-desferrioxamine (DFO-NCS)	^89^Zr	melanoma cells; mesenchymal stem cells; dendritic cells	(154)
			cardiopoietic stem cells	(155)
	maleimide-functionalized fluorescent dye	^124^I	Jurkat cells	(156)
	dithiophenolmaleimide-functionalized fluorescent dye			
antibody-receptor binding	anti-CD45 antibodies	^89^Zr	human peripheral blood stem cells (hPBSCs)	(157)
		^64^Cu		
	internalizing TCR antibody	^64^Cu	chicken ovalbumin (cOVA)-TCR transgenic T cells	(158)
Lipid bilayer intercalation	optical dye PKH-26 derivative	^125^I	macrophages	(159)
	iodo-(dialkylaminostyryl)pyridinium dyes	^131^I	lymphocytes, leukocytes	(160)
		^125^I	lymphocytes, leukocytes	
			splenocytes	(161)
	porphyrin-phospholipid conjugate	^64^Cu	RBCs	(162)
	hexadecylbenzoate-conjugates	^18^F	MSCs	(163)
			progenitor cells	(164)
			breast cancer cells (MDA-MB-231): Jurkat cells	(165)
		^124^I	ADSCs	(166)
		^64^Cu	ADSCs	(167)

#### 4.3.1 Cell Surface Protein Binding

An early method for cell surface labeling was to radiolabel proteins present on the cell surface (Figure 10A) as reported by Melder et al., who used [^11^C]CH_3_I (Figure 10D).^150,151^ Nonradioactive CH_3_I is a commonly used methylation agent capable of attaching a methyl group to variety of functional groups (amines, thiols, carboxylates) via the S_N_2 substitution reaction. The fact that some of these functional groups are present on cell membranes allowed the use of [^11^C]CH_3_I to radiolabel natural killer (NK) cells. While the labeling efficiency of [^11^C]CH_3_I was not reported, the attachment of the tracer to the cell surface (cellular retention) was shown to be stable (>90%) over the 60 min tested. Additionally, the radiolabeling method was shown to have little effect on the cell viability and cytotoxic activity of the NK cells.^151^ However, the short half-life of ^11^C (*t*_1/2_ = 20 min) considerably limits the PET imaging window and is a major drawback for cell labeling; in this case imaging was performed up to 60 min.^150^

**Figure 11:**
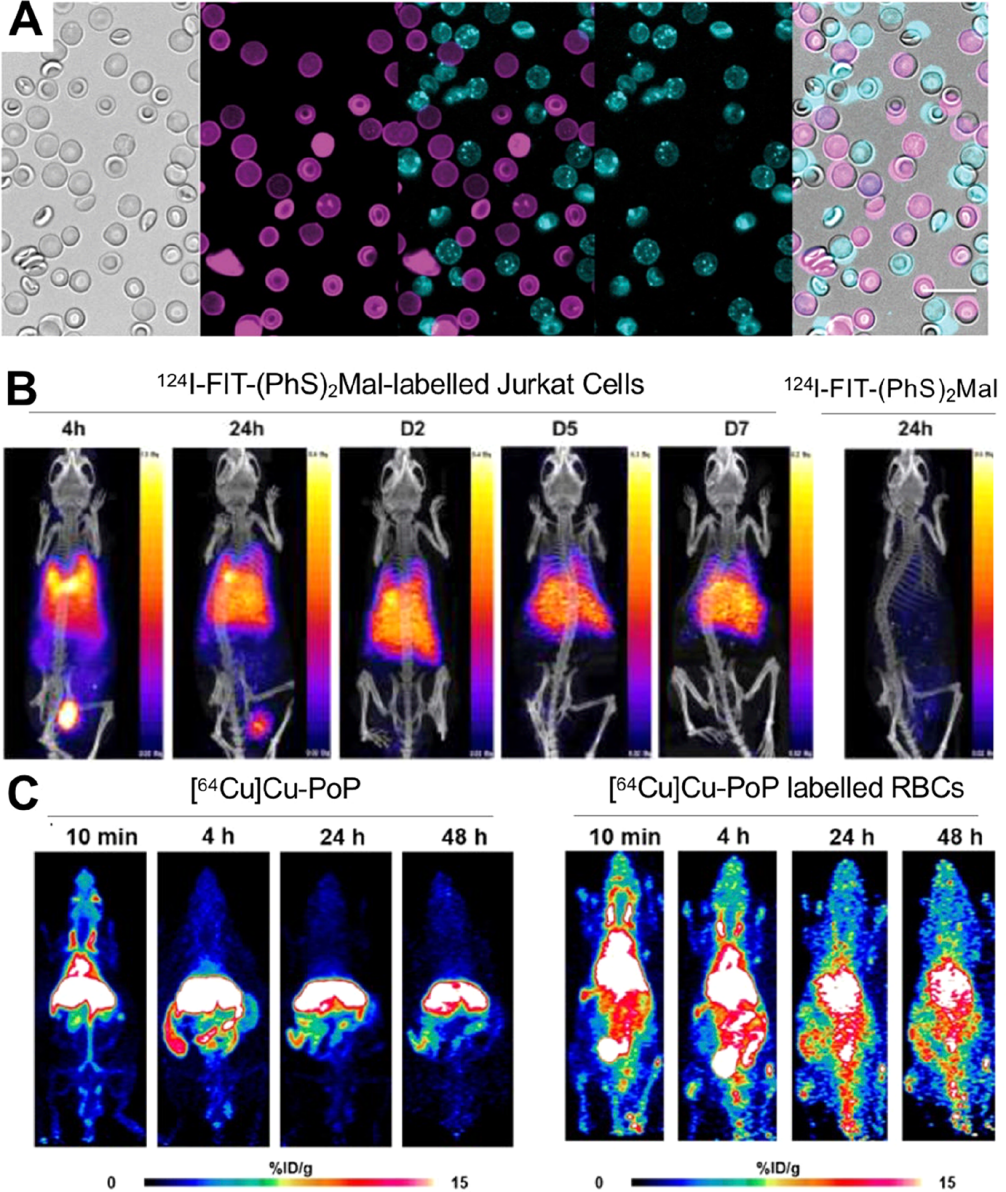
(A) Microscopy images of RBCs labeled with either Cy3 or Cy5 dyes based on [^18^F]BF3-Cy3-NHS. Bright field imaging of the RBC-Cy3/RBC-Cy5 mixture (far left). Middle left image: RBC-Cy3s and middle right image is for RBC-Cy5. Middle image is an overlay of the RBC-Cy3 and RBC-Cy5 showing a lack of spectral overlap between the two fluorophores, and no mixing of fluorophores between cells after 14 h. Far left image is an overlay of bright field and fluorescent images. Adapted with permission from Wang et al., ref (153). Copyright 2017 SAGE Journals. (B) PET/CT images of NSG mice that received ^124^I-FIT-(PhS)_2_Mal labeled Jurkat cells at 4 and 24 h and 2, 5, and 7 days or ^124^I-FIT-(PhS)_2_Mal at 24 h post IV injection. Adapted with permission from Pham et al., ref (156). Copyright 2020 American Chemical Society. (C) PET image of mice injected with ^64^Cu-labeled porphyrin-phospholipid conjugate (PoP) (left) or ^64^Cu-labeled PoP RBCs (right). RBCs were obtained from mice prior to labeling and intravenous injection. Adapted with permission from Kumar et al., ref (162). Copyright 2021 Kumar et al. Published by Wiley-VCH GmbH under CC License [https://creativecommons.org/licenses/by/4.0/].

The cell surface labeling method was later expanded by Olasz et al., who used *N*-succinimidyl-4-[^18^F]fluorobenzoate ([^18^F]SFB; Figure 10D) to radiolabel cells via amine residues on their surface.^152^ It was shown that bone marrow-derived dendritic cells (BMDCs) could be radiolabeled with the agent with a cell labeling efficiency of ∼20%. Interestingly, the cellular retention of the radiotracer was shown to be lower at 37 °C than at 4 °C (44% and 91%, respectively, after 4 h), suggesting that this tracer is removed from the cells through membrane turnover or a metabolic process rather than passive efflux. A variation of this method, incorporating a fluorescent cyanine dye (Cy3 or Cy5), was reported by Wang et al. for RBC radiolabeling via amine residues.^153^ The compound was radiolabeled via reaction of a dioxaborolane precursor with [^18^F]F^–^ forming the trifluoroborate [^18^F]BF_3_-Cy3-NHS (Figure 10D) Interestingly, the authors showed that the dye was stably attached to the cell surface and not transferred to neighboring cells. RBCs labeled with each of the two NHS dyes were mixed together and left for 14 h, after which fluorescence microscopy showed the absence of spectral overlap between the two fluorophores (Figure 11A), demonstrating that there was no mixing of fluorophores between cells. Despite this, cell radiolabeling with this compound was inefficient with only ∼2% (actual value not reported) of added activity associated with RBCs after labeling. This may be due to the lack of isolation and purification of the [^18^F]BF_3_-Cy3-NHS radiolabeled agent before its use in the cell labeling procedure. Additionally, high bone uptake could be seen in PET images of the radiolabeled RBCs suggesting release of the radionuclide as [^18^F]F^–^ from [^18^F]BF_3_-Cy3-NHS/RBCs in vivo.

Bansal et al. developed a ^89^Zr-based cell labeling agent using an isothiocyanate derivative of the chelator desferrioxamine (DFO).^154,155^ The isothiocyanate group of [^89^Zr]Zr-DFO-NCS (Figure 10D) most likely reacts with free amines present on the cell surface to form a thiourea linkage. This technique demonstrated good labeling efficiency (30–55%, depending on cell type), and excellent retention of radioactivity over 7 days.^154^ In vivo PET imaging showed distinct differences between the distribution of [^89^Zr]Zr-DFO-NCS labeled cells and that of unchelated ^89^Zr. However, the authors did not investigate the in vivo biodistribution of the [^89^Zr]Zr-DFO-NCS as a negative control, although this compound is likely to be rapidly excreted. Understanding the biodistribution of stable cell surface labeling agents is needed to confirm that the PET signal observed when performing in vivo cell tracking relates to that of labeled cells.

Similarly, Pham et al. reported two dual modality PET/fluorescent cell labeling agents comprising of a hydrophilic fluorescein dye conjugate containing ^124^I with either a maleimide (^124^I-FIT-Mal; Figure 10D) or dithiophenolmaleimide (^124^I-FIT-(PhS)_2_Mal; Figure 10D) moiety for cell labeling via free thiol groups on membrane proteins.^156^^124^I-FIT-(PhS)_2_Mal had much higher LE than ^124^I-FIT-Mal and was chosen for further evaluation. Labeling efficiency was further increased by pretreating cells with tris(2-carboxyethyl)phosphine (TCEP), a disulfide bridge reducing reagent, confirming that conjugation occurred via free thiol groups on the membrane. Fluorescence microscopy confirmed tracer binding to the cell surface. Cellular retention of ^124^I-FIT-(PhS)_2_Mal was high with >65% still associated with cells after 7 days.^156^ In vivo PET imaging of Jurkat cells labeled with ^124^I-FIT-(PhS)_2_Mal showed uptake in the bladder was observed at 4 and 24 h (Figure 11B), suggesting urinary clearance of ^124^I-FIT-(PhS)_2_Mal released from cells. Assessment of the in vivo release of iodide by this radioiodine-based tracer using thyroid radioactivity uptake as a marker was not possible as the animals were pretreated with potassium iodide to block uptake of any free ^124^I. The expected distribution of the cells was observed, with initial uptake in the lungs followed by gradual redistribution to the liver and spleen (Figure 11B); the labeled cells showed a biodistribution that was distinct from administered ^124^I-FIT-(PhS)_2_Mal which was rapidly excreted (Figure 11B) indicating good in vivo stability of the compound on cells.

**Figure 12:**
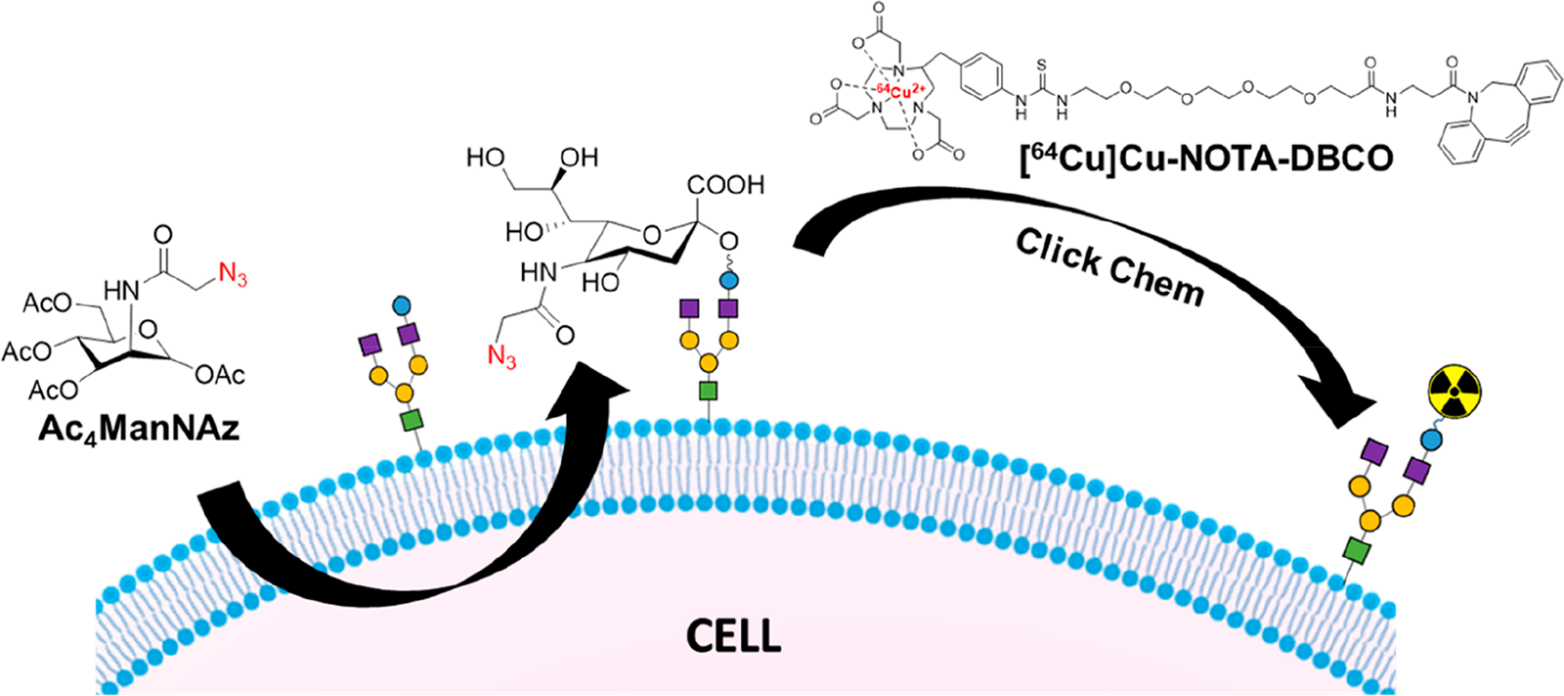
Schematic overview of metabolic glycoengineering approached used by Lu et al.^168^ The OVA-CTLs were metabolically modified with azide-linked oligosaccharides which allowed radiolabeling of the cells with the [^64^Cu]Cu-NOTA-DBCO click component.

An interesting approach for surface labeling was recently reported by Lu et al., who used metabolic glycoengineering biosynthesis to incorporate reactive groups on the surfaces of cells. Chemically modified monosaccharides with non-natural functional groups have been shown to hijack the glycosylation pathways in mammalian cells, leading to the presentation of modified glycans on the surface.^169^ The authors used this methodology to incorporate azide-functionalized oligosaccharides on the surface of CTLs by first pretreating them with the monosaccharide Ac_4_ManNAz for 24 h to generate azide groups, and then labeling with radioactive biorthogonal click component [^64^Cu]Cu-NOTA-DBCO (Figure 12).^168^ The cell labeling was shown to be specific for the glycoengineered cells with approximately three times higher LE for CTLs treated with the monosaccharide than for untreated cells. Additionally, the cellular retention of the bound [^64^Cu]Cu-NOTA-DBCO was high, with <20% efflux of ^64^Cu after 48 h. While this method may be unnecessarily complicated for direct cell labeling and tracking, glycoengineering could be used as a basis for indirect cell labeling: azide-functionalized cells could potentially be imaged longitudinally using bioorthogonal tracers.

#### 4.3.2 Antibody–Receptor Binding

Although cell labeling with antibodies is usually performed in vivo (i.e., radiolabeled antibodies are administered intravenously to accumulate on the target cells), it is also possible to directly label cells with antibodies in vitro before infusing them (Figure 10B). Depending on the antibody and its target, it may remain on the cell surface or be internalized. A useful review covering the various parameters affecting the fate of antibodies in vivo was recently written by Thomas and Balthasar.^170^ For example, one study using ^64^Cu- and ^89^Zr-labeled anti-CD45 antibodies (as TETA or DFO conjugates, respectively) showed no internalization by human peripheral blood stem cells (hPBSCs), and the higher cell labeling efficiency observed with [^89^Zr]Zr-DFO-CD45 (2000-fold higher in terms of μCi/cell;% LE not reported) compared with [^64^Cu]Cu-TETA-CD45. This suggests the cell labeling was affected by the antibody-chelator conjugation method.^157^ On the other hand, Griessinger et al. reported the use of radiolabeled antibodies that could bind to the surface receptors on T-cells and be internalized. The cell labeling properties were compared with [^64^Cu]Cu-PTSM.^158^ The uptake of ^64^Cu-labeled internalizing antibodies was found to be three-times lower than that of [^64^Cu]Cu-PTSM (∼14% and 46%, respectively). However, the radiolabel retention with ^64^Cu-labeled internalizing antibodies was superior with ∼74% retained activity after 48 h compared to <10% for cells labeled with [^64^Cu]Cu-PTSM.^158^ This is likely due to the higher stability of the [^64^Cu]Cu-DOTA complex compared to [^64^Cu]Cu-PTSM. Additionally, the internalized antibodies are mostly sequestered within the endosomal/lysosomal compartment (see section 5.2.2), reducing the availability of ^64^Cu for endogenous export mechanisms. Despite this, the internalizing antibodies also caused a significant reduction in cell viability with a ∼40% loss in cell viability after 48 h at the lowest level of activity used, limiting the use of this cell labeling method.

#### 4.3.3 Lipid Bilayer Intercalation

As an alternative to attaching radiotracers to the surface of cells via covalent bonds to proteins, carbohydrates, or receptor binding mechanisms, direct cell labeling agents can be designed to intercalate into the lipid bilayer of cell membranes (Figure 10C). An early example of this approach was the compound ^125^I-PKH-95; a radioiodinated derivative of the lipophilic optical dye PKH-26 (Figure 10E) developed in the early 1990s.^171^ It was hypothesized that the long alkyl chains present on the compound would allow “anchoring” of the complex into the cellular membrane. One study showed better cellular retention of ^125^I-PKH-95 in macrophages compared with [^111^In]In-oxine.^159^ Similarly, a study using a series of iodo-(dialkylaminostyryl)pyridinium dyes radiolabeled with ^125^I/^131^I for the radiolabeling of leukocytes showed that the compounds with longer alkyl chains (*n* = 8–10) were less efficient cell labeling agents than those with dibutyl or dihexyl chains.^160^ The suggested reason was the aqueous insolubility and possible micelle formation, of the compounds with longer chains. The lead candidate1-[e-3-[^125^1]iodo-prop-2-enyl]-4-[4-(dihexylamino)styryl-pyridinium ([^125^I]I2P-di-6-ASP; Figure 10E) was later taken forward and used by Albright et al. to radiolabel splenocytes.^161^ One potential drawback of using radiolabeled dyes that integrate into membranes is their transfer to neighboring cells, leading to misleading imaging signal. This has previously been shown to occur in vivo with stem cells labeled with a variety of lipophilic fluorescent dyes, including PKH26.^172^ Although this phenomenon, to the best of our knowledge, has not been demonstrated with radioactive analogues of these dyes, it is highly likely to occur as well. Similarly, Kumar et al. described the radiolabeling of red blood cells using a porphyrin-phospholipid conjugate (PoP).^162^ The porphyrin macrocycle ring allowed chelation with ^64^Cu ([^64^Cu]Cu-PoP; Figure 10E) and hence the radiolabeling of RBCs and their imaging with PET. Membrane exchange was shown to occur with nonradioactive PoP in vitro when incubating unlabeled RBCs with RBCs labeled with the porphyrin conjugate, although this was not tested on RBCs radiolabeled with [^64^Cu]Cu-PoP. However, while in vivo, PET imaging showed that radiolabeled RBCs had a distinct biodistribution from the free [^64^Cu]Cu-PoP agent (Figure 11C), the circulation time of the labeled RBCs was lower than expected, suggesting loss of the PoP agent in vivo or low cell viability of the labeled RBCs.^162^

Alternative tracers for cell surface labeling include hexadecyl-4-[^18^F]fluorobenzoate ([^18^F]HFB), [^64^Cu]Cu-DOTA-hexadecylbenzoate ([^64^Cu]Cu-DOTA-HB), and hexadecyl-4-^124^I-iodobenzoate ([^124^I]HIB) (Figure 10E). [^18^F]HFB allowed labeling of mesenchymal stem cells (MSCs) with 25% LE and >90% retention up to 4 h^163^ and enabled the visualization of progenitor cells in the heart for up to 4 h.^164^ [^124^I]HIB and [^64^Cu]Cu-DOTA-HB labeled cells showed moderate labeling efficiencies, with high retention of activity for [^124^I]HIB (>90% up to 24 h).^166,167^ Overall, tracers that label the surface by noncovalent insertion into the cell membrane, such as [^18^F]HFB and [^124^I]HIB, showed much higher retention than the protein-binding *N*-succinimidyl-4-[^18^F]fluorobenzoate ([^18^F]SFB)^163^ and FDG.^164,166^ [^124^I]HIB could also image adipose-derived stem cells in the heart for 3–9 days,^166^ whereas FDG was rapidly taken up by neighboring tissue. The mechanisms behind the difference in retention between the cell surface labeling agents [^18^F]HFB/[^124^I]HIB and [^18^F]SFB were not explored, but it is possible than protein-rich areas of the membrane (to which [^18^F]SFB is more likely to bind) are more frequently recycled or that surface protein-bound radiolabels are cleaved by extracellular proteases. [^18^F]HFB was found to preferentially bind to disrupted membrane fragments on dead cells over live intact cells.^165^ This could be a potential drawback for in vivo cell tracking with this agent, as dead cells can have different biodistribution profiles compared to live cells, leading to misinterpretation of the images.^3^ In general, validation of the membrane intercalation method for cell radiolabeling is still lacking. Labeling efficiency is often either low or not reported and in vitro cellular retention of the radiotracers over long periods of time (several days) is not known. This, coupled with the potential issue of membrane transfer with these compounds, may explain why this method has not found widespread use compared with other labeling methods.

### 4.4 Other Small Molecule-Based Methods

As we have previously discussed, small molecular weight compounds can be used for the ex vivo radiolabeling of cells; either via the passive transport across the membrane or by direct attachment to the cell surface itself. However, other small molecules can be trafficked into cells through passive or active transport mechanisms and converted into hydrophilic forms via intracellular pathways reducing the ability of the radionuclide to diffuse out of cells (Figure 6C). Table 5 summarizes the various small-molecule-based direct cell radiolabeling methods discussed in this section.

**Table 5:** Summary of the Various Small-Molecule-Based Cell Radiolabeling Agents Used for Direct Cell Labeling, along with Their Corresponding Radionuclides and the Cell Type Labeled

cell labeling agent	radionuclide	cell type labeled	ref
[^51^Cr]Na_2_CrO_4_	^51^Cr	RBCs	(174)
		leukocytes	(175, 176)
[^18^F]fluoro-2-deoxy-d-glucose ([^18^F]FDG)	^18^F	granulocytes	(177)
		progenitor cells; mesenchymal stem cells	(178)
		cardiac stem cells	(179)
		human vascular endothelial cells (HUVECs)	(180)
		mesenchymal stem cells	(181)
3′-deoxy-3′-L-[^18^F]-fluorothymidine ([^18^F]FLT)	^18^F	breast cancer cells (MDA-MB-231)	(182)
		HUVECs	(180)
5-[^124^I]iodo-2′-deoxyuridine ([^124^I]IdU)	^124^I	OT-I T cells	(183)
sulfonamide derivatives	^11^C	RBCs	(184)
	^18^F		

One of the earliest direct cell labeling methods was the use of radioactive chromate ([^51^Cr]Na_2_CrO_4_; *t*_1/2_ = 27.7 d) for the labeling of RBCs/erythrocytes, first reported in 1950,^174^ and used for the radiolabeling of leukocytes in 1955—but not for imaging.^175^ While the exact mechanism of cell labeling is not known, it has been shown that intracellular ^51^Cr is primarily in the 3+ oxidation state and bound to proteins,^185^ suggesting reduction of the chromate ion occurs intracellularly. The chromic ion has been shown to bind to the β-globin chain of intracellular hemoglobin in erythrocytes.^186^ However, it is likely the radiometal can bind to other intracellular macromolecules as well. This mechanism may also depend on cell type. For example, it was shown that leukocytes have a highly specific transport mechanism for [^51^Cr]Na_2_CrO_4,_ with uptake being reduced by the use of nonradioactive chromate and metabolic inhibitors; other divalent anions only slightly inhibited uptake.^176^ However, this cell labeling method is not appropriate for in vivo cell tracking as ^51^Cr is not suitable for imaging because of the low gamma-ray yield (10%, 0.32 MeV) emitted from the isotope and long half-life (27.7 d), leading to a significantly higher dose compared with other radionuclides.

**Figure 13:**
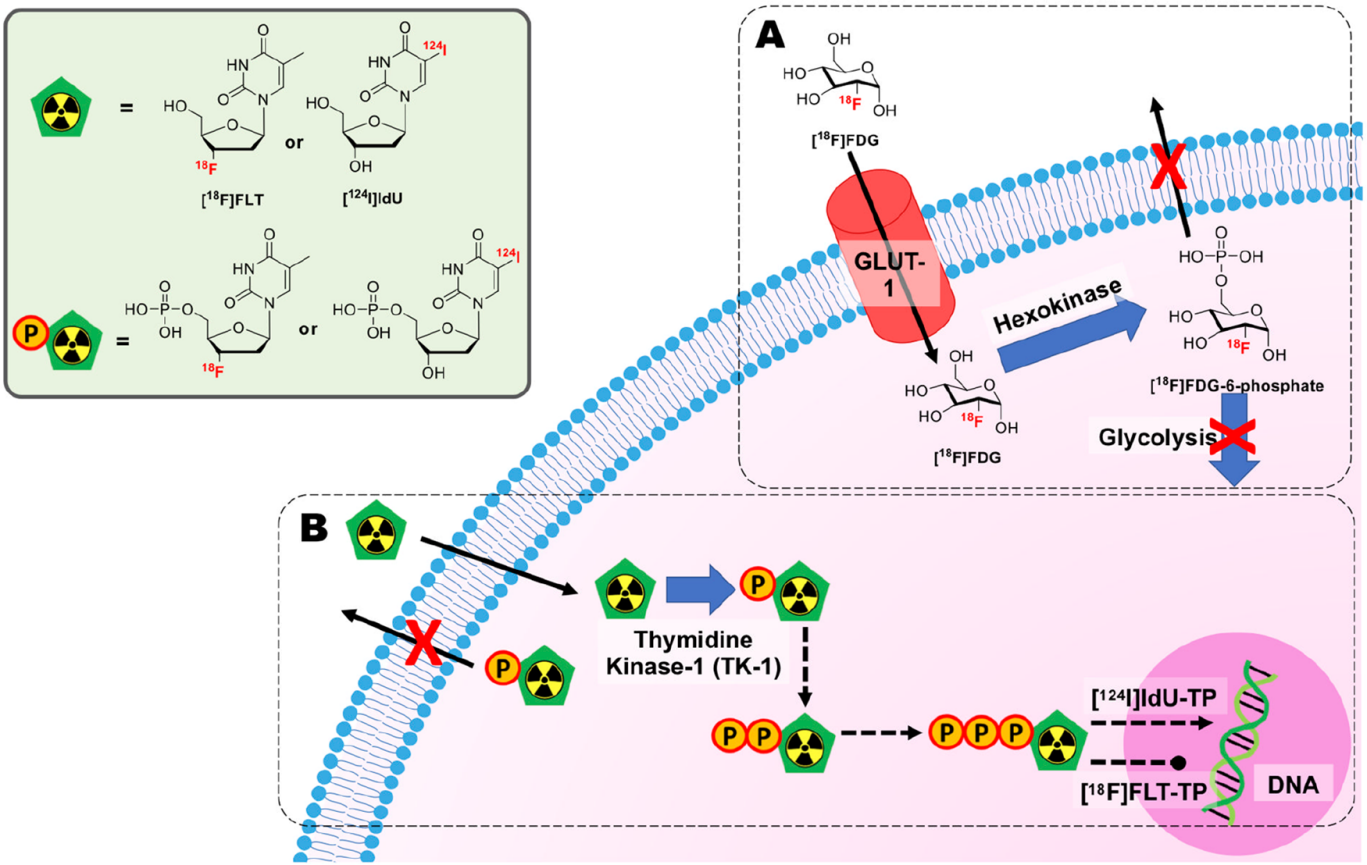
(A) Schematic representation of the cellular uptake mechanism of [^18^F]FDG. [^18^F]FDG is taken up into cells via glucose transporter 1 (GLUT-1) and phosphorylated by hexokinase, preventing it from diffusing out of cells. (B) Structures of [^18^F]FLT and [^124^I]IdU (green box); and schematic representation of cellular uptake and trapping in cells. The compounds enter cells and are phosphorylated by thymidine kinase (TK-1) preventing their escape from cells; the compounds can potentially be further phosphorylated. [^124^I]IdU may be incorporated into DNA, however this is limited for [^18^F]FLT as it acts as a chain terminator because of the absence of the 3′-hydroxyl in its structure.^173^

**Figure 14:**
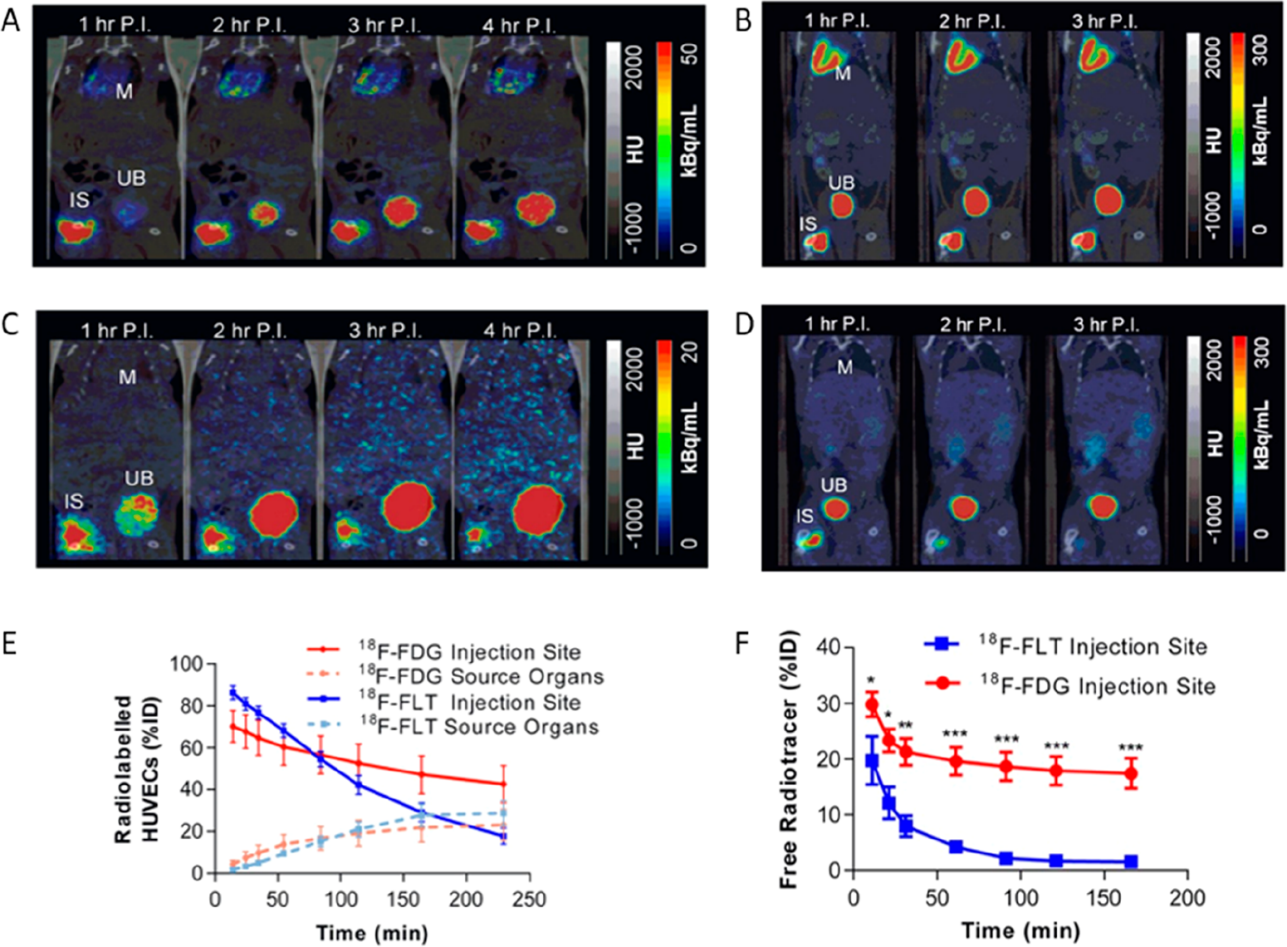
Comparison of human vascular endothelial cells (HUVECs) labeled with [^18^F]FDG (A) and [^18^F]FLT (C) or free [^18^F]FDG (B) and [^18^F]FLT (D) injected at the same site, representative averaged (1 h) images from each hour postinjection (P.I.). IS: Injection site. M: Myocardium. UB: Urinary bladder. (E, F) Time-activity curves for ^18^F-labeled cells and the corresponding radiotracers. The persisting signal at the injection site with [^18^F]FDG-labeled cells is partly due to [^18^F]FDG leaking from the labeled cells and being taken up by neighboring tissue. Note the increasing ^18^F signal in the heart region, indicating release of [^18^F]FDG into the circulation. In contrast, free [^18^F]FLT is rapidly cleared from neighboring tissue. Consequently, the signal from [^18^F]FLT-labeled cells is more representative of the presence of labeled cells at the injection site. Adapted with permission from Macaskill et al., ref (180). Copyright 2017 Springer Nature under CC License [https://creativecommons.org/licenses/by/4.0/].

A relatively simple method for cell labeling takes advantage of the trapping mechanism of [^18^F]fluoro-2-deoxy-d-glucose ([^18^F]FDG) used in the vast majority of clinical PET scans. [^18^F]FDG is transported across the cell membrane and into the cytoplasm via GLUT-1 transporters and is phosphorylated within the cell by hexokinase to [^18^F]FDG-6-phosphate (Figure 13A). Hence, direct cell labeling can be blocked by the presence of nonradioactive glucose.^181^ With a fluorine atom instead of a hydroxy group on the second carbon, [^18^F]FDG-6-phosphate cannot be isomerized and metabolized further and is trapped intracellularly. However, the retention of ^18^F inside most leukocytes and stem cells is poor, as [^18^F]FDG-6-P undergoes dephosphorylation back to [^18^F]FDG, leading to the release of 20–40% of the original activity within an hour.^113,177,178,181,187,188^ This does not preclude the use of direct cell tracking with [^18^F]FDG for in vivo imaging applications within a short time frame—there have been several clinical studies using [^18^F]FDG-labeled cells^189^ (section 6), but this radiotracer has yet to see routine use as a cell labeling agent. Furthermore, released [^18^F]FDG is then taken up by neighboring tissue cells, leading to an artificial increase in signal which is not a true reflection of the presence of the administered cells (Figure 14). This issue, along with low cellular retention of [^18^F]FDG and the short half-life of ^18^F, likely limits the use of this radiotracer for cell tracking despite its broad availability in the clinic.

An example of released [^18^F]FDG being taken up by neighboring tissue was elegantly illustrated in a comparison with human endothelial cells (HUVECs) labeled with 3′-deoxy-3′-L-[^18^F]-fluorothymidine ([^18^F]FLT; Figure 13). Uptake of [^18^F]F-FLT into cells is proposed to occur via thymidine kinase 1 (TK1) activity. [^18^F]F-FLT is converted through TK1 and other enzymes into [^18^F]F-FLT-diphosphate and [^18^F]F-FLT-triphosphate, which are then trapped inside the cell (Figure 13B). Additionally, [^18^F]FLT uptake is dependent on the cell cycle, with higher activity during the S-phase (DNA synthesis) during which the expression of thymidine kinase 1 (TK1) is increased.^182^ Consequently, uptake is limited to actively dividing cells and [^18^F]FLT is less likely to be incorporated by surrounding tissue (Figure 14). 12% LE was achieved, and the cellular retention of the radioactivity was shown to be ∼80% up to 60 min. Radiolabeling had no apparent effect on cell viability, proliferation, or structure. While cell labeling and tracking with [^18^F]FLT clearly has advantages over [^18^F]FDG, the method may be limited by the low labeling efficiency and the short half-life of ^18^F.

A similar cell labeling method was reported by Agger et al. with the radiolabeled thymidine analogue 5-[^124^I]iodo-2′-deoxyuridine ([^124^I]IdU, Figure 13B), which can be incorporated into DNA intracellularly during DNA replication.^183^ No data on cellular uptake or retention was reported in this study. However, using a DNA-binding radiotracer for cell tracking purposes is a potentially risky strategy due to the potential damage to DNA molecules (see section 6). Agger et al. reported cell viability of 71–90% for OT-I spleen cells with little detail on how this was measured. The authors showed that radiolabeled cells had similar tumor accumulation to nonradiolabeled cells based on flow cytometry analysis. However, no evaluation of DNA damage to radiolabeled cells was carried out.

Finally, ^123^I-, ^125^I-, ^11^C-, and ^18^F-labeled sulfonamide derivatives have been shown to specifically radiolabel RBCs in vitro and in vivo by targeting carbonic anhydrase II (CA II), a metalloenzyme expressed on RBCs.^184,190−192^ Although the radioiodine compounds were tested in the clinic, there does not appear to have been widespread use of this class of molecules for labeling of RBCs and use as blood pool imaging agents.

### 4.5 Particle Uptake

Finally, while a vast portion of the direct cell labeling literature focuses on small molecules, larger compounds are capable of direct cell labeling. The uptake of radioactive particles (colloids and nanoparticles; Figure 6D) has also been explored. While the size and shape of radiocolloids may vary greatly between tens of nanometers to several micrometers,^193^ nanoparticle-based methods use particles with a generally smaller and homogeneous size for radiolabeling. Additionally, nanoparticle uptake into cells can be modified by the use of coatings and external membrane permeabilizing agents. On the basis of these properties, we will discuss colloids and nanoparticles in separate subsections.

#### 4.5.1 Colloids

Radioactive colloids have been known since the 1950s as effective direct cell radiolabeling agents. Following the discovery that colloidal matter is quickly taken up by phagocytic cells in the liver, spleen, and bone marrow after systemic administration, pioneering work by Ganz et al.^194^ and Gosselin et al.^195^ using ^198^Au colloids demonstrated that this uptake was mediated by phagocytosis. This was quickly identified as a useful method to directly label cells for in vivo cell tracking using nuclear imaging (Figure 6D).

After this early work using ^198^Au colloids, focus shifted to ^99m^Tc because of its excellent emission properties for gamma imaging, and availability from benchtop generators. Gillespie et al. first evaluated ^99m^Tc radiolabeling of a series of cells of mouse and human origin by in situ reduction of [^99m^Tc]TcO_4_^–^ using stannous chloride.^196^ The exact radiolabeling mechanism using this methodology was not investigated, but it is highly likely that ^99m^Tc incorporated into the cells via two mechanisms: (i) direct binding of reduced forms of Tc (likely Tc^5+^ or Tc^3+^) to cell membrane components or (ii) by formation of Sn–Tc colloids that [^99m^Tc]TcO_4_^–^ can form when being reduced with large amounts of stannous salts, possibly assisted by the presence of Na_2_CrO_4_. Radiolabeling yields using this methodology were consistently high and the authors demonstrated the ability of the labeled cells to synthesize DNA. Interestingly, the presence on Na_2_CrO_4_ increased cell labeling efficiency by ∼30%, allegedly by the pertechnetate carrier effect of the chromate anion, although more experiments would have been required to prove this is the case. Furthermore, they reported in vivo cell tracking of murine cancer cells (murine fibrosarcoma Sa I) for the first time, finding they distributed to the lungs, liver, and spleen after intravenous administration. Ferrant et al. also used this technique to radiolabel red blood cells and evaluated it in patients for the first time in comparison with the then-standard method based on ^51^Cr.^197^

White blood cells (WBC) have also been radiolabeled using reduced ^99m^Tc via Sn reduction, that as mentioned above is likely to be mediated by ^99m^Tc–Sn colloids. Being able to image autologous WBCs is a useful method to diagnose infections/inflammation. Linhart et al. explored this concept in vitro, showing radiolabeling yields of 30% and satisfactory functional activity tests (e.g., chemotaxis) postradiolabeling.^198^ Kelbaek et al. refined this methodology for WBC radiolabeling, exploring different amounts of Sn salts (SnF_2_ and SnP_2_O_7_), and confirming retainment of cell function after radiolabeling.^199^

An important report in this area identified the factors that control ^99m^Tc WBC labeling by phagocytic uptake of Tc–Sn colloids. The size and shape of Tc–Sn colloids can vary greatly;^193,200^ it was found that the most important factor for reproducibly labeling WBCs using this technique was a mean particle size of 2.1 μm.^193,201^ Using ^99m^Tc-SnF_2_, Puncher et al. used autoradiography of smears and frozen sections of labeled cell suspensions to show that this colloid was selective for neutrophils when radiolabeling leukocyte-rich plasma, and that erythrocytes were the cell type most highly radiolabeled when performing this procedure in whole blood.^202^ Interestingly, autoradiographs identified two distinct labeling mechanisms: one that is stable and where the radioactivity was diffuse and intracellular (predominant in neutrophils and monocytes) and another one where the radioactive particles were weakly bound at the cell membrane in localized spots (predominant in red blood cells and lymphocytes). Additionally, they reported that the phagocytic inhibitor cytochalasin B showed no effect on cell labeling of neutrophils with SnF_2_ and ^99m^Tc, suggesting phagocytosis was not the mechanism of uptake. However, they noted the amounts used may have only partly inhibited the phagocytosis, with the cells in high excess compared to the labeling agent to still allow uptake.^202^ An interesting comparison between ^99m^Tc-labeled leukocytes (via ^99m^Tc-SnF_2_ colloids) and ^111^In-labeled leukocytes (via [^111^In]In-oxine) for imaging abdominal infection in patients was reported by Carter et al.^203^ This study concluded that ^99m^Tc-SnF_2_ colloid labeling of leukocytes compared favorably to the ionophore-mediated [^111^In]In-oxine method, particularly due to its simple and easily reproducible radiochemistry that facilitates adoption and routine use of this technique. However, work by Tsopelas et al. has observed that both ^99m^Tc-SnF_2_ colloids and ^99m^Tc-SnF_2_ colloid-labeled leukocytes showed very similar biodistributions in rats (predominantly in the liver and spleen).^204^ This similarity in distribution makes it very difficult to distinguish cellular uptake and free colloid distribution in vivo. Furthermore, the heterogeneity of the SnF_2_ radiocolloid formation coupled with the uncertainty of the mechanism of cell radiolabeling, compared with other radiolabeling methods, limit the use of these compounds.

#### 4.5.2 Nanoparticles

The clearance of nanoparticles by phagocytic cells (e.g., macrophages)^219^ makes these cells good candidates for labeling with radiolabeled nanoparticles (Figure 6 and Table 6). Internalization of nanoparticles by nonphagocytic cells can also be induced, for example using a protamine sulfate-heparin or with electroporation. Chitosan nanoparticles (CNs) have been reported for direct cell labeling. CNs were directly labeled with both ^64^Cu and ^89^Zr without the need for a chelator, and used to radiolabel human leukocytes.^205,206^ The Cu^2+^ and Zr^4+^ ions likely bind to the amine and hydroxyl groups abundantly present on the chitosan polymer. Uptake of the radiolabeled particles was proposed to occur via phagocytosis. Using the same chitosan polymer, ^89^Zr-CNs showed much higher uptake compared with the ^64^Cu-CNs—∼70% and 25%, respectively—and higher cellular retention was observed for ^89^Zr-CNs (53% after 24 h), whereas almost all activity was lost from ^64^Cu-CN-labeled leukocytes after just 3 h.

**Table 6:** Summary of the Various Types of Radiolabeled Nanoparticles Used for Direct Cell Labeling, along with the Corresponding Radionuclides and the Cell Type Labeled

cell labeling agent (nanoparticle)	radionuclide	cell type labeled	ref
chitosan nanoparticles	^64^Cu	WBCs	(205, 206)
	^89^Zr		
exosome-mimetic vesicles	^99m^Tc	WBCs	(207)
carboxymethylcellulose-based nanoparticles	^68^Ga	WBCs	(208)
gold nanoparticles	^125^I	dendritic cells	(209)
	^124^I		
	^64^Cu	CAR T-cells	(210)
protamine–heparin mixture	^89^Zr	hematopoietic progenitor cells	(211)
SPIONs	^64^Cu	CAR T-cells	(212)
	^18^F	mesenchymal stem cells	(213, 214)
	^111^In	adipose-derived stem cells (ADSCs)	(215)
silica nanoparticles	^131^I	bone marrow stromal cells (BMSCs)	(216)
	^68^Ga	breast cancer cells (MDA-MB-231)	(217)
	^89^Zr	CAR T-cells	(218)

Son et al. labeled red blood cell-derived exosome-mimetic vesicles (RBC-EMVs) with ^99m^Tc using the stannous chloride method. The vesicles were then used to radiolabel WBCs.^207^ Uptake of ^99m^Tc-RBC-EMVs was shown to be dose- and time-dependent, and the incubation times (12–18 h) required to reach maximum uptake levels in cells are too long for this method to be clinically applicable.

Carboxymethylcellulose-based nanoparticles were directly radiolabeled with ^68^Ga^3+^ and, subsequently, used to radiolabel WBCs.^208^ Labeling efficiencies of ∼16% were achieved after 45 min, with low cellular retention (52% after just 45 min) observed. These results indicate this approach may not be the most favorable for this application.

**Figure 15:**
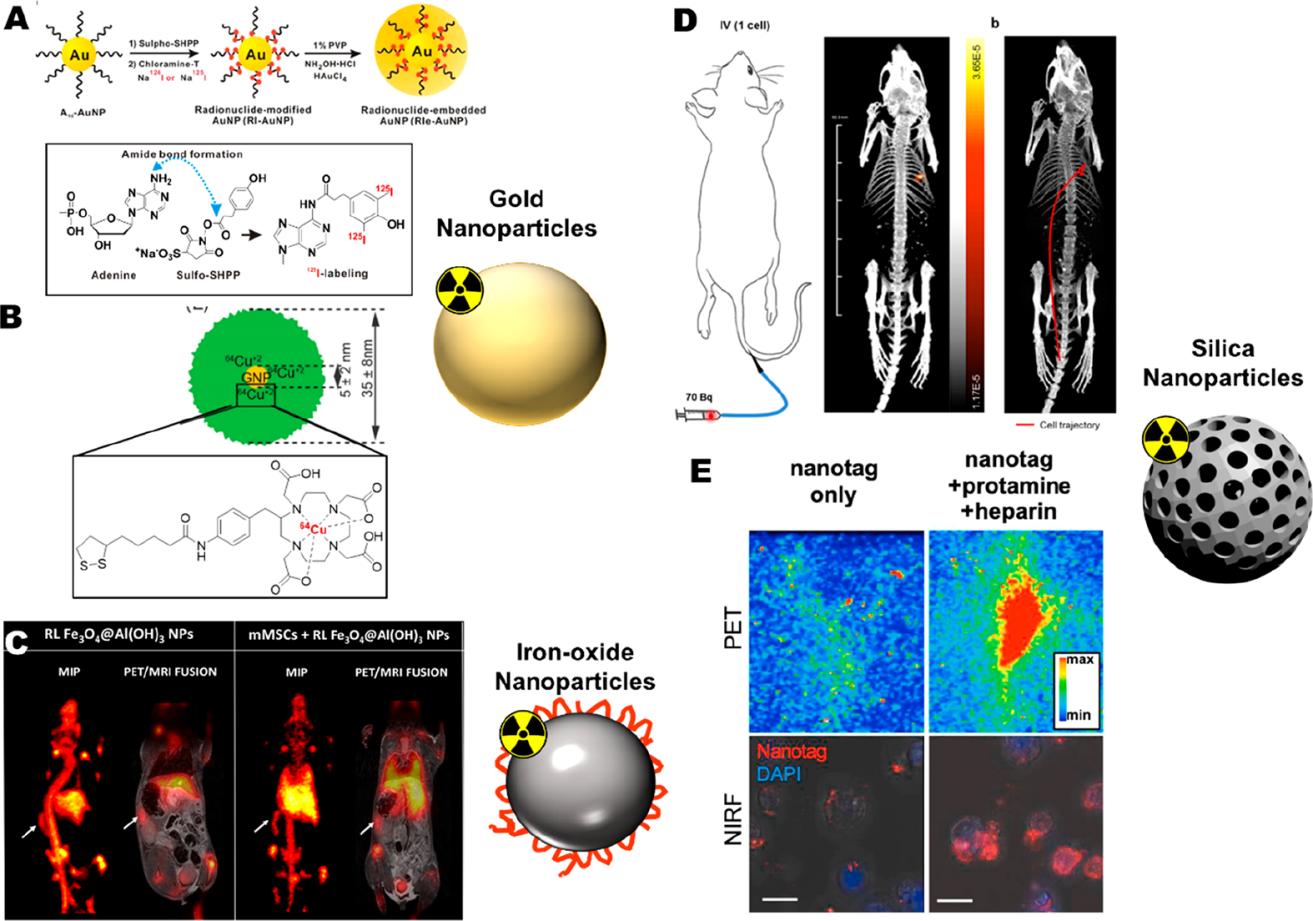
(A) Synthetic scheme and characterization of radiolabeled gold nanoparticles (RIe-AuNPs). Schematic of synthesis of RIe-AuNPs. Modification of the chemical structures of adenine, using sulpho-SHPP for conjugation of a phenol moiety, allows radiolabeling with [^125^I]NaI via aromatic electrophilic substitution. Adapted with permission from Lee et al., ref (209). Copyright 2016 Springer Nature under CC License [https://creativecommons.org/licenses/by/4.0/]. (B) Schematic showing the radiolabeling method used for gold nanoparticles used to radiolabel CAR T-cells. A DOTA-thioctic acid bioconjugate was used to bind to the gold nanoparticle surface. Adapted with permission from Bhatnagar et al., ref (210). Copyright 2012 Oxford University Press. (C) PET/MRI images of ^18^F-labeled Fe_3_O_4_@Al(OH)_3_ NPs (left) and of mMSCs labeled with ^18^F-labeled Fe_3_O_4_@Al(OH)_3_ (right). Bone uptake can be observed in the images. Adapted with permission from Belderbos et al., ref (214). Copyright 2020 Springer Nature under CC License [https://creativecommons.org/licenses/by/4.0/]. (D) A single MDA-MB-231 breast cancer cell radiolabeled with ^68^Ga-labeled silica nanoparticles (70 Bq) was injected via butterfly catheter into the tail vein and imaged using PET/CT imaging. Dynamic trajectory reconstructed from the same list-mode data tracks the single cell as it travels through the bloodstream and arrests in the lungs (right). Adapted with permission from Jung et al., ref (217). Copyright 2020 Springer Nature. (E) Panels demonstrating the endocytic uptake a combination of SiNPs nanoparticles, heparin and protamine for the direct cell labeling of CAR T-cells. Top row is PET imaging of a vial containing the cells and the silica nanotag with and without protamine and heparin. Bottom row shows fluorescence microscopy of CAR T-cells stained with DAPI for nuclei and of the NIR fluorescent SiNP nanotags; with and without protamine and heparin. It is clear protamine and heparin allows increased uptake of the nanoprobe. Adapted with permission from Harmsen et al., ref (218). Copyright 2020 Elsevier.

Lee et al. reported the radiolabeling of dendritic cells using radiolabeled oligonucleotide-modified AuNPs.^209^ The AuNPs were reacted with a water-soluble Bolton–Hunter reagent via free amines on adenine present in the oligonucleotides. This allowed radiolabeling of the AuNPs with ^125^I or ^124^I (Figure 15A). Subsequently, an additional Au shell was formed on the radiolabeled particles (Figure 15A). Cellular uptake of the AuNPs was found to be dose- and time-dependent, with the peak of ∼40% LE being reached after 3 h. The cellular retention of the AuNPs was good with ∼60% retention after 3 days with limited effect on the cell viability (>80% after 48 h) suggesting little cytotoxicity of the AuNPs. Interestingly, it was shown that the additional gold shell was necessary for high cellular retention, as radiolabeled AuNPs without the protective gold layer showed rapid removal from dendritic cells (almost all radioactivity gone within 3 h).^209^ However, the requirement for this additional shell formation step complicates the method and would likely limit its clinical utility.

Radiolabeled AuNPs have also been used for direct cell labeling of CAR T-cells. AuNPs were radiolabeled by the use of a DOTA-thioctic acid bioconjugate (Figure 15B), which allowed attaching DOTA to the gold surface and radiolabeling with ^64^Cu.^210^ The nonphagocytic cells were labeled using an electroporation process, which increases the permeability of cell membranes via pore formation by the application of an electric field.^220^ Although electroporation could be faster and enable the labeling of more cell types than endocytosis/phagocytosis mechanisms, which can take several hours for AuNPs,^221^ this process severely impacts the viability of the cells.^212^ The authors subsequently reported the labeling of CAR T-cells using ^64^Cu-labeled USPIONs.^212^ The commercial nanoparticles were preconjugated with the chelator DOTA. To avoid the use of electroporation the authors used DMSO as a membrane permeabilising agent to increase uptake of the USPIONs. It was found that 3% DMSO allowed increased uptake of nanoparticles into the CAR T-cells—with 50% LE achieved with optimized conditions—but led to a reduction in the cell viability compared to unlabeled controls.

Belderbos and collaborators reported the use of radiolabeled superparamagnetic nanoparticles consisting of a magnetite core (Fe_3_O_4_) embedded in an aluminum hydroxide shell (Fe_3_O_4_@Al(OH)_3_) for the tracking of mesenchymal stem cells.^213,214^ The aluminum hydroxide shell allows the direct adsorption of [^18^F]F^–^. One major drawback of this method is the instability of the ^18^F label on the particles, which was demonstrated in vitro in serum, and in vivo, where bone uptake was observed (Figure 15C). This was also seen for the radiolabeled MSCs, albeit at lower levels.^214^ ADSCs have also been radiolabeled using ^111^In-labeled SPIONs.^215^ After incubation with cells, histology and TEM showed the nanoparticles were taken up intracellularly and were present within the lysosomes. One drawback of this method was the need for a long cell labeling time (16 h) which may limit its clinical use. Nonetheless, cellular retention was high with ∼73% of activity remaining in the ADSCs over 7 days, with no effect on cell viability or cell function for up to 7 days.^215^

Yao et al. reported the labeling of bone marrow stromal cells (BMSCs) with cobalt protoporphyrin IX (CoPP)-loaded mesoporous silica nanoparticles (CPMSNs) with a ^125^I-conjugated/spermine-modified dextran polymer (^125^I-SD) as the shell (CPMSN@^125^I-SD),^216^ achieving 46% LE after 4 h and 60% after 8 h. Nanoparticles without the cationic coating had significantly lower uptake (15% after 8 h). The CPMSNs were found to be unstable intracellularly with the gradual release of Si and the porphyrin observed over time; the effect of this on cellular retention of the ^125^I radiolabel was not explored.

Mesoporous silica nanoparticles (MSNs) were also used for cell labeling,^217^ taking advantage of the ability or MSNs to form stable coordination complexes with oxophilic radiometals such as ^68^Ga and ^89^Zr, through deprotonated Si–O^–^ groups on their surface.^222^ The MSNs were also coated in lipofectamine to increase cellular uptake. This allowed 95% LE of MDA-MB-231 breast cancer cells for lipofectamine-coated ^68^Ga-labeled MSNs, with only 20% LE for the uncoated MSNs. However, cellular efflux of ^68^Ga using this method was high, with nearly 50% of activity released after 2 h, primarily as unchelated ^68^Ga.^217^ Larger amounts of ^89^Zr could be incorporated into cells with the MSNs, likely due to the increased oxophilicity of the Zr^4+^ ion, but with similar efflux to the ^68^Ga-MSNs. This labeling method was highly efficient and allowed the loading of a single breast cancer cell with enough activity (∼70 Bq per cell) to allow the in vivo tracking of a single cell using PET (Figure 15D) While the radiolabel stability and cell viability of this method is not optimized for long-term in vivo cell tracking for cell therapy applications, this study does highlight the beneficial cell labeling properties of lipofectamine-coated MSNs.

Harmsen et al. also used silica nanoparticles directly labeled with ^89^Zr for cell labeling.^218^ Self-assembling nanocomplexes were formed by mixing ^89^Zr-labeled SiNPs with protamine and heparin, a cell labeling strategy previously reported by Thu et al. using ferumoxytol.^223^ This heparin-protamine combination was also shown to allow cell labeling with just the addition of neutralized [^89^Zr]Zr-oxalate by Pantin et al.^211^ The authors labeled HPCs with the ^89^Zr-protamine-heparin complex. Rapid efflux was observed, with <25% retained after 14 h. Harmsen et al. demonstrated that the combination of SiNPs, heparin and protamine facilitated endocytic uptake of the nanoparticles (Figure 15E). CAR T-cell LE with the nanocomplexes was ∼83%, with both protamine and heparin necessary for high LE, however no in vitro cellular retention data was reported. No effect on cell viability was observed for up to 7 days. Notably, the ^89^Zr-labeled SiNPs were shown to remain within CAR T cells in vivo for about 1 week, after which they were progressively released into the tumor tissue that the CAR T cells had surrounded.^218^

One potential drawback to the use of nanoparticles as cell labeling agents, in particular SPIONs, is the transfer of these labels from administered cells to resident tissue macrophages.^224,225^ While this phenomenon has not been reported with cells labeled with radioactive nanoparticles, this highlights the need for ex vivo validation that the radionuclide signal maintains its association with the original cells (i.e., with FACS analysis or histology).^3^

## 5 Important Considerations for Direct Cell Radiolabeling

In this section, we will describe aspects that should be considered when radiolabeling cells, including radiolabel retention, radiolabeling conditions, dosimetry, radiotoxicity, and retention of cell functionality. While some of these considerations are also applicable to indirect cell radiolabeling or radiolabeling of molecules more generally, we will address them mainly in the context of direct cell labeling.

### 5.1 The Cell Population: What Are We Labeling?

Cells used for radiolabeling are often mixed populations of cells rather than individual cell types, particularly for radiolabeled blood cells. With mixed populations, in images obtained after injection of radiolabeled cells, a non-negligible fraction of the signal may arise from labeled cells that behave differently from the cells of interest in terms of target organs and circulation time. The main reason for using mixed cell populations is a technical limitation: until the development of automated cell sorting instruments and antibody-coated magnetic beads, the only way to separate blood cell populations was by differential centrifugation, based on differences in densities between cell types. The classical method involves centrifuging anticoagulated blood mixed with a solution of methylcellulose and hydroxypropyl methylcellulose to facilitate the sedimentation of erythrocytes, producing a supernatant containing leukocytes, platelets, and plasma proteins—all of which can be further separated by centrifugation at higher speeds. Thus, from a healthy human it is possible to obtain a leukocyte preparation containing 40–70% of granulocytes (primarily neutrophils, but also non-negligible proportions of eosinophils and basophils), with the remainder comprising of mononuclear cells (lymphocytes, monocytes, NK cells, etc.), “residual” platelets, and erythrocytes in proportions that may vary considerably in patients with infections or hematological diseases. This crude separation method remains the standard endorsed by nuclear medicine societies and allows the presence of platelets and erythrocytes in numbers similar to the total leukocyte numbers.^146,226^ The use of discontinuous density gradients can further separate granulocytes from mononuclear cells, but this technique alone does not allow more precise separation, for example of neutrophils from eosinophils or B cells from T cells. For mixed cell populations, postlabeling cell separation using beads^227^ or flow cytometry^73^ can provide a higher level of homogeneity. In theory, flow-assisted cell sorting could also measure differences in radiotracer uptake between cells from a “pure” population but in different states of activation or metabolic activity (e.g., at difference stages of the cell cycle), provided a marker can be found to identify these states. After radiolabeled cells have been administered to an animal, the digestion of target organs into single-cell suspensions followed by cell sorting and gamma-counting could also be used to assess the retention of the radionuclide within the initially labeled population.^228^

**Figure 16:**
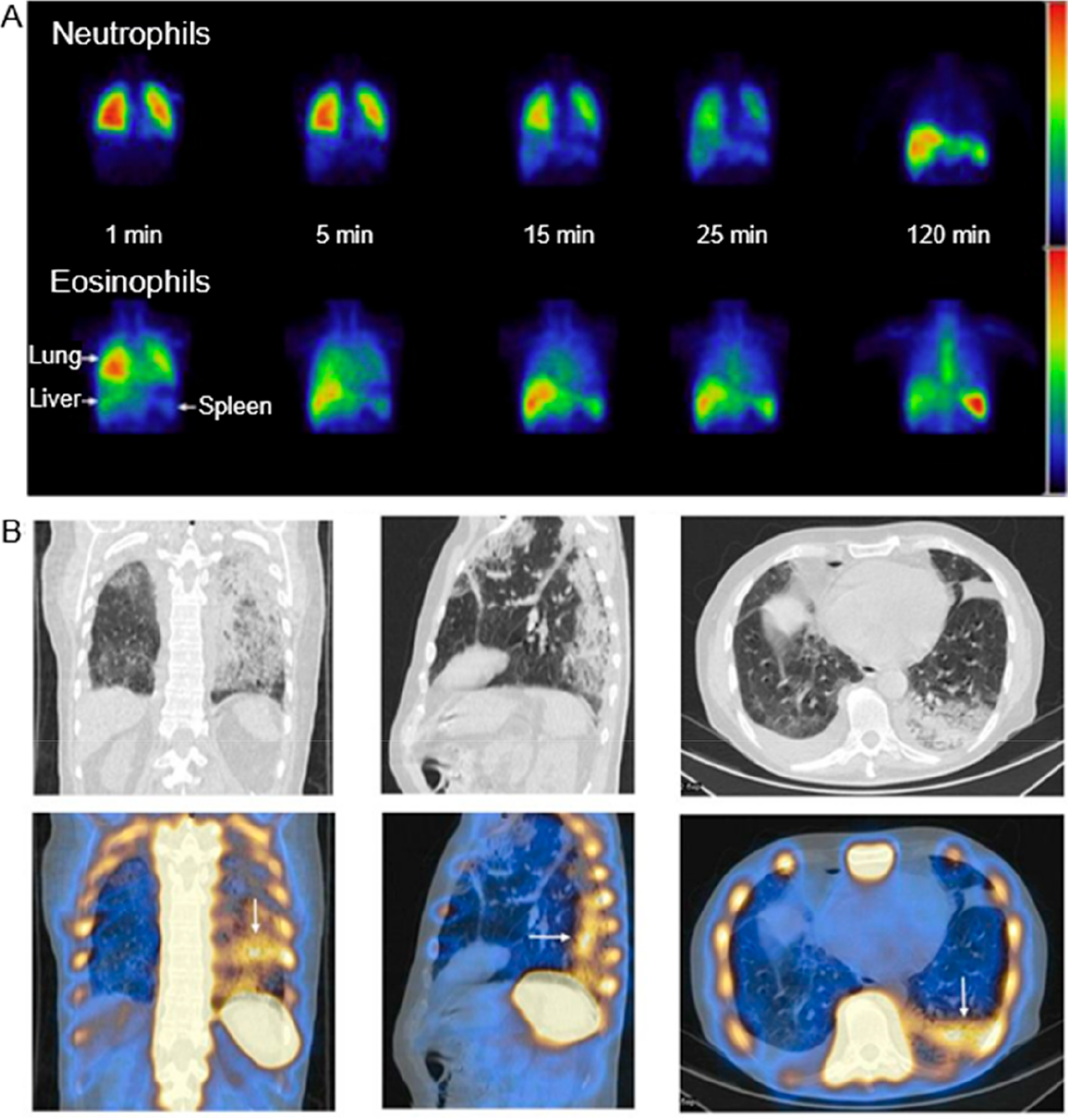
(A) Representative SPECT/CT images of affinity bead-purified neutrophils and eosinophils labeled with [^99m^Tc]Tc-HMPAO and injected intravenously to healthy volunteers. The images show the longer retention of neutrophils in the lung vasculature compared to eosinophils. Eosinophils rapidly traffic to the liver and spleen. Reproduced with permission from Lukawska et al., ref (235). Copyright 2013 Elsevier. (B) Coronal, sagittal, and transaxial CT (top) and fused SPECT/CT (bottom) images of an asthmatic patient with pulmonary eosinophilic inflammation, demonstrating focal ^99m^Tc-eosinophil uptake in areas of abnormality in the CT (white arrows). Adapted with permission from Loutsios et al., ref (241). Copyright 2015 BMJ under CC license [https://creativecommons.org/licenses/by/4.0/].

The first clinical study of radiolabeled leukocytes already acknowledged the issue of labeling mixed cell populations, noting a much higher accumulation of radioactivity in the spleen of patients injected with ^111^In-labeled cells containing large numbers of erythrocytes compared to the patient who was administered an erythrocyte-depleted preparation.^229^ The stannous pyrophosphate labeling method for ^99m^Tc suffers from the same drawback, as it efficiently labels residual erythrocytes in WBC preparations.^198^ This realization led early investigators in the field to evaluate the selectivity of various radiotracers for leukocytes over erythrocytes, although the radiotracers initially found to be the most efficient for cell labeling were not selective.^54^ [^111^In]In-tropolone was found to label preferentially granulocytes over erythrocytes.^230^ Evaluations of [^99m^Tc]Tc-HMPAO for cell labeling showed it was selective for granulocytes,^111,231,232^ but in more detailed studies, it was later found to label eosinophils 10 times more efficiently than neutrophils,^227,233^ meaning that a large fraction of the ^99m^Tc signal in a WBC scan could actually originate largely from eosinophils, despite these cells being far less abundant than neutrophils. With the use of bead-purified populations, eosinophils kinetics in humans were later characterized^234^ and found to have notably different migration patterns from neutrophils^235^ (see Figure 16). It is therefore important to properly characterize the cells that will be radiolabeled and, whenever possible, to use pure cell populations. Even within supposedly homogeneous cell populations, the distribution of the radiotracer is not always homogeneous (that is, some cells may carry much greater load of radioactivity than others that are otherwise identical).^236^ It should also be kept in mind that the labeling efficiency with a given radiotracer can vary considerably from one cell type to another, for example LE values for [^111^In]In-oxine can exceed 85% for platelets, neutrophils and lymphocytes,^97,98,122,237^ whereas for stem cells the variability is higher with reported values anywhere between 30–85%.^60,62,238−240^

### 5.2 Radiotracer Retention and the Intracellular Fate of Radionuclides

A second fundamental aspect of direct cell radiolabeling is the retention of the radiotracer/radionuclide inside or on the surface of the cells after quenching of the radiolabeling step. This is of high importance because, unlike fluorescence or bioluminescence, radioactivity cannot be switched off or selectively activated and all radiotracer signal will be acquired by the detector whether it originates within a cell or not. Consequently, it is difficult to tell a priori from a PET or SPECT image whether the signal represents live cells, damaged cells, radioactive cell debris or leaked radiotracer. To mitigate this, several approaches should be taken in conjunction. First, the radionuclide should ideally be fully retained by the cells for the useful duration of the study. This includes considering the physicochemical interactions of the radiotracer with the various cellular constituents (receptors, membrane, intracellular proteins) and its intracellular metabolism, but also ensuring that the amount of radiotracer does not result in cell damage. Second, any unincorporated radiotracer should be removed by washing the cells after incubation with the radiotracer and before further use in vitro or in vivo, to ensure that at least at the point of administration the radioactivity is fully associated with the cells of interest. Finally, for in vivo experiments, the typical distribution of the unincorporated radiotracer—and for radiometal chelates, the distribution of both the intact radiotracer and the free radiometal—should be known or established in advance. Thus, signal originating from an organ known to accumulate a certain radiotracer or radionuclide can sometimes be an indication of release from the cells. For example, unchelated ^64^Cu has high uptake in the liver; an organ in which administered radiolabeled cells will often accumulate. Hence, when using imaging ^64^Cu-radiolabeled cells, it may be difficult to distinguish signal from labeled cells localized in the liver from signal originating from released ^64^Cu from cells. A summary of the typical distribution of radiometals after intravenous administration can be found in the review by Man et al.^26^ A notable caveat is that the chemical form of the radionuclide released from the cells is rarely known.

#### 5.2.1 Impact of Labeling Conditions on Radiotracer Retention

Aside from the affinity of the radiotracer for cells, several factors can affect the labeling efficiency and retention. Although it is not within the scope of this Review to review cell separation methods, it should be kept in mind that for blood cells in particular, the separation technique can affect cell viability, metabolism, and activation state, which can in turn affect the uptake and retention of radiotracers. It is, therefore, important to ensure the isolation and labeling conditions are suitable for each cell type.

Adjuvants can be used to facilitate labeling, for example an early study showed that sodium chromate could “facilitate” the entry of ^99m^Tc into cells.^196^ Similarly, SnCl_2_ is often used with ^99m^Tc. Tin chloride reduces the technetium so that it can bind to cellular components, but the indiscriminate nature of this reaction also means that the presence of serum during labeling will reduce the labeling efficiency. Stannous pyrophosphate and stannous fluoride were also investigated but did not achieve high labeling efficiency of PMNs with ^99m^Tc.^199^ An early survey of radiotracers determined that lipophilic radiotracers generally had much higher labeling efficiencies than hydrophilic radiotracers and that labeling in plasma-free conditions was often preferable to the presence of plasma.^54^ Some metals, such as gallium and indium, form stable complexes with transferrin;^70^ therefore, incomplete removal of transferrin when isolating blood cells can reduce labeling efficiency with ^67^Ga-, ^68^Ga-, or ^111^In-based radiotracers. The use of heparin as an anticoagulant required higher concentrations of MPO or tropolone to label leukocytes with [^111^In]In-MPO or [^111^In]In-tropolone than when using citrate.^99,242^ By chelating metal ions found in plasma, citrate may reduce the amount of ions that could compete with ^111^In for binding to MPO or tropolone. While citrate is a commonly used anticoagulant, excessive amounts of citrate can chelate cell-bound radiometals, such as ^111^In, and reduce labeling efficiency.^88^ It is, therefore, important to wash cells before adding the radiotracers to remove any contaminants, either endogenous or used in the cell isolation process, that could compete with radiotracer uptake by the cells. The stability of [^111^In]In-oxine in granulocytes was shown to be low, as most of the oxine (measured by UV spectrometry) was released from the cells in the first 10–15 min of the labeling process, whereas more than 99% of the ^111^In was retained by the cells 2 h after radiolabeling. After 15 min of incubation, 80% of the ^111^In was found associated with cytosolic proteins, but after 60 min, 40% of the ^111^In was associated with nucleic material.^53^ Predictably, increasing the cell concentration during labeling resulted in higher labeling efficiencies with [^99m^Tc]Tc-oxine, [^111^In]In-oxine, [^111^In]In-MPO, [^111^In]In-tropolone, and [^18^F]FDG.^55,88,91,99,177,230^ Increasing the incubation time moderately improved the labeling efficiency of [^99m^Tc]Tc-oxine in platelets from approximately 25% to 40% over 2 h^55^ but did not affect the labeling efficiency of [^111^In]In-oxine, [^111^In]In-MPO, and [^111^In]In-tropolone in saline or Tyrode’s buffer, as the labeling efficiency reached 80–90% after only 5 min incubation.^55,243^ Labeling efficiency with [^99m^Tc]Tc-oxine was not affected by temperature, whereas labeling at 4 °C was less efficient than 25 or 37 °C for [^111^In]In-oxine, [^111^In]In-MPO, [^111^In]In-tropolone, and [^18^F]FDG.^55,98,177^ Similarly, labeling at 37 °C was more efficient than at room temperature for [^99m^Tc]Tc-SnF_2_ colloid, as expected for a radiotracer for which the uptake relies on phagocytosis.^244^ The presence of plasma proteins during the labeling step greatly reduced platelet labeling efficiency with [^99m^Tc]Tc-oxine, [^111^In]In-oxine,^55,88,243^ and to a lesser extent, in the case of [^111^In]In-tropolone,^230,243^ whereas labeling efficiency of platelets with [^111^In]In-MPO in the presence of plasma was high.^98,243^ However, increasing the neutrophil concentration to around 10^8^/mL when labeling with [^111^In]In-tropolone resulted in high (>80%) labeling efficiencies even in 90% citrated plasma.^230^

The subject of labeling in plasma or saline was investigated in many early studies. Removing plasma is undesirable because it introduces additional steps, takes longer and places cells in nonphysiological conditions. Although this clearly affects labeling efficiency, consequences for cell functionality after labeling are less clear. Isaka et al. noted that platelets labeled with [^111^In]In-tropolone in plasma had transiently higher accumulation in liver than platelets labeled in saline. However, the difference disappeared around 60 min after injection.^245^ Another study found that platelet survival was lower when labeled in saline or Tyrode’s buffer, compared to plasma, although distribution between organs 6 days after injection in rabbits was not significantly affected.^243^ It is likely that the issue of labeling in saline or in plasma is highly dependent on the cell type, as some cell types (in particular platelets and neutrophils) can very easily become activated in response to mechanical stress or changes in pH and temperature. Activation during labeling should be avoided, as activated leukocytes have longer transit times in the lung vasculature and this could potentially be mistaken for an underlying pathology.^246^

The amount of chelator can also influence labeling efficiency. For ^111^In, tropolone, oxine, and MPO concentrations of 20–400 μM were found to be optimal for platelet and leukocyte labeling.^88,90,91,99,230,245^ For [^99m^Tc]Tc-HMPAO, the concentration of HMPAO did not affect labeling efficiency.^231^ Presumably, at lower concentrations the complex is insufficiently stable in solution to label cells. At higher concentrations, the excess of ionophore could compete with cellular components for the binding of ^111^In during the labeling, reducing transchelation on which intracellular trapping depends, and [^111^In]In-tropolone may then diffuse out of the cells.^230^ Finally, if a cell labeling agent is taken up by an active mechanism (e.g., receptor or transporter), the labeling medium should not contain the natural substrate for that transporter. For example, the presence of glucose or mannose in the labeling medium reduces the uptake of [^18^F]FDG by cells due to competition for glucose transporters.^177^

#### 5.2.2 Intracellular Fate of Radionuclides

The fate of the radionuclide once inside cells affects both the retention of the radionuclide and the radiobiological effects on the labeled cells. It depends on the mechanism of entry, the chemical form in which the radionuclide is found (e.g., released or bound to the ionophore or chelator), and whether the radiotracer and radionuclide can be metabolized by the cells. In this section we discuss the case where radiolabeled cells remain viable and metabolically functional. The toxicity of radionuclides to cells is described in section 5.3, and for more detailed descriptions of the physiological roles and intracellular trafficking pathways of (radio)metals, we refer the reader to recent reviews.^247−252^

Radiotracers that enter cells through endocytic mechanisms will be found in endosomes and lysosomes, from which they may be released into the cytoplasm. Endocytic mechanisms and metabolic processes vary between cell types. Erythrocytes, for example, do not exhibit catabolic activity. Much of the knowledge in this area originates from studies of radiolabeled antibodies and metabolism of metals. For example, receptor-mediated endocytosis of ^111^In- and ^90^Y-labeled antibodies resulted in high retention of the radionuclide because of the lysosomal sequestration of radiolabeled amino acids,^253−255^ whereas with iodinated antibodies the retention of radioiodine was much lower.^254,256,257^ While the retention of radioiodine could be increased by treating cells with metabolic inhibitors,^256,258^ such treatments may also alter cell function and should be considered carefully as images obtained in these conditions may not accurately reflect the physiological behavior of cells. The cellular retention of ^124^I after internalization of ^124^I-labeled gold nanoparticles was significantly increased when the nanoparticles were protected from deiodination by an additional gold shell.^209^ Internalizing antibodies labeled with ^99m^Tc have also led to the binding of ^99m^Tc to cytosolic proteins rather than lysosomal sequestration.^259^ Several radiometals used for cell labeling have similar chemical properties to iron and, therefore, share some of its biological pathways. Most mammalian cells acquire iron through transferrin-mediated endocytosis, and manganese, indium, and gallium can also enter cells through this route.^260^ The low pH in the endosomal and lysosomal compartments causes the release of metals from transferrin. In this compartment, Fe^3+^ and Mn^3+^ must be reduced to Fe^2+^ and Mn^2+^ to be transported into the cytosol by divalent metal transporters, such as DMT1, Zip14 and TRPML1 (see review by Byrne et al.^247^). However, In^3+^ and Ga^3+^ are known not to be reduced and transported into the cytosol by similar mechanisms,^261^ and it is unclear whether or not they can escape the lysosomal compartment.

Radiolabeling agents that passively diffuse across the cell membrane, such as oxine or tropolone radiometal complexes, can bypass the endosomal route to directly reach the cytoplasm and may also enter the nucleus. From that point on, the retention depends on the existence of catabolic pathways and efflux mechanisms. While iron and zinc are exported from cells by ferroportin, gallium, copper, and manganese are not substrates of this transporter.^262^ For [^89^Zr]Zr-oxine and [^52^Mn]Mn-oxine, large differences in labeling efficiencies between cell types have been reported, with higher retention of ^89^Zr compared to ^52^Mn.^76,87^ Manganese is a cofactor for many enzymes, including arginase, glutamine synthetase, and manganese superoxide dismutase. Manganese is shuttled within cells by a number of transporter proteins and exported from cells by ferroportin and SLC30A10 (see Annagiani and Tuschl^263^). In contrast, zirconium has no biological role and few chemical similarities with other biological metals^264^ and is, therefore, more likely to remain trapped inside the cells after dissociation from its chelator. Efflux of ^89^Zr from labeled cells, through currently unknown mechanisms, is slow and is not a major impediment to imaging.^75,76,81^ Studies have shown that labeling cells with [^64^Cu]Cu-tropolone and [^64^Cu]Cu-PTSM was followed by a high efflux of ^64^Cu,^97,117^ and this could be partially prevented by adding a membrane-permeable compound that is hydrolyzed intracellularly into a chelator with high affinity for Cu^2+^, trapping the copper inside the cell.^97^ This further supports the hypothesis that biological metals (and their radioactive isotopes) that are not tightly bound to chelators when entering the cell can be used by the cell machinery. Many fundamental processes are performed by copper-dependent enzymes, such as superoxide dismutase, ceruloplasmin, cytochrome-c oxidase, and tyrosinase, based on redox cycling between Cu(I) and Cu(II).^265^ There are many copper transport mechanisms inside cells. For example, the export of copper from the lysosomes into the cytosol is thought to be mediated by an interaction between CTR1 and CTR2, and copper is loaded into secretory vesicles for cellular export by the metallochaperone ATOX1 and the copper ATPases ATP7A and ATP7B.^266^ It is likely that the low retention of ^64^Cu in cell labeling studies is due to ^64^Cu entering these export pathways.

The lipophilicity of [^99m^Tc]Tc-HMPAO enables it to cross cell membranes, after which it accumulates in organelles where it is converted into hydrophilic species, possibly by glutathione and other thiolated proteins, trapping ^99m^Tc inside the cell.^267,268^ Although the evidence for this mechanism is sparse, [^99m^Tc]Tc-HMPAO has been used as an indicator of cellular redox status, for example in the brain and in the lungs.^269,270^

#### 5.2.3 Methods to Determine the Localization of the Radiolabel Inside Cells

**Figure 17:**
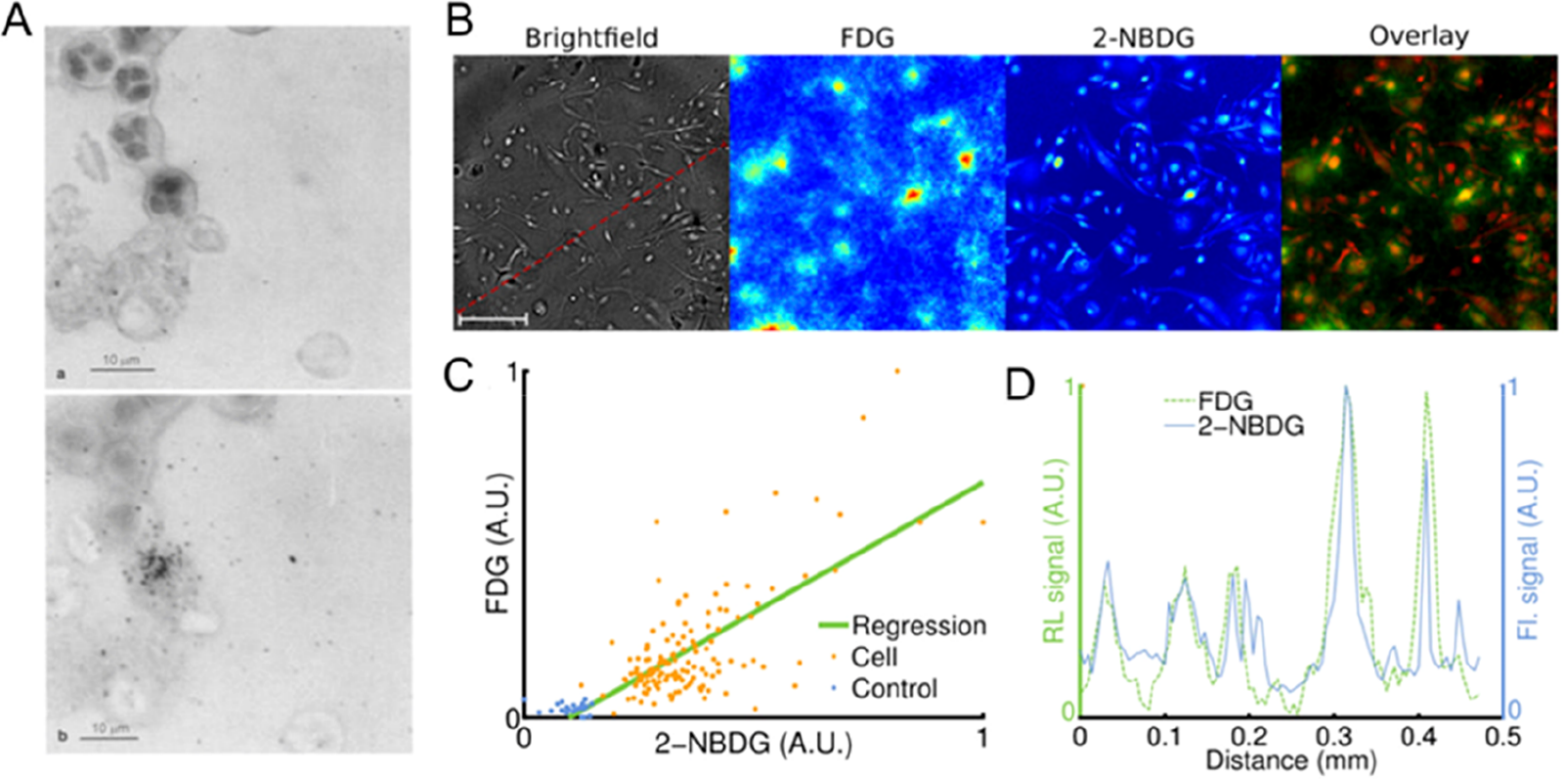
Single-cell analysis of radiotracer uptake. (A, left panels) Microautoradiograph of a typical labeled neutrophil (labeled with [^99m^Tc]Tc-SnF_2_) showing diffuse grain pattern, smear preparation, (a) cells in focus, and (b) silver grains in focus. Reproduced with permission from Puncher and Blower, ref (202). Copyright 1995 Springer Nature. (B) Radioluminescence imaging of [^18^F]FDG uptake in single cells. Human breast cancer cells (MDA-MB-231) were deprived of glucose for 1 h, incubated for 1 h with [^18^F]FDG (400 μCi) and 2-[*N*-(7-nitrobenz-2-oxa-1,3-diaxol-4-yl)amino]-2-deoxyglucose (2-NBDG, 100 μM), and then washed. (B) Brightfield (scale bar, 100 μm), radioluminescence ([^18^F]FDG), and fluorescence (2-NBDG) micrographs. Overlay, showing colocalized radioluminescence (green) and fluorescence (red). (C) Scatter plot comparing FDG and 2-NBDG uptake, computed over 140 cells (light red dots) and 26 control ROIs (blue dots). The green line was obtained by linear regression (correlation, *r* = 0.74). Arbitrary units (A.U.). (D) Radioluminescence (FDG) and fluorescence (2-NBDG) intensity profile shown along a line [red dashed line in panel B]. Reproduced with permission from Pratx et al., ref (271). Copyright 2012 PLOS under CC license [https://creativecommons.org/licenses/by/4.0/].

The most common way of measuring the activity of radiolabeled cells is to centrifuge cells and measure the resulting pellet in a dose calibrator. However, this provides only an average over the whole cell population, and no information about the distribution of activity among cells of the population or its localization on the surface or inside the cells. Intracellular localization of the radionuclide is usually determined by cell fractionation, where cells are lysed and separated into their main constituents (e.g., membrane, cytoplasmic, and nucleic fractions) by density gradient centrifugation. Another method to determine the distribution of radioactivity among a cell population or within individual cells is microautoradiography, showing for example that [^111^In]In-oxine predominantly localizes in the nucleus of leukocytes^236^ and that the colloidal radiopharmaceutical ^99m^Tc-SnF_2_ preferentially labels neutrophils because it is taken up through phagocytosis^202^ (Figure 17). With a slightly lower spatial resolution than microautoradiography but perhaps technically less involved, the recent development of radioluminescence imaging has enabled the determination of the fate of radiotracers inside living cells, with a resolution of around 20–25 μm (Figure 17).^165,217,271^ The uptake of [^18^F]FLT by actively dividing cells, explained by higher levels of thymidine kinase 1 (TK1) expression during the S-phase, could be imaged at single-cell level,^182^ and single-cell pharmacokinetic analysis of [^18^F]FDG uptake was performed.^271^ Finally, mass spectrometry imaging techniques, such as laser ablation inductively coupled mass spectrometry (LA-ICP-MS), time-of-flight secondary ion mass spectrometry (Tof-SIMS), or NanoSIMS, can be used to localize trace metals with high sensitivity and spatial resolutions below 500 nm (see reviews by Wu et al., Witt et al.^272,273^), although we have not found reports of these techniques being applied to radiometals to date.

### 5.3 Radiobiology and Toxicity

Ionizing radiation can damage biomolecules directly due to the direct deposition of low linear energy transfer (LET) radiation (e.g., photons) and high LET radiation (e.g., neutrons, Auger electrons, protons, alpha-particles, and heavy ions). With the radionuclides used for cell labeling, radiation-induced damage originates mainly from Auger electrons, positrons, and secondary electrons formed by Compton scattering of γ rays. Because of the high penetrating power of γ rays, these secondary electrons can be formed anywhere in the body, but cells closer to the source will be more affected.^274^ Radiochemical impurities, originating from daughter radionuclides or from side reactions during production, are an additional source of damage. For example, ^111^In can contain the radioactive impurity ^114m^In (*t*_1/2_ = 49.5 d) which decays to ^114^In (*t*_1/2_ = 71.9 s), which in turn emits high-energy (777 keV) β^–^ particles. Auger electrons are emitted by nuclei decaying through electron capture or internal conversion. Although the energy of Auger electrons is low (<25 keV), they have relatively high LET (1–20 keV/μm) owing to their very short-range (≤100 nm) and can, therefore, be highly damaging to cells if emitted in close proximity to radiosensitive organelles, such as nuclear DNA or the cell membrane.^275^ However, radiation-induced damage to biomolecules is predominantly indirect, occurring through the radiolysis of water. The excessive and uncontrolled formation of hydroxyl and superoxide radicals and other reactive oxygen species (ROS) and subsequently formed reactive nitrogen species (RNS), can lead to protein oxidation and nitrosylation, lipid peroxidation, and DNA damage. These result in abnormal cell signaling, perturbed enzymatic activity, genetic mutations, and cell death through increased apoptosis.^276^ For more detailed explanations of the biological and biochemical effects of ionizing radiation and how to evaluate DNA damage, we refer the reader to several reviews of the field.^277−281^ Because most of the radiation-induced damage to cells involves ROS and RNS, there is potential for antioxidants to be used as radioprotective agents. These have mostly been evaluated in the context of external X-ray irradiation and cancer radiotherapy, and we refer the reader to a recent review by Smith et al.^282^ for further details. To the best of our knowledge, radioprotective agents are not currently used in conjunction with radiolabeled cells.

The literature on radiobiology in the context of cell tracking is rather limited; studies investigating radiation-induced cell damage mostly focus on radiotherapy or environmental exposure to ionizing radiation. Overall, studies of cell radiolabeling suggest that the toxicity is primarily due to radionuclide decay rather than chemical toxicity of the ligand.^93,115,117,123,283^ The intracellular localization of the radionuclide has major implications for its toxicity. Radiation damage from ^99m^Tc and ^111^In is primarily caused by Auger electrons,^284,285^ which have a very short-range (≪1 μm) and cause damage to biomolecules in the immediate vicinity of the emitting radionuclide. Therefore, radiolabeling agents that bring the radionuclide into close proximity to the nucleus and mitochondria are more likely to cause DNA damage, as shown with [^99m^Tc]Tc-HMPAO.^268,286^ Conversely, labeling agents that remain on the cell surface or within the membrane are less likely to induce DNA damage. Unfortunately, very few studies have compared the radiotoxicity of cell labeling agents based on their subcellular location. In one example, toxicity was slower to appear in stem cells incubated with ^111^In-labeled nanoparticles than with [^111^In]In-oxine, presumably because in the former case the ^111^In was more strongly bound to the nanoparticles and therefore less available to diffuse into the nucleus or mitochondria and bind other cellular components such as DNA.^215^

Aside from radiotoxicity, some chelators and impurities have also been shown to be toxic. For example, tropolone and acetylacetone have been shown to reduce neutrophil chemotaxis and phagocytosis,^90,287,288^ whereas for oxine the effects on chemotaxis have been inconsistent between studies.^73,90,91^ One study found radiolabels such as [^111^In]In-oxine to be equally toxic to cells even after complete decay, suggesting the existence of additional cytotoxic mechanisms.^289^ When labeling with ^111^In, impurities such as Cd^2+^ can also be transported into the cells and have toxic effects.^90,287^ Differences in cell types and uncertainties around the quantity and purity of ionophores could account for discrepancies between the various studies.

In addition to the toxicity of the radiotracer to the labeled cells, the radiation dose to the rest of the body is an important factor to consider. Nonradiolabeled cells can be damaged by the emissions of nearby labeled cells, but also through biological signals (e.g., ROS, proinflammatory cytokines and other stress-associated molecules) released by radiolabeled cells.^290^ While an extensive discussion of the dosimetry of different radiolabeled cells is beyond the scope of this review, a few key points deserve mention. To date, clinical applications of radiolabeled cells have mostly involved intravenous delivery. As we describe further in this review, cells administered intravenously follow a general pattern of transient trapping in the lung circulation, typically for a few hours, followed by migration to a large extent in the liver, spleen, or bone marrow depending on the cell type. The lungs, liver, spleen, and bone marrow are, therefore, the main organs at risk from radiation delivered by radiolabeled cells.^291−293^ For red blood cells, although labeling with ^51^Cr resulted in higher RBC viability than labeling with ^99m^Tc (using SnCl_2_; 83% for ^51^Cr vs 67% for ^99m^Tc after 24 h) or [^111^In]In-oxine (94% for ^51^Cr vs 85% for ^111^In after 24 h), the high radiation dose to the spleen associated with the use of ^51^Cr and the improved imaging offered by ^99m^Tc and ^111^In were strong arguments in favor of the latter radionuclides.^294^ After ^111^In labeling, ^114m^In and ^114^In have been shown to contribute up to 10% (for radiolabeled leukocytes) and even 33% (for radiolabeled erythrocytes) of the absorbed dose to the spleen.^295^ There is much less dosimetry data available for more recent radionuclides, such as ^64^Cu or ^89^Zr in the context of cell therapies, mainly because this type of imaging has rarely been performed in patients and preclinical studies are often proof-of-concept, tracer validation studies. For ^89^Zr-labeled cells, a recent study of NK cells in rhesus macaques has suggested that administered activities up to 1.1 MBq/kg body weight would be safe in humans, which is well above the amount required to obtain good quality PET/CT images.^134^ This bodes well for human application. In this study, deferoxamine (DFO) was infused to rhesus macaques to chelate and accelerate the urinary elimination of extracellular ^89^Zr, resulting in images with better contrast and a lower radiation dose to the subjects,^134^ an approach that could easily be translated clinically. Patient safety will also greatly benefit from technological improvements, such as total-body PET scanners, as the expected 40-fold increase in sensitivity^31,32,296^ will allow significant reductions in the amount of activity used for radiolabeling.

### 5.4 Functionality of Radiolabeled Cells

Ideally, radiolabeling cells should not affect their viability and functionality. This is fundamental for a radiolabeled cell to provide an image that is representative of the biological process studied. The direct labeling of cells typically involves multiple washing steps with centrifugation and pipetting, particularly in the case of blood cells if a density gradient separation method is used. The repeated manipulation steps can reduce cell viability and functionality or lead to cellular activation, independently of the radiotracer used. It is important, therefore, to use cell isolation methods that are as gentle as possible. An illustration of this issue was given by Dewanjee et al.^88^ showing that platelet aggregability was far more affected by the isolation process than by the actual radiolabeling. A common test to evaluate the functionality of radiolabeled neutrophils after administration in patients is to measure the percentage of cell-bound activity in the blood shortly (approximately 45 min) after infusion, as cells damaged or activated during the labeling process will more rapidly accumulate in the lungs, liver, and spleen.^297^

A related question with direct practical implications is how many cells to radiolabel? For WBC labeling in the clinic, the standard practice is to isolate WBCs from 50 to 60 mL of the patient’s blood and radiolabel however many cells are obtained, as long as the ratios of RBCs and platelets to WBCs are within acceptable limits, with an amount of radiotracer (e.g., 20–37 MBq for [^111^In]In-oxine, 600–1000 MBq for [^99m^Tc]Tc-HMPAO) that is usually not adjusted for cell numbers or patient weight.^146,226^ Given the high inter- and intraindividual variability in circulating leukocyte numbers (depending for example on infection/allergy status), this results in considerable variability in terms of activity-per-cell. In some patients, for example, in neutropenia cases, it can also be difficult to obtain a sufficient number of cells, which will in turn affect labeling efficiency. For cell therapies in the clinic, the number of administered cells is better controlled and based on patient weight. To avoid damaging precious therapeutic cells, it is common to radiolabel only a fraction of the administered cells,^187,298−300^ which will have higher activity-per-cell than if the entire amount had been radiolabeled and therefore may suffer from radiation-induced damage. In a preclinical setting, this fractionated approach is not always possible because the total number of cells that can be injected safely in a small animal is significantly lower. In summary, radiolabeling of cell therapies needs to satisfy multiple independent requirements guided by very different considerations: the total number of cells to administer is determined by the biological properties of the cell therapy product and by patient safety/efficacy considerations; the total activity to use depends on the chosen radionuclide, the desired time scale for imaging, the sensitivity of the scanner and the expected number of cells at the target location. Linking these parameters is the radiobiology aspect that imposes further constraints. In other words, for in vivo cell tracking, a balance needs to be struck between image quality, toxicity to the radiolabeled cells, and whole-body dosimetry. An excessive amount of radiotracer in the cells might lead to premature cell death or loss of critical functionality, such as chemotaxis or proliferative abilities. The resulting image may offer a good contrast but may be biologically and medically irrelevant. On the contrary, cells labeled with an insufficient amount of radiotracer may retain full functionality, but this may result in count rates too low for meaningful imaging–and therefore unnecessary exposure of the subject to ionizing radiation and waste of resources. If the number of cells to be administered is large, the amount of radioactivity per cell may not adversely affect the radiolabeled cells, but the total administered dose should also remain within safe limits for the organs in which the cells will accumulate.

Not all cell types are equally affected by radiolabeling. Lymphocytes are known to be particularly sensitive to radiolabeling. For ^111^In, activities of around 5–10 kBq/10^6^ cells were found to be “safe” (i.e., survivable) for lymphocytes^113,301−304^ and hepatocytes,^57^ whereas activities higher than 20–30 kBq/10^6^ cells may be sufficient to adversely affect cell trafficking.^56,113,283,304^ For ^99m^Tc, activities of 100 kBq/10^6^ cells led to the appearance of numerous micronuclei in lymphocytes.^284^ Illustrating the difference between cell types, human embryonic stem cells labeled with [^64^Cu]Cu-PTSM remained capable of proliferating with up to 74 kBq/10^6^ cells,^305^ whereas HeLa cells proliferated unhindered with up to 185 kBq of ^111^In bound per million cells.^283^ Similarly, mesenchymal stem cells labeled with up to 140 kBq/10^6^ cells remained viable and able to produce cardiac myosin for up to 14 days.^94^ Some studies have reported that even higher activities per cell were achievable; for example, the viability and chemotaxis of endothelial progenitor cells were not affected up to 4 days after labeling with 10 MBq/10^6^ cells of [^111^In]In-oxine,^60^ whereas the same functions in hematopoietic progenitor cells were significantly affected 24–48 h after radiolabeling.^238^ However, it was also shown that the toxicity of [^111^In]In-oxine and [^89^Zr]Zr-oxine may only become apparent after 2–5 days.^77,85,306,307^ Yoon et al. showed that the proliferation of MSCs over 14 days was significantly inhibited, but not abolished, by ^111^In at 38 MBq/10^6^ cells, although this effect was not visible in the first 24 h following labeling.^93^ The toxicity of [^18^F]HFB on cardiac progenitor cells was only apparent 24 h after labeling, despite the short half-life of ^18^F.^164^ Stem cells labeled with [^18^F]FDG also showed only transient decreases in proliferation ability, which normalized 4 days after labeling.^178^ These studies illustrate how functional assays for radiolabeled cells should be performed on a time scale that is relevant both to the cell type and the radionuclide used and that simple viability assays immediately after radiolabeling are not sufficiently reliable indicators. Furthermore, nonuniform uptake by cells could also confound the reliability of viability and functional assays because cells that are more heavily labeled than the average are more likely to have damaged function and contribute disproportionately more to the signal.

Furthermore, not all cell functions are equally affected by radiolabeling. For example, chemotaxis of neutrophils radiolabeled with [^111^In]In-oxine and [^111^In]In-tropolone was more affected than phagocytosis.^90^ The motility of dendritic cells was not affected by labeling with [^111^In]In-oxine (11–74 kBq/10^6^ cells) or [^99m^Tc]Tc-HMPAO (1.85–18.5 MBq/10^6^ cells),^112^ nor was their phenotype affected by labeling with [^89^Zr]Zr-oxine (90–110 kBq/10^6^ cells).^76^ Antitumoral T-cells and stem cells radiolabeled with relatively high activities of ^89^Zr (150–300 kBq/10^6^ cells) were still capable of killing tumor cells,^75,81,85^ whereas their ability to proliferate was severely curtailed at much lower activities.^81,85^ Another study found that [^99m^Tc]Tc-HMPAO (1.5 MBq/10^6^ cells), [^111^In]In-oxine (135–180 kBq/10^6^ cells), and [^18^F]FDG (120–160 kBq/10^6^ cells) inhibited T cell proliferation without affecting their immediate viability, but at those levels of activity, the [^99m^Tc]Tc-HMPAO-labeled cells retained their cytotoxic abilities whereas the [^111^In]In-oxine- and [^18^F]FDG-labeled cells did not.^113^ The implication for lymphocytes is that short-term tracking (up to 24–48 h) that does not rely on cell proliferation can be performed with higher amounts of activity than longer-term tracking, for which it will be crucial to reduce the amount of activity per cell; total-body PET will come into its own in this situation.

To assess cell viability after radiolabeling, standard viability assays using Trypan blue or annexin V/propidium iodide or other viability markers can be employed, in combination with a light microscope, an automated cell counter or a flow cytometer. The general principle of these assays is that the membrane of healthy cells is impermeable to the dye, whereas the membrane of a dead or dying cell will allow the dye to permeate through. Thus, a simple microscopic analysis can distinguish between colorless, live cells and stained dead or dying cells. Annexin V further allows the detection of apoptotic cells as it binds to phosphatidylserine residues which are normally present on the cytoplasmic side of cell membrane but are exposed outwardly during apoptosis.

Cell functionality assays will depend on the cell type and the main function that is expected from the cell population. For neutrophils and eosinophils, chemotaxis, phagocytosis, ROS production, or granule-release assays can be used.^177,198^ More recently, measuring HMGB1, an endogenous marker of cellular damage, has been suggested.^308^ For lymphocytes, cytokine secretion (e.g., IFNγ for CD4 or CD8 T cells, IL-10 for Tregs), phenotyping and proliferation assays are typically desirable. In the case of cytotoxic T cells, a cell killing assay performed with the radiolabeled T cells against the target cells is highly recommended. It is also recommended not to limit such studies to a single stimulus. For example, proliferation of radiolabeled lymphocytes was affected differently depending on the stimulus used.^303^ For stem cells, proliferation, metabolic activity and differentiation assays can be performed.^178,307^ For platelets, aggregation and degranulation assays can be used to assess function. In clinical practice, however, priority is given to administering the labeled cells to the patient without delay and functionality and viability tests are too time-consuming to be performed for each patient. The tests are, therefore, mostly performed during the method development and validation stages, and later at regular intervals. For radiolabeled WBC, in routine use a simple visual inspection of the sample is typically performed to check for the absence of clumps that would indicate leukocyte activation.^146,226^

Finally, radiolabeling cells is itself a method of assessing their viability that has been employed for decades, using for example the uptake of tritiated amino acids.^152^ By measuring the amount of tracer taken up, the protein metabolism of cells can be evaluated. Alternatively, cells can be labeled with ^51^Cr, which is released upon cell death. This method has been used, for example, for the in vitro evaluation of T cell toxicity, where the target tumor cells were radiolabeled.^309,310^

## 6 Applications and Clinical Translation of Cell Tracking

Cell tracking is based on the unique migratory capabilities of each cell type. It is worth repeating here the importance of properly characterizing the cell population to be radiolabeled or at the very least being aware of the caveats of radiolabeling mixed cell populations. The applications of radiolabeled cells can be broadly divided into diagnostic and therapeutic categories. In diagnostic imaging, a subset of patient cells, usually select populations of circulating blood cells, are extracted, radiolabeled, and infused into the same patient to determine their trafficking dynamics as a sign of normal or abnormal physiological function. This includes for example the labeling of red blood cells to determine their rate of splenic destruction, or the use of white blood cells to localize infection sites. Therapeutic applications encompass the use of radiolabeled cells as a means of tracking the engraftment of therapeutic cells, such as stem cells for regenerative medicine or tumor-killing cells in oncology, to potentially predict therapeutic efficacy or the appearance of adverse effects. Therapeutic cells may originate from a different donor (heterologous) to the recipient or from the same donor (autologous), usually after ex vivo expansion or genetic modification. Some of the more recent studies have also combined the administration of radiolabeled cells with pharmacological interventions, for example, inhibitors of specific signaling pathways or tumor-sensitizing agents, showing how these compounds can affect cell trafficking to and from various organs. Table 7 summarizes the clinical uses of direct cell labeling and tracking to date.

**Table 7:** Examples of Clinical Applications of Direct Cell Labeling

cell type	application	radionuclide/tracer	refs
**infection and inflammation**			
WBCs	musculoskeletal infections (e.g., osteomyelitis, spondylodiscitis, prosthetic joints, cardiovascular implants)	^111^In-oxine, [^99m^Tc]Tc-HMPAO, [^18^F]FDG	numerous, see, e.g., refs (189, 311−313)
neutrophils	chronic obstructive pulmonary disease	^111^In-oxine, [^99m^Tc]Tc-HMPAO, ^111^In-tropolone	(314−318)
eosinophils	asthma	[^99m^Tc]Tc-HMPAO	(234, 241, 319)
			
**cardiovascular**			
RBCs	transfusion recovery	^51^Cr	(320)
	cardiac function	^99m^Tc	(321)
	cerebral blood volume	^99m^Tc	(322)
	detection of bleeding	^99m^Tc, [^18^F]FDG	(323−325)
platelets	deep vein thrombosis, pulmonary embolism	^111^In-oxine, [^99m^Tc]Tc-HMPAO	(326−329)
	atherosclerosis	^111^In-oxine	(330)
			
**transplantation**			
hepatocytes	liver diseases	^111^In-oxine	(331, 332)
mesenchymal stem cells	cirrhosis	^111^In-oxine	(63)
peripheral blood stem cells, bone marrow stem cells	myocardial infarction	[^18^F]FDG, ^111^In-oxine	(239, 240, 333, 334)
PBMCs	graft rejection	^99m^Tc + SnCl_2_	(335)
WBCs	graft rejection	^111^In-oxine, [^99m^Tc]Tc-HMPAO	(336)
			
**oncology**			
tumor-infiltrating lymphocytes	metastatic melanoma	^111^In-oxine	(304, 337)
purified antigen-specific T cells	metastatic melanoma, breast cancer	^111^In-oxine	(309, 310)
gammadelta (γδ) T cells	melanoma, ovarian cancer, colon cancer, adenocarcinoma, breast cancer, duodenal cancer, cervical cancer, cholangiocarcinoma	^111^In-oxine	(338)
dendritic cells	multiple myeloma, renal cell carcinoma, cervical cancer, lung cancer, myosarcoma	^111^In-oxine, [^18^F]FDG, [^64^Cu]Cu-PTSM, [^99m^Tc]Tc-HMPAO	(58, 112, 188, 339)
macrophage activated killer cells	ovarian cancer	^111^In-oxine, [^18^F]FDG	(187)

With technological improvements, the quality of information has also vastly improved. Studies performed by scintigraphy or early SPECT imaging provided only qualitative or semiquantitative evaluations of cell trafficking. Modern SPECT reconstruction algorithms and PET imaging now allow more precise quantification of cell numbers in organs, detection of very low cells numbers (around 10^4^ cells^83,94,115^) and much more accurate localization of administered cells.

### 6.1 Infection/Inflammation

Along with RBC labeling, WBC labeling for infection imaging was one of the first applications of cell tracking, starting with the clinical studies by McAfee, Segal, and Thakur.^122,229^ Neutrophils are first-responder cells, rapidly recruited from circulation to sites of infection and inflammation. The release of pathogen-associated molecular patterns (PAMPs) and damage-associated molecular patterns (DAMPs) from those sites drives the activation of the immune system. Local activation of monocytes and macrophages by PAMPs amplifies the immune response by releasing chemotactic factors that attract and guide neutrophils toward the injury or pathogen. This biological process underpins the use of radiolabeled WBC as infection imaging agents.

**Figure 18:**
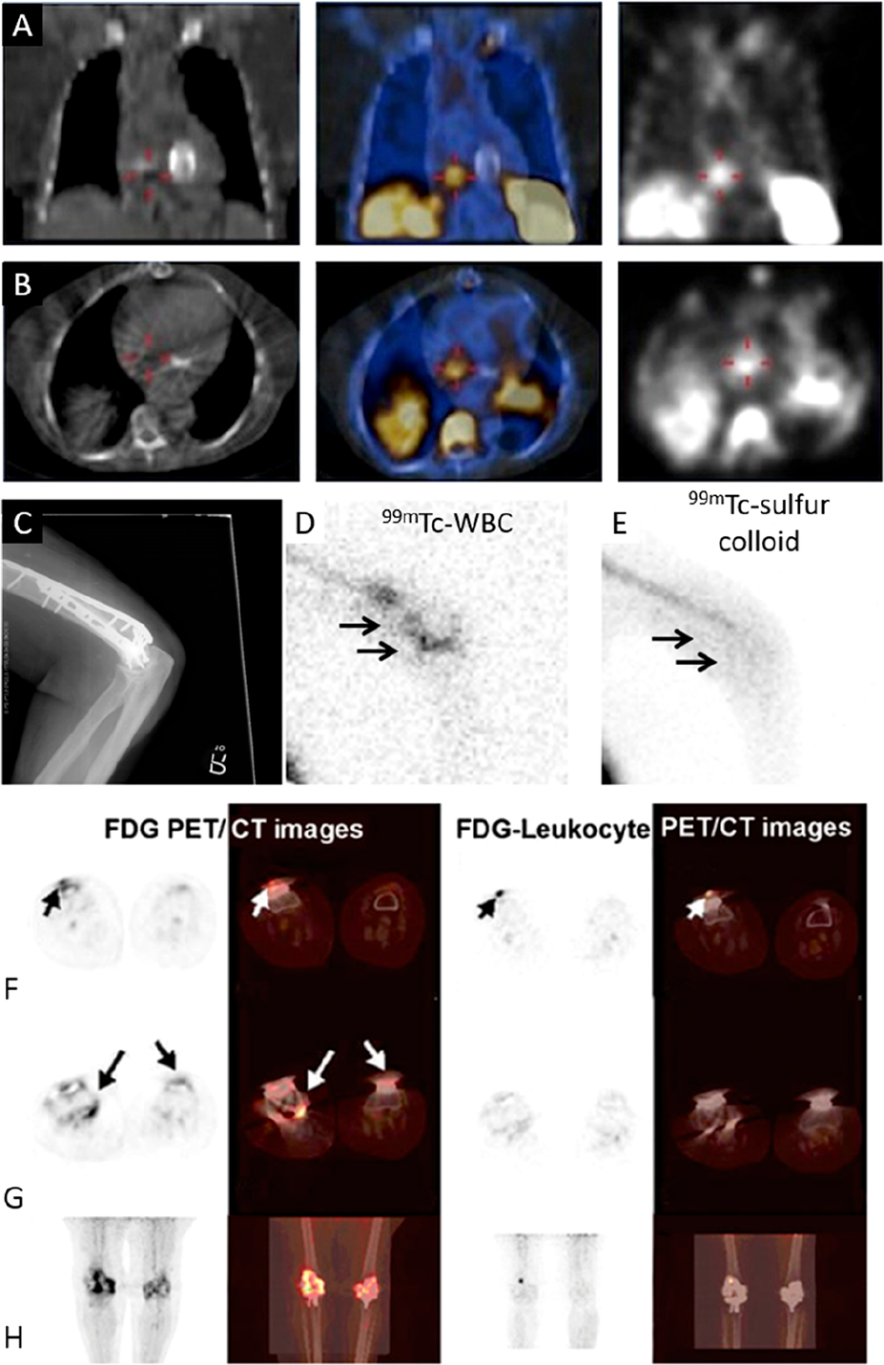
Infection and inflammation imaging with radiolabeled WBC. (A, B) [^99m^Tc]Tc-HMPAO-WBC SPECT/CT images of a patient with endocarditis of native tricuspid valve: (A) coronal views and (B) transaxial views; CT (left), fused SPECT/CT (center), and SPECT (right). SPECT/CT allowed the exclusion of an initially suspected prosthesis-associated endocarditis. Adapted with permission from Erba et al., ref (341). Copyright 2012 SNMMI. (C–E) CT and scintigraphy images taken after administration of [^99m^Tc]Tc-HMPAO-WBC (D) or ^99m^Tc-sulfur colloid (E) showing prosthetic joint infection in the distal right humerus. Note the focal accumulation of radiolabeled WBC compared to the more diffuse pattern of the colloid. Adapted with permission from Palestro, ref (311). Copyright 2016 SNMMI. (F–H) PET/CT images of a patient who had undergone bilateral knee arthroplasty 1 year previously and presenting bilateral knee pain. Selected axial (F, G) and maximum intensity projection (H) PET/CT images are shown. The [^18^F]FDG PET/CT images (two left columns) show increased [^18^F]FDG uptake around the right knee prosthesis and slightly increased [^18^F]FDG accumulation around the left knee prosthesis. [^18^F]FDG-labeled WBC PET/CT images (two right columns) show intense WBC accumulation in soft tissue in the anterior part of right knee. The final microbiological diagnosis confirmed infection of the right knee prosthesis. The clinical diagnosis confirmed aseptic loosening of left knee prosthesis. Adapted with permission from Aksoy et al., ref (351). Copyright 2014 Springer Nature.

A meta-analysis of leukocyte imaging studies to diagnose osteomyelitis of the diabetic foot found that [^99m^Tc]Tc-HMPAO-WBC scintigraphy had 91% sensitivity and 92% specificity, comparable to [^18^F]FDG-PET/CT (NB: not ^18^F-labeled WBC), whereas [^111^In]In-oxine-WBC scintigraphy had a sensitivity of 92% and a specificity of 75%.^313^ For prosthetic joint infections, sensitivity and specificity of radiolabeled leukocytes are around 85% for scintigraphy and SPECT, increasing to 90–95% with SPECT/CT.^311^ For spondylodiscitis, radiolabeled WBC have not shown satisfactory results.^340^ For cardiovascular implant-related infections, the sensitivity of [^99m^Tc]Tc-HMPAO-WBC SPECT/CT was 90–94% and the specificity close to 100%.^341,342^ Examples of these applications are shown in Figure 18. Other indications for which radiolabeled leukocytes are clinically relevant include central nervous system infections, infective endocarditis, inflammatory bowel diseases and fevers of unknown origin.^343^ Overall, [^99m^Tc]Tc-HMPAO and [^111^In]In-oxine have been found to be equivalent from a clinical perspective,^297^ and it is generally the availability of either radiolabeling agent that is the determining factor. [^99m^Tc]Tc-HMPAO has been the main agent to radiolabel leukocytes in Europe since the commercial discontinuation of [^111^In]In-oxine in 2014. A number of preclinical^177,344,345^ and clinical^346−354^ studies have investigated the use of [^18^F]FDG for labeling leukocytes, but the poor intracellular retention of ^18^F after [^18^F]FDG labeling and the additional cost compared to standard [^18^F]FDG PET/CT seems to have prevented routine use in this indication despite the wide availability of this PET radiotracer. In preclinical models, ^64^Cu has been explored as an alternative option to label WBC for PET imaging,^84,97^ but the in vitro retention of ^64^Cu was lower than ^111^In and preclinical studies showed high background signal in the abdominal region. The limited availability of ^64^Cu is also an impediment to its widespread use and clinical translation. More recently, [^89^Zr]Zr-oxine has emerged as a potential candidate for PET imaging of WBC,^73,74^ but no clinical results have been reported to date.

**Figure 19:**
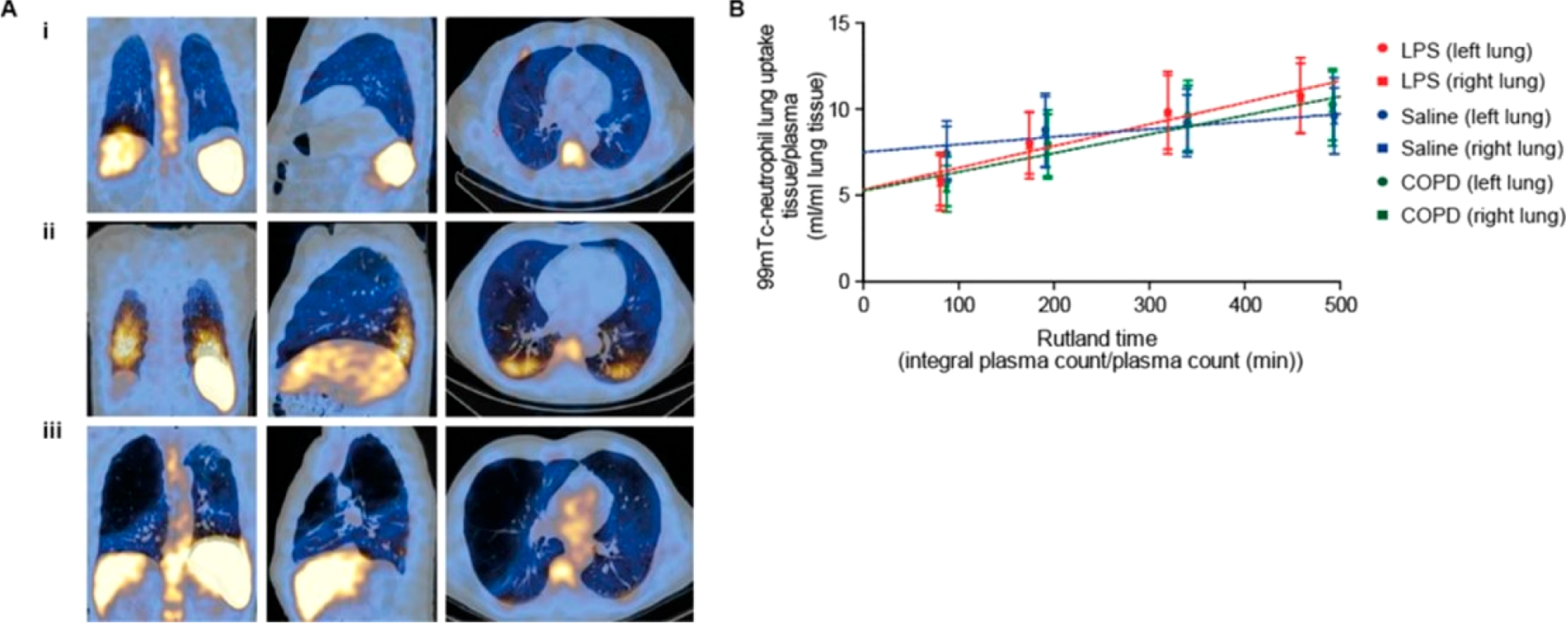
Radiolabeled neutrophils in COPD. (A) SPECT/CT images (coronal, sagittal, and transverse views, respectively) from (i) a saline-challenged healthy volunteer, (ii) an LPS-challenged healthy volunteer, and (iii) a patient with COPD. The large airspaces, with negligible radioactivity, are black and can be seen in the emphysematous lung (iii). (B) Composite Patlak–Rutland graphical plot in saline-challenged healthy volunteers, LPS-challenged healthy volunteers and patients with COPD. The plot gradient represents blood clearance of ^99m^Tc-neutrophils to the lungs in mL/min/mL lung volume. The *y*-axis intercept corresponds to the ^99m^Tc-neutrophil distribution volume. The profiles for COPD patients and LPS-treated subjects are similar to each other and markedly different from saline-treated healthy subjects. Adapted with permission from Tregay et al., ref (318). Copyright Tregay et al. 2019. Published by BMJ under CC license [https://creativecommons.org/licenses/by/4.0/].

Chronic obstructive pulmonary disease (COPD) is an inflammatory disease of the lungs primarily driven by neutrophils. Radiolabeled leukocytes have been found in higher numbers in the lung parenchyma of chronic obstructive pulmonary disease (COPD) patients compared to healthy nonsmokers,^316^ and longer transit times in the lungs of patients with acute COPD compared to stable COPD.^315^ Experimental administration of bacterial lipopolysaccharide (LPS) in healthy volunteers also resulted in increased accumulation of neutrophils compared to control individuals^318^ (see Figure 19), in line with the observation that primed (preactivated) neutrophils had longer transit times in the lungs.^314,317^ On the other hand, asthma is often characterized by eosinophilic inflammation of the lungs, which can be observed with radiolabeled purified eosinophils (see Figure 16).^241^ In asthmatic patients, eosinophil clearance from the lungs was delayed in subjects challenged with an allergen compared to nonchallenged subjects and subjects treated with inhaled corticosteroids prior to challenge.^319^ Eosinophil uptake in the lungs was also increased in obese asthmatic patients compared to nonobese asthmatic subjects.^355^ Such studies suggest that nuclear imaging of neutrophils and eosinophils could be a useful, noninvasive way of monitoring the effects of novel treatments for COPD and asthma. Radiolabeled platelets have also been used to show the recruitment of platelets into the lung airspaces in acute lung inflammation in mice.^356^

### 6.2 Cardiovascular Function

**Figure 20:**
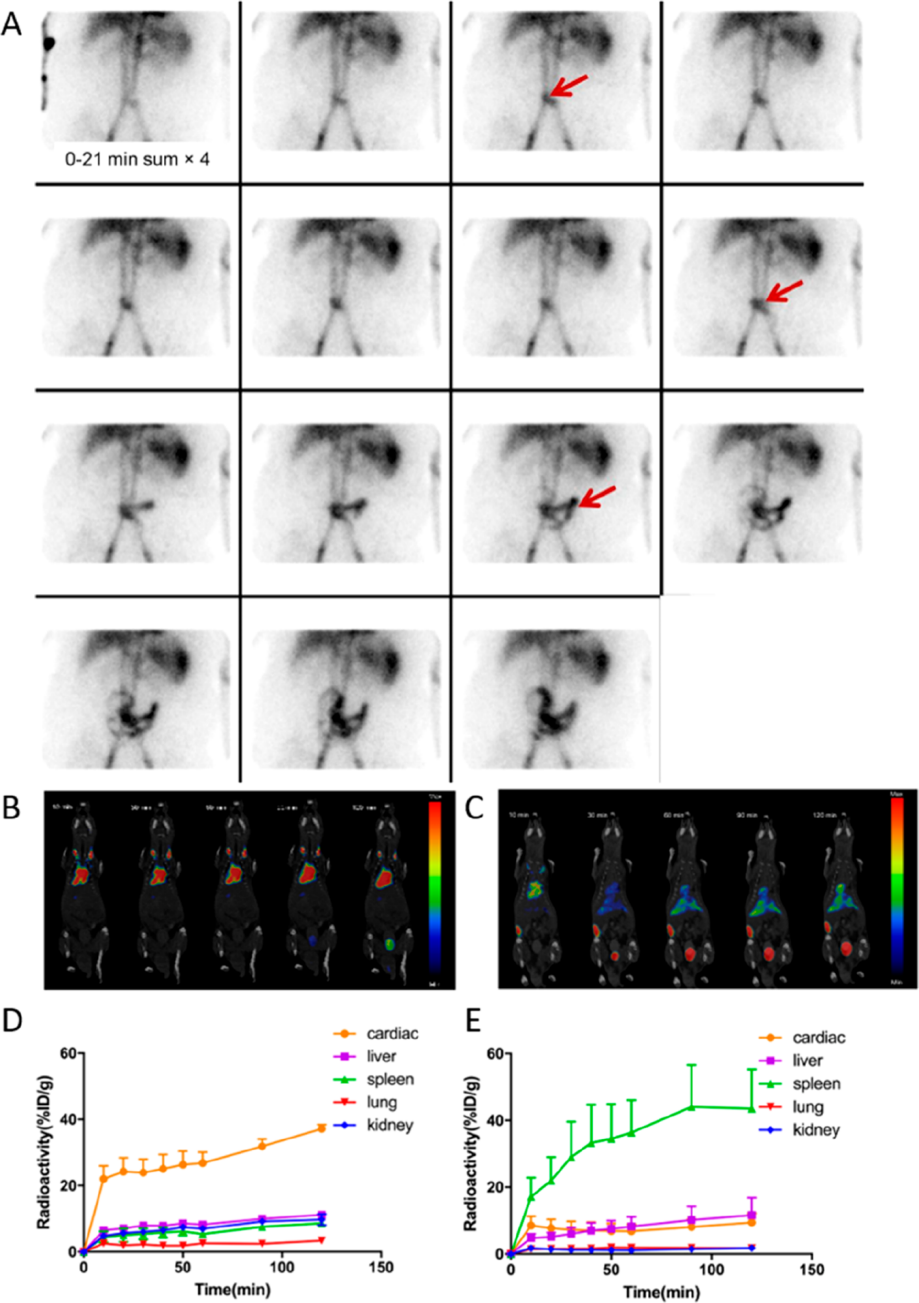
Applications of radiolabeled RBCs. (A) Scintigraphic images of ^99m^Tc-labeled RBCs showing bleeding originating from a branch of the superior mesenteric artery. A focus of increasing intensity is visible in the lower abdomen at the midline (arrows), showing anterograde and retrograde movement conforming to the bowel lumen. The focus crosses the midline several times and is therefore most compatible with small-bowel bleed. Adapted with permission from Grady. ref (325). Copyright 2016 SNMMI. (B, C) Coronal PET/CT images of untreated (B) and heat-stressed (C) [^18^F]FDG-labeled RBCs in mice, taken over 120 min. Untreated RBCs are mostly visible in the heart and carotid regions, with limited urinary excretion of [^18^F]FDG. In contrast, heat-stressed RBCs rapidly accumulate in the liver and spleen and increased release of ^18^F from the RBC is visible from the bladder signal. (D, E) Time-activity curves of ^18^F uptake in major organs after administration of untreated (D) and heat-stressed (E) [^18^F]FDG-labeled RBCs, mirroring the profiles observed on the PET/CT scans. Adapted with permission from Yin et al., ref (360). Copyright 2021 Springer Nature under CC license [https://creativecommons.org/licenses/by/4.0/].

The labeling of red blood cells (RBCs) with ^51^Cr was one of the earliest applications of direct cell labeling and has been the gold standard method for measuring transfusion recovery for nearly 50 years.^320^^51^Cr is not suitable for imaging, and imaging-compatible alternatives to this method include the use of ^99m^Tc (for in vivo labeling of RBCs with stannous chloride) or ^111^In-labeled RBCs. The lower retention of ^99m^Tc is a source of error in these measurements and thus would favor the use of ^111^In,^111,197^ but the wider availability of ^99m^Tc and the overall simpler procedure of in vivo RBC labeling has made the latter the more common approach. Radiolabeled RBCs allow blood pool imaging, which is a useful technique to evaluate cardiac function,^321^ measure regional blood volume in the brain,^322^ and detect hemangiomas^323,324^ and gastrointestinal bleeding^325^ (Figure 20A), although it has progressively been replaced in some of these roles by nonradioactive techniques such as Doppler ultrasonography or MRI. Heat-damaged RBCs are also used for spleen imaging (Figure 20B-E). [^68^Ga]Ga-oxine was recently evaluated in the clinic for the labeling of heat-denatured RBC, helping to identify a benign splenic nodule that could otherwise have been mistaken for a metastatic lesion.^69^

A few preclinical studies have recently explored the labeling of RBCs with [^18^F]FDG. RBCs are well suited for [^18^F]FDG labeling as they have high expression of the GLUT1 transporter. Overall, preclinical studies have shown that [^18^F]FDG has good uptake in RBCs compared to other cell types and encouraging imaging performance for use in blood pool imaging.^357−360^ One study showed the possibility of performing in vivo ^18^F-labeling of RBCs using 4-(2-[^18^F]fluoroethoxy)benzenesulfonamide, with good agreement between PET and MRI measurements of heart function,^184^ but no further development appears to have taken place. Surface-labeling of RBCs with ^18^F has been used to detect small areas of intracranial hemorrhage.^153^ Other candidates for PET imaging of RBCs include oxine complexes of ^64^Cu and ^89^Zr.^84,361^

Radiolabeled platelets have previously been used clinically for thrombus imaging, for example to detect deep vein thrombosis or pulmonary embolism, again mostly with [^111^In]In-oxine, [^111^In]In-tropolone, and [^99m^Tc]Tc-HMPAO.^326−329,362^ However, the relatively slow accumulation of platelets at the target site and interference by anticoagulant agents^363^ limit the utility of the procedure. Radiolabeling platelets is, as for erythrocytes, also a method to evaluate their recovery and survival after transfusion.^364^

[^111^In]In-oxine-labeled monocytes have been used preclinically to investigate atherosclerosis, showing that the specific accumulation of monocytes in large atherosclerotic lesions in the aortas of apolipoprotein E-deficient mice, best imaged 5 days after administration, was reduced after treatment with statins.^66^ It is often highlighted that nuclear medicine techniques have the advantage of using very small amounts of tracer and thus minimize the risk of disturbing the observed system. In this case, however, the number of radiolabeled cells administered exceeded the number of constitutively circulating monocytes, effectively pushing the system outside of physiological conditions.

### 6.3 Auto-Immune Diseases, Transplantations, and Stem Cell Grafts

Imaging the engraftment of stem cells has been a major field of application of direct cell radiolabeling. The variable success rate of stem cell therapies in clinical trials has been rationalized by unknown factors such as the degree of engraftment of administered cells. However, only imaging can localize and quantify this. Therefore, determining how many cells actually remain and proliferate in the target organ could potentially predict the success of the intervention in patients.^365^

Allogeneic hepatocyte transplantation is an alternative to orthotopic liver transplantation for severe liver diseases, but evaluation of cell engraftment after transplantation is challenging.^366^ In the clinic, hepatocytes administered through the portal vein remained in the liver for at least 1–5 days.^331,332^ In contrast, intravenously administered mesenchymal stem cells transited through the lungs before reaching the liver and to a larger extent the spleen, although advanced cirrhosis accompanied by splenomegaly in patients may have skewed the distribution toward the spleen.^63^ Preclinically, microautoradiography and scintigraphy were used to show that intrasplenically transplanted [^111^In]In-oxine-labeled hepatocytes translocated from the vascular spaces of the spleen to hepatic veins.^57^

**Figure 21:**
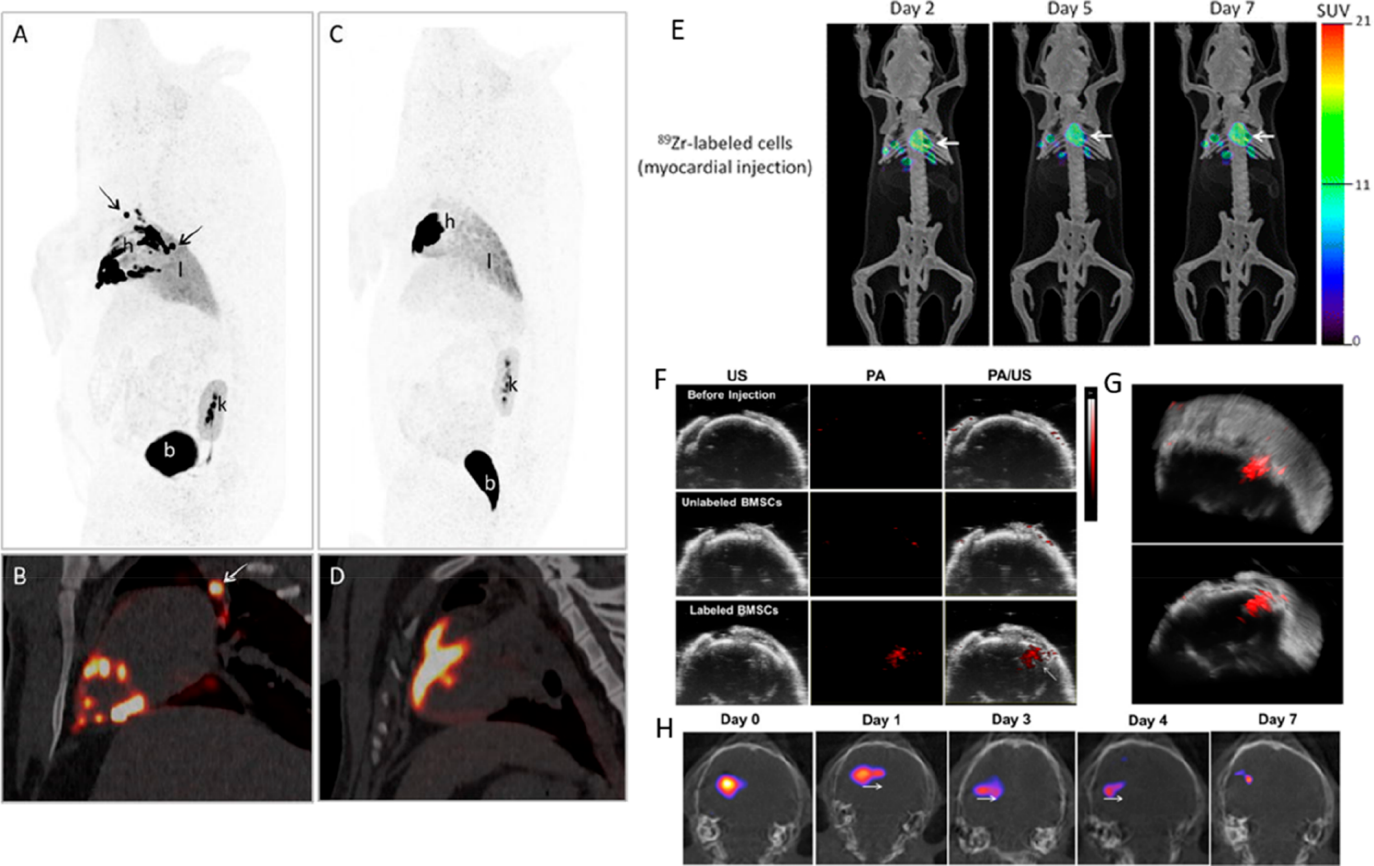
Radiolabeling of stem cells. (A–D) PET/CT images of pigs with myocardial ischemia-reperfusion injury, after intramyocardial (A, B) or intracoronary (C, D) administration of [^18^F]FDG-labeled cardiac stem cells (CSC). A, C: Whole-body maximal intensity projection images. B, D: Sagittal sections of the heart area. In intramyocardial images, a spot-pattern uptake can be clearly observed over the myocardial wall (h), whereas intracoronary administration showed a diffuse uptake. [^18^F]FDG activity could also be clearly detected in bladder (b), kidneys (k), and lungs (l). Arrows point to lymph nodes with high [^18^F]FDG uptake. Adapted with permission from Collantes et al., ref (179). Copyright 2017 Springer Nature under CC license [https://creativecommons.org/licenses/by/4.0/]. (E) PET/CT images of ^89^Zr-labeled mesenchymal stem cells following myocardial administration in an ischemia/reperfusion mouse model, showing persistence of MSCs in the heart region for up to 7 days. Adapted with permission from Bansal et al., ref (154). Copyright 2015 Springer Nature under CC license [https://creativecommons.org/licenses/by/4.0/]. (F–H) In vivo photoacoustic (PA) and SPECT/CT images of bone marrow-derived stem cells (BMSCs) tagged with cobalt protoporphyrin IX (CoPP)-loaded mesoporous silica nanoparticle (CPMSN) radiolabeled with ^125^I (CPMSN@^125^I-SD) and injected in ischemic mouse brains. (F) PA images (680 nm) of ischemic mouse brains immediately after intracerebral injection of 500 000 unlabeled or CPMSN@^125^I-SD-labeled BMSCs. (G) Representative 3D-reconstructed PA images of ischemic mouse brain tissue after injection of 500 000 labeled BMSCs. (H) SPECT/CT images of ischemic mouse brain tissue 0–7 days after intracerebral injection of labeled BMSCs. The white arrows show the migration direction of the labeled BMSCs. Adapted with permission from Yao et al., ref (216). Copyright 2020 American Chemical Society.

Several studies have used cells labeled with ^18^F, ^64^Cu, ^124^I, ^111^In, or ^99m^Tc, for example endothelial progenitor cells, hematopoietic progenitor cells and mesenchymal stem cells, in animal models of myocardial infarction,^60−62,65,92,94,115,164,166,167,238,367−369^ as well as patients.^239,240,333,334^ While the majority of the cells accumulated transiently in the lungs, then in the liver and spleen, engraftment in the heart was usually observed after intracoronary, intraventricular, or intramyocardial delivery (Figure 21).^154,164,166,167,179,181,238−240,333,334,368,369^ In contrast, stem cell engraftment in infarcted tissue after intravenous delivery has been more variable. Some reported little or no accumulation in the heart,^61,154,368^ whereas others did observe engraftment in the heart after intravenous delivery.^60,62^ The results appear to differ depending on the species, the exact type of cell, the amount of activity used for labeling and the chelator used in the radiotracer. Short-term distribution of stem cells appears to depend mainly on the injection route, as demonstrated in a recent comparison of [^18^F]FDG-labeled stem cells in mice, rats, rabbits, and nonhuman primates.^181^ Unsurprisingly, the hypoxic environment of infarcted tissue is not favorable to cell engraftment, as shown by the much shorter persistence of radiolabeled cells compared to healthy tissue.^166^

Additionally, the number of engrafted cells was low even in the more successful studies, in some cases detecting as few as 10^4^ cells.^62,83,115^ This type of information could only be obtained through imaging, further highlighting the advantages of quantitative and highly sensitive nuclear imaging methods over MRI. The relative tolerance of MSCs to high radiolabeling activities^61,92,94^ is an additional benefit, as small numbers of cells can easily be visualized, and the total administered dose to the patients can remain low. The more recent preclinical studies using PET have shown not only the degree of uptake of stem cells but their distribution within the target organ;^179^ some have used additional reporting modalities to evaluate their viability.^85^ Differentiated kidney lineage cells labeled with [^64^Cu]Cu-PTSM and implanted in fetal monkeys were observed to remain at the site of injection for up to 3 days.^305^ There was significant loss of signal on the third day, presumably due to a loss of cell viability, but it is unclear whether this decline was caused by the radiolabeling. It is expected that the use of longer-lived PET radionuclides, such as ^89^Zr, will allow such studies to extend several days or weeks after administration. One of the longest imaging studies to date showed that following intravenous administration, ^89^Zr-labeled endothelial progenitor cells accumulated significantly more in the lungs of rats with pulmonary arterial hypertension compared to control rats for over 10 days, and that this occurred after the initial lung sequestration of cells had subsided.^86^ One strategy to promote the survival of stem cells implanted in ischemic sites is to protect them from oxidative stress. For example, Yao et al. labeled stem cells with silica nanoparticles loaded with cobalt protoporphyrin IX as an antioxidant agent. The nanoparticles were additionally labeled with ^125^I, allowing the tracking of stem cells in ischemic mouse brains over 7 days and revealing their migration toward the ischemic areas.^216^ In this case, the cobalt protoporphyrin also served as a photoacoustic imaging agent (Figure 21F−H).

**Figure 22:**
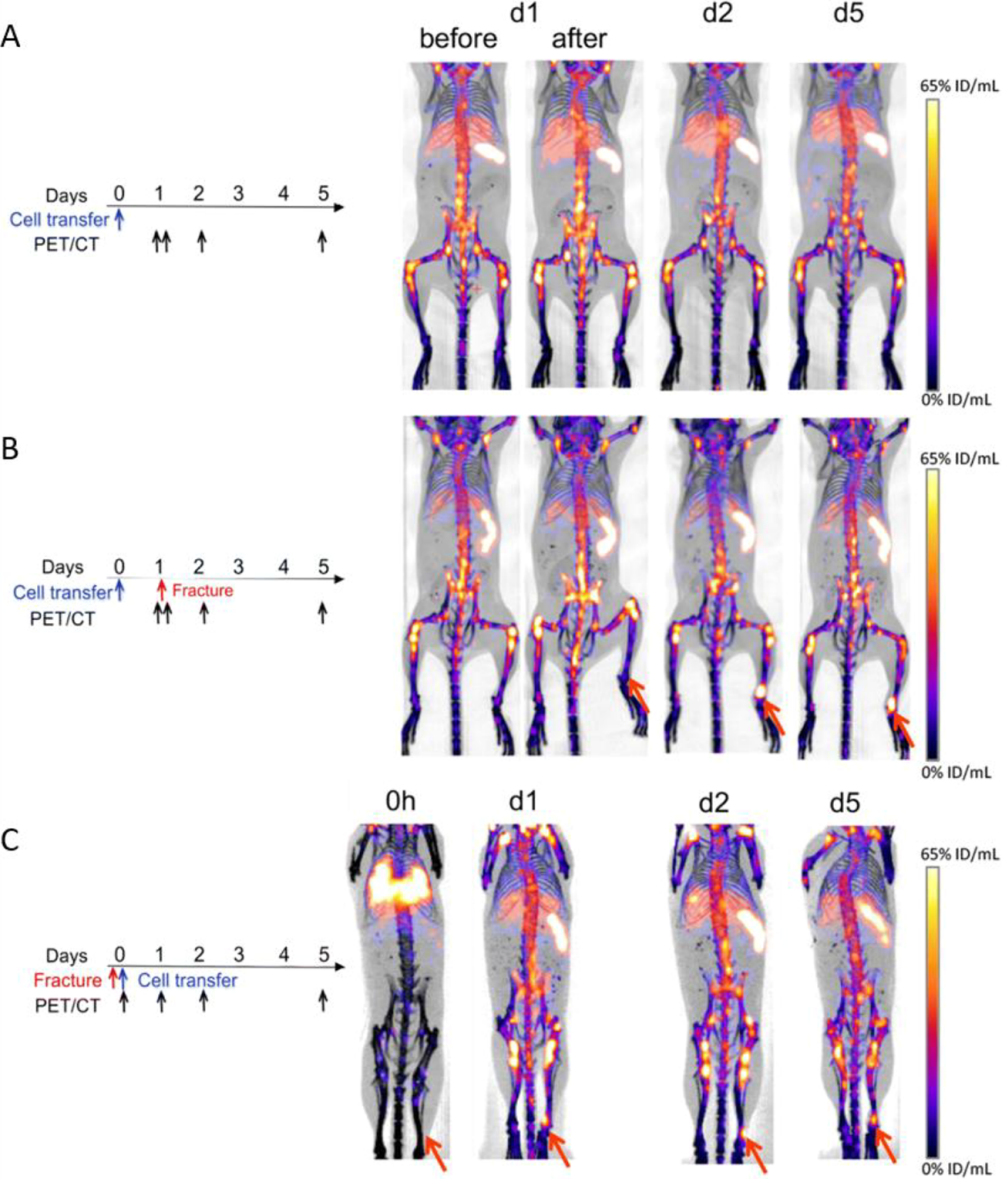
Radiolabeled bone marrow cells in bone fracture models. Longitudinal PET/CT imaging of ^89^Zr-labeled bone marrow cells administered intravenously in control mice (A), injected 1 day before tibial fracture (B), or injected on the same day as the fracture (C). In both fracture models, bone marrow cells can be seen accumulating at the fracture site (orange arrows) within 1 day of administration. In model B, the accumulation of labeled cells at the fracture site represents remobilization of administered bone marrow from organs they had initially trafficked to. Adapted with permission from Asiedu et al., ref (78). Copyright 2018 Asiedu et al. Published by Springer Nature under CC license [https://creativecommons.org/licenses/by/4.0/].

Hematopoietic progenitor and stem cells have also been used in bone marrow transplantation and bone fracture models (Figure 22), where PET imaging showed that pharmacological modulation of the CXCR4 signaling pathway could affect the homing of intravenously administered ^89^Zr-labeled cells to the bone marrow.^77,78^ These studies further demonstrate that radiolabeling of cells is a powerful technique to study the impact of pharmacological interventions on cell trafficking between organs and would merit more frequent usage.

Aside from the persistence of cells at the site of engraftment, PET imaging has also been used to optimize the injection technique. Image-guided surgical placement of catheters is usually done with ultrasound imaging or MRI. One study used PET and ^89^Zr-labeled hematopoietic progenitor cells to demonstrate that the standard intrabone delivery performed by hand via two distinct injection sites led to leakage of cells from the first site during the second injection, evidenced by diffuse activity surrounding the initial injection site and in the lungs, which did not occur after a single injection with a precisely controlled infusion rate.^211^ However, a follow-up study in rhesus macaques showed that even this optimized intrabone delivery of hematopoietic progenitor cells was less beneficial than the much simpler intravenous administration.^79^ Bone-marrow derived MSCs were also imaged in the brains of rats with traumatic brain injury.^93^ Finally, radiolabeled leukocytes have been used preclinically to evaluate graft rejection as an alternative to biopsies,^335,336,370−372^ showing for example that ^18^F-labeled lymphocytes could distinguish between allograft rejection and other causes of organ-specific toxicity.^372^

The success of stem cell therapies depends on their long-term engraftment. This is a major limitation of direct cell labeling, as cells cannot be relabeled after administration. Cell tracking after direct labeling is therefore limited by the half-life of the radionuclide and will only inform on early engraftment, particularly if ^18^F is used. Indeed, in several studies differences in engraftment at later time points were revealed by histological methods.^78,179,215^ For longer-term, noninvasive tracking, reporter gene imaging strategies or stem cell-specific tracers that can be administered repeatedly, such as antibodies, should be investigated.

### 6.4 Cancer Immunotherapies

**Figure 23:**
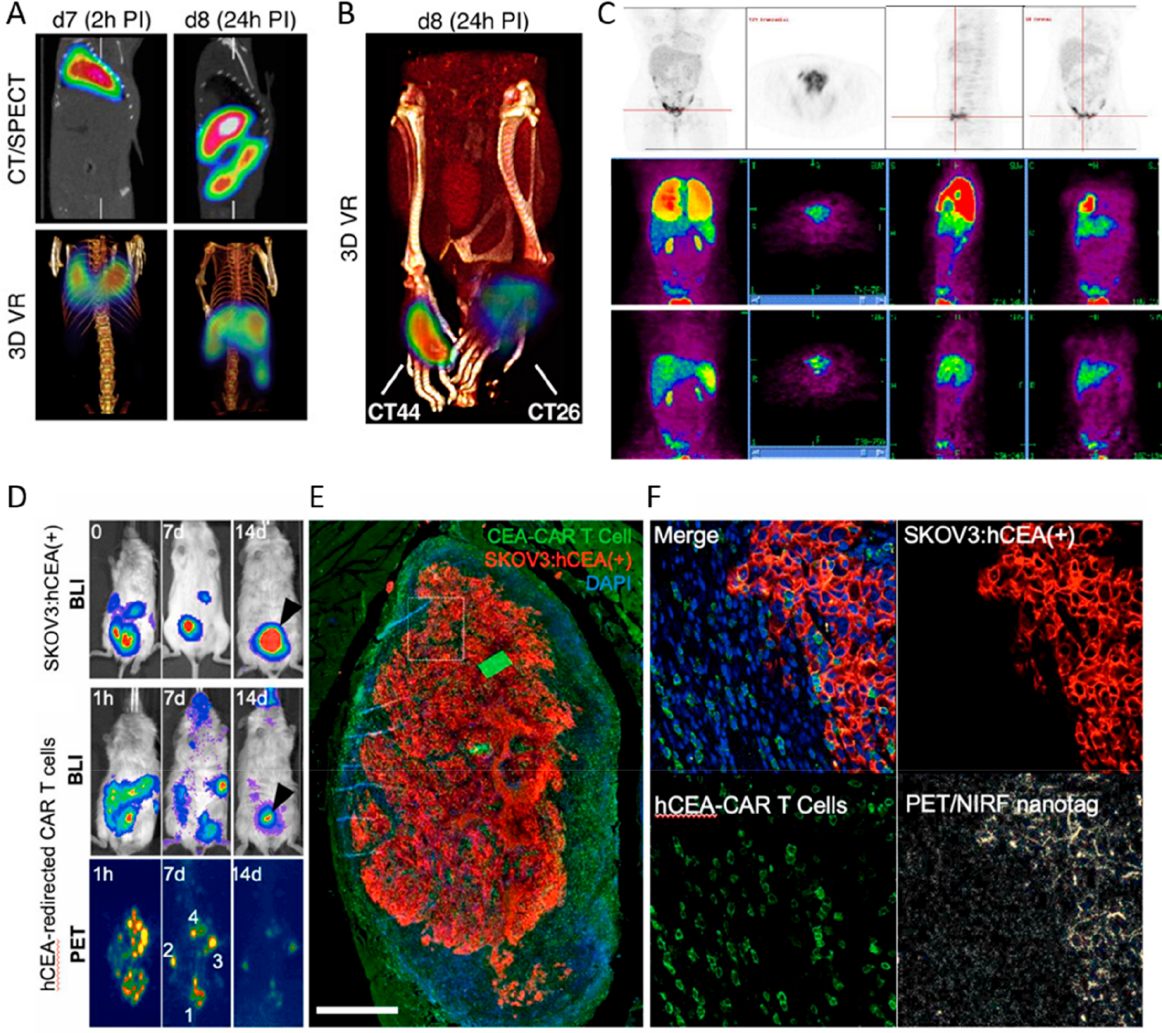
Examples of radiolabeled antitumoral immune cells. (A, B) SPECT/CT images (coronal and 3D virtual renderings) of ^111^In-labeled HA-specific cytotoxic T cells administered intravenously in mice bearing CT44 (HA-positive) and CT26 (HA-negative) tumors. In panel B, a stronger PET signal, representing higher T cell accumulation, is visible in the HA-positive tumors. Adapted with permission from Pittet et al., ref (64). Copyright 2007 National Academy of Sciences. (C) Top: Baseline [^18^F]FDG PET images of a patient, showing multifocal peritoneal metastases predominantly in the pelvis and additional lesions in the serosal peritoneum over the liver and anterior superior tip of the spleen. From left to right: Whole body, axial, sagittal, and coronal images. Middle and bottom: PET images 1 and 4 h after i.v. injection of macrophage activated killer (MAK) cells labeled with [^18^F]FDG + MDX-H210 antibody, showing accumulation of cells in the lungs (at 1 h), liver, spleen and pelvic tumor. Adapted with permission from Ritchie et al., ref.^187^ (Copyright 2007 Springer Nature). (D-F) CAR-T cell imaging in a mouse xenograft model of ovarian cancer. (D) Top: bioluminescence imaging (BLI) of SKOV3:hCEA(+) cells in an NSG mouse prior to (*t* = 0) and post adoptive T cell transfer (*t* = 7, 14 days). At *t* = 14 days post adoptive cell transfer, one major lesion was present (arrowhead); middle and bottom: BLI and PET imaging at 1 h, 14 days post adoptive cell transfer (intraperitoneal) administration of hCEA-redirected CAR T cells tagged with ^89^Zr-labeled and near-infrared fluorescent (NIRF) silica nanoparticles. (E) Immunofluorescence image of the remaining tumor (red) demonstrating that the majority of CAR T cells (green) were found most prominently in the tumor periphery (scale bar, 1000 μm). (F) In another section (box) of the tumor, it was found that at *t* = 14 days (p.i.) the PET/NIRF nanoparticles (yellow) were no longer associated with the hCEA-redirected CAR T cells but have been released and, subsequently, taken up by the SKOV3:hCEA(+) cancer cells (scale bars: 100 μm). Adapted with permission from Harmsen et al., ref (218). Copyright 2021 Elsevier.

Recent developments in cell therapies in the field of oncology, and particularly the emergence and recent clinical approvals of CAR T cell therapies, have led to an increased interest in the use of nuclear imaging to track such cells in the past decade. This recent surge, however, builds on work done over more than 40 years. Before the advent of genetically engineered cells, tumor-infiltrating lymphocytes (TILs) and lymphokine-activated killer (LAK) cells were considered promising therapies, and it is now established that the immunological profile of TILs, i.e. the relative proportions of infiltrating cell subpopulations (e.g., CD8^+^, CD4^+^, γδ cells, Tregs, B cells, NK cells) affects the clinical outcome.^373,374^ Therefore, tools to assess whether adoptively transferred cells reach their target are required. Clinical studies using ^111^In-labeled TILs or DC-stimulated tumor antigen-specific T cells in melanoma patients showed that administered cells accumulate in the lungs, liver and spleen in the first 24 h after infusion. Cells trapped in the lungs were then mostly released into the circulation and accumulated in tumors over the following days.^304,309,337^ While the uptake of lymphocytes in tumors is dependent on the presence of their cognate antigens on tumor cells, the pattern of transient trapping in the lungs and durable uptake in the liver and spleen is commonly observed in clinical studies using intravenously administered radiolabeled lymphocytes.^237,301,309,310,338,375−379^^111^In-labeled γδ-T cells were observed to accumulate in tumors in patients a few hours after administration, although patient numbers were too limited to draw further conclusions.^338^ Bernhard et al. show images from a patient in which ^111^In-labeled HER2-specific T cells were unable to penetrate liver metastases because of the barrier of stromal cells surrounding the tumor.^310^ In the case of macrophage activated killer (MAK) cells, uptake of ^111^In and ^18^F-labeled MAKs at the tumor site was observed in approximately half of the patients, after either intravenous or intraperitoneal administration^187^ (Figure 23).

The same pattern of lung trapping followed by high uptake in the liver and spleen was observed in preclinical studies.^75,81,114,117,134^ It was established using fluorescence 2-photon microscopy that activated T cells are larger and more elongated than naïve cells, and their size slows them down as they pass through pulmonary capillaries.^380^ Therefore, longer persistence in the lungs observed by PET or SPECT imaging may be an indication of early T cell activation. In mice, homing of T cells to secondary lymphoid organs (lymph nodes) has also been observed, independently of the specificity of the T cells.^96^ In contrast, radiolabeled cells administered intraperitoneally or subcutaneously remained in the vicinity of the injection site and uptake in the liver or spleen was much lower.^96,381^ One study also observed migration to perithymic lymph nodes of mice after intraperitoneal administration of ^64^Cu-labeled T cells.^117^ Key features and advantages of PET for imaging during adoptive cell therapies, for example, its high sensitivity, its utility to determine cell uptake kinetics and their dependence on tumor size and vascularization, were already apparent in very early preclinical studies. For example, despite the very short half-life of ^11^C (20 min), activated murine NK cells labeled with [^11^C]methyl iodide and injected in close proximity to the tumors accumulated 5× more in the tumors than similarly administered control cells, and heterogeneous uptake was observed particularly in larger tumors.^150,151^^89^Zr-labeled NK cells have been investigated preclinically for the treatment of hematological malignancies but low trafficking to the bone marrow was observed.^134^ The use of 5-[^124^I]iodo-2-deoxyuridine ([^124^I]IdU) allowed the visualization of tumor antigen-specific T cells in tumors with as little as 0.3 kBq/10^6^ cells.^183^ [^111^In]In-oxine and [^89^Zr]Zr-oxine-labeled γδ-T cells have been shown to accumulate in tumors in preclinical models;^81,95^ this accumulation was dependent on the presence of a functional γδ-TCR^67^ and increased after treatment with a liposomal aminobisphosphonate drug.^81^ Similar increased uptake in tumors expressing a specific antigen was observed in mice with ^89^Zr-labeled CAR T cells^75,218^ (Figure 23) and other tumor antigen-specific cytotoxic T cells.^76,82^ Tracking CAR T cells with ^68^Ga-oxine has also been performed. The half-life of ^68^Ga was, unsurprisingly, too short to observe the accumulation of CAR T cells in solid tumors, but for short-term tracking, the results were the same as with ^89^Zr-labeled cells and the radiation doses much lower.^71^ Overall, studies of radiolabeled lymphocytes in oncology show that adoptively transferred lymphocytes expressing tumor-specific antigens can indeed accumulate in tumors, provided the specific tumor antigens are accessible.

Preclinical studies using DCs labeled with [^111^In]In-oxine or [^18^F]SFB showed that local administration of DCs leads to accumulation in the draining lymph nodes, whereas intravenous administration leads to a similar distribution pattern to that of lymphocytes, i.e. initial accumulation in the lungs followed by liver and spleen.^59,152,188^ Results using ^111^In-, ^99m^Tc-, or ^64^Cu-labeled DCs in humans exhibited more variability but the overall picture is one where migration of DCs to the lymph nodes depends on the route of administration, with local routes (intralymphatic, intradermal, subcutaneous) showing much more uptake in lymph nodes than after intravenous administration.^58,112,188,339,382^ Interestingly, mature DCs were found to remain trapped in the lungs of patients much longer than nonmatured DCs after intravenous administration, and the use of ^64^Cu-PET enabled detection of as few as 7000 cells per lymph node.^188^

In other oncological applications, tumor cells have been radiolabeled to study metastasis in preclinical models, examining for example the role of protein kinase C (PKC) or surface sialylation in the accumulation of metastatic cells in the liver^383,384^ or the tropism of different tumor cell lines to the liver and lungs.^385^ However, tumor metastasis is generally a slower process than the radioactive decay of the most commonly used radionuclides for cell labeling. For such studies, it is nowadays preferable to use reporter gene imaging systems, which allow repeated imaging of cells over much longer periods (see reviews by Iafrate et al. and Serganova and Blasberg^2,386^).

Fewer studies, however, have attempted to correlate the therapeutic efficacy with the degree of cell uptake as determined by nuclear imaging. In patients, the combination of ^111^In-labeled TILs with cyclophosphamide (an immunosuppressant using in cancer chemotherapy) resulted in higher tumor accumulation of TILs than without cyclophosphamide, and clinical response was observed in 38% of the patients who showed TIL uptake in tumors, but in none of the patients who showed no uptake in tumors.^387^ Preclinically, ^111^In-labeled tumor antigen-specific cytotoxic T cells were shown to accumulate in higher numbers in the tumors of lymphodepleted mice compared to nondepleted mice, and this combination also resulted in a greater therapeutic effect (Figure 23).^64^ Similarly, ovalbumin-specific T cells labeled with ^89^Zr accumulated in ovalbumin-expressing tumors and induced tumor shrinkage in mice.^76^ In the future, the uptake of labeled cells at the target location, determined by quantitative imaging methods and particularly PET, may become a key clinical end point in trials of cell therapies.

Finally, radiolabeling and imaging therapeutic cells could also be an additional safety measure in the clinic, particularly for novel adoptive cell-based therapies. There are notable reports of engineered autologous T cells attacking healthy tissue and resulting in severe toxicity and even patient deaths, either because the target antigen was also expressed on nontumor cells (e.g., liver toxicity in the case of carbonic-anhydrase-IX (CAIX)-targeting CAR T cells attacking CAIX expressed on bile duct epithelial cells,^388^ and pulmonary toxicity due to the recognition of tumor antigen ERBB2 on lung epithelial cells^389^) or because of unexpected cross-reactivity of the T cells with an antigen expressed on a nontarget organ (e.g., cardiotoxicity of MAGE A3-specific T cells cross-reacting with the muscle protein titin^390,391^). Nuclear imaging of adoptive cell therapies could detect the accumulation of cells in nontarget locations and thus provide an early warning of impending toxicity and allow mitigating measures (e.g., immunosuppression) to be taken rapidly.

## 7 Conclusions and Future Perspectives

Cell labeling and tracking using nuclear medicine techniques has been used for decades in both preclinical and clinical studies. With the advent of novel and highly efficacious cell-based therapies such as those based on CAR technology, as well as new immune cell types (e.g., natural killers T cells, γδ-T cells, dendritic cells), there is an increasing need to develop novel methods to image the fate of these cells after administration in patients, to help understand under what circumstances they may be efficacious or give rise to toxic side-effects.

In this Review, we have reviewed the different chemical methods available to date for directly radiolabeling cells. Compared to indirect methods, direct radiolabeling has specific advantages (e.g., avoiding genetic modification) and disadvantages (e.g., relatively short-term imaging, potential of radiolabel loss over time), that we have discussed. Overall, direct cell radiolabeling remains the most widely used method to track cells in the clinical setting. Therefore, we expect that direct radiolabeling will continue to play a key role in the development and evaluation of cell-based therapies, although we note that clinical translation of these techniques is significantly slower nowadays than in the early days of their development. Taking into account the current regulatory frameworks, and to improve the clinical translation of new direct radiolabeling techniques, researchers need a clear understanding of these regulatory hurdles from the early stages of their development. Cell-based therapies are more complex in their production and distribution than patient-based white blood cells and hence may be limited in how and when they can be radiolabeled and imaged. In addition, improved radiobiological and functional assessment of the impact of radiolabeling on the cells of interest should always be implemented to ensure confidence in image interpretation. We also highlight the importance of understanding the fate of the radionuclide after cell radiolabeling, in vitro and in vivo, as this will allow efficient assessment of the success of cell tracking studies. This is particularly important when using ionophore-based methods that may result in the leakage of free radionuclides, such as ^64^Cu, that share accumulation in organs and excretion pathways with those of the cells themselves (for example, liver and spleen), or when using phospholipid-based radiolabeling, as phospholipids may exchange between different cells.

In our view, the more exciting development in this field is the advent of total-body PET, a new scanner technology that promises a remarkable 40-fold increase in sensitivity.^31^ The significance of this technology in the future of cell tracking studies should not be underestimated: it should allow significantly lower levels of radioactivity per cell, allowing tracking of radiosensitive cells, tracking different cell types, imaging multiple radiotracers in the same patient using short-lived radionuclides, and tracking directly labeled cells for much longer periods of time compared to current PET technology. Another area in which these radiolabeling technologies can play a significant role in the development of cell-based therapies is in the new field that is evaluating how pharmacological interventions can modify cell trafficking, aiming to improved therapeutic outcomes and safety profiles. We hope that the different direct radiolabeling strategies reviewed and outlined in this review, as well as the discussion of their preclinical and clinical applications to date, will enable scientists from different areas to effectively choose the most appropriate radiochemical method for their cell-tracking studies.

## Abbreviations Used

ATP: adenosine triphosphate
ATOX-1: antioxidant protein 1
CAR: chimeric antigen receptor
CTR-1/2: high affinity copper uptake protein 1/2
CXCR4: chemokine receptor type 4
DBCO: dibenzocyclooctyne
DEDTC: diethyldithiocarbamate
DMT-1: divalent metal transporter 1
DMDTC: dimethyldithiocarbamate
DNA: deoxyribonucleic acid
DOTA: 2,2′,2″,2‴-(1,4,7,10-tetraazacyclododecane-1,4,7,10-tetrayl)tetraacetic acid
DPDTC: dipropyldithiocarbamate
FACS: fluorescence-activated cell sorting
FOV: field of view
GFP: green fluorescent protein
HMGB-1: high mobility group box 1 protein
HMPAO: hexamethylpropylene amine oxime
LE: labeling efficiency
MR: magnetic resonance
NHS: *N*-hydroxysuccinimide
NIS: sodium iodide symporter
NOTA: 1,4,7-triazacyclononane-1,4,7-triacetic acid
PTSM: pyruvaldehyde bis(*N*^4^-methylthiosemicarbazone)
RBC: red blood cells
RFP: red fluorescent protein
SiNPs: silica nanoparticles
SLC30A10: solute carrier family 30 member 10
SPIONs: superparamagnetic iron oxide nanoparticles
TETA: 1,4,8,11-tetraazacyclotetradecane-1,4,8,11-tetraacetic acid
TRPML-1: transient receptor potential mucolipin 1
WBC: white blood cells
ZIP-14: zinc-import protein 14
